# msBayesImpute as a versatile framework for addressing missing values in biomedical mass spectrometry proteomics data

**DOI:** 10.1038/s42004-026-02106-3

**Published:** 2026-07-07

**Authors:** Jiaojiao He, Barbara Helm, Franziska Gödtel, Katharina Büchner, Marcel Schilling, Marc A. Schneider, Laura V. Klotz, Jana Braunger, Hauke Winter, Britta Velten, Ursula Klingmüller, Junyan Lu

**Affiliations:** 1 Institute for Computational Biomedicine, Medical Faculty Heidelberg, Heidelberg University, Heidelberg, Germany; 2 System Biology of Signal Transduction, German Cancer Research Center (DKFZ), Heidelberg, Germany; 3 Translational Lung Research Center Heidelberg (TLRC), Member of the German Center for Lung Research (DZL), Heidelberg, Germany; 4 Translational Research Unit, Thoraxklinik at Heidelberg University Hospital, Heidelberg, Germany; 5 Department of Thoracic Surgery, Thoraxklinik at Heidelberg University Hospital, Heidelberg, Germany; 6 Biological Data Science, Center for Organismal Studies (COS), Heidelberg University, Heidelberg, Germany; 7 Interdisciplinary Center for Scientific Computing (IWR), Heidelberg University, Heidelberg, Germany

**Keywords:** Proteomics, Computational chemistry

## Abstract

Advancements in mass spectrometry (MS) technologies have significantly improved the ability to quantify proteins and analyse their modifications. However, MS-based proteomics datasets frequently encounter missing values due to a complex interplay of missing at random (MAR) and missing not at random (MNAR) mechanisms. Such missing data can result in information loss and biased outcomes in data pre-processing, as well as subsequent analyses and interpretations. Few approaches effectively address both MAR and MNAR, and those that do often necessitate manual tuning of mixture percentages between them or rely on two-group experimental designs. Therefore, we developed msBayesImpute, an innovative computational method that integrates Bayesian factorization with probabilistic dropout models. We evaluated msBayesImpute against several popular imputation methods using both simulated missing values and those generated through a dilution series experiment on samples from lung cancer patients. Our comprehensive benchmark demonstrated superior performance in reconstructing missing values, estimating normalization factors, identifying differentially expressed proteins and predicting outcomes with machine learning models across varying levels of missingness and sample sizes. Notably, msBayesImpute does not require predefined experimental designs and is scalable to large-scale studies. This versatility positions msBayesImpute as an effective and robust tool for enhancing the utility of MS datasets in biological research.

## Abstract (web summary)

Mass spectrometry-based proteomics often suffers from missing data, leading to biased analyses and interpretations. Here, the authors introduce msBayesImpute, a Bayesian factorization method that effectively addresses both MAR and MNAR mechanisms, outperforming existing imputation techniques and enhancing the utility of MS datasets in large-scale biological research.

## Introduction

Proteins play a crucial role in the majority of biological processes. Thus, their identification and quantification are essential for understanding physiological and pathological mechanisms, as well as identifying disease biomarkers and therapeutic targets^1,2^. Proteomics also complements other omics data types, such as genomics, transcriptomics, epigenomics, etc., in studying complex biological systems and diseases^3,4^. Recent advancements in mass spectrometry (MS) technologies, especially the label-free acquisition methods, have enabled highly accurate and high-throughput identification and quantification of proteins and their modifications in biological samples^5–7^. Nevertheless, MS-based proteomics data often suffer from missing values due to the complex interplay of missing at random (MAR) and missing not at random (MNAR) patterns^8,9^.

In MS proteomics, MAR values occur independently of protein abundance and primarily due to technical factors such as sample loss, ionization inefficiency, or errors in data processing^10–12^. In contrast, MNAR values are abundance-dependent and typically arise when proteins are present near or below the detection limit of instruments^13^. Both patterns often co-exist in datasets, with MNAR being the more prevalent source of missing values^10^. These missing values decrease statistical power and reduce reproducibility across experiments. If not properly handled, the missing values also introduce bias in statistical analysis, such as normalization, differential expression, and lead to the incorrect interpretation of the data. On the other hand, many popular statistical and machine learning tools, such as principal component analysis (PCA), regularised multiple linear regression, support vector machines, etc., only work with complete datasets without missing values.

Besides enhancing instrument sensitivity and sample processing, a common approach for addressing missing values in MS proteomics data is imputation, which aims to replace missing values with reasonable substitute values. Numerous statistical methods for missing value imputation (MVI) have been developed to date. General-purpose MVI methods, K-nearest neighbours (KNN)^14^, random forest-based (missForest)^15^, Bayesian PCA (BPCA)^16^, and multi-omics factor analysis (MOFA)^17^ are commonly employed to address datasets containing missing values. These methods reconstruct missing values based solely on observed data, thereby implicitly assuming the MAR mechanism. This assumption can result in biased estimation in MS proteomics data, particularly when the MNAR portion is significant^13^. To manage the MNAR mechanism in MS proteomics data, left-censored imputation methods have been proposed, such as Quantile Regression Imputation of Left-Censored data (QRILC)^18^, which uses quantile regression to sample from a normal distribution near the lower detection limit, and MinDet^18^, which replaces missing values with either the dataset’s minimum value or the minimum per sample. However, these methods inherently introduce underestimation errors by disregarding the MAR pattern and generally exhibit inferior imputation accuracy, as they do not leverage the underlying data correlation structure to aid imputation like other MAR imputation methods.

Efforts have been made to model both MAR and MNAR portions in MS proteomics data to enhance imputation accuracy. Gatto and Lilley introduced a mixed imputation method within the R package MSnbase, combining a MAR imputation function, such as KNN, and an MNAR function, such as MinDet^19^. However, users must specify the subset of data following the MNAR mechanism, which is typically unknown. Currently, some approaches can automatically identify protein subsets adhering to MAR or MNAR mechanisms, such as MsImpute^20^, and detection probability curve (DPC) modelling incorporated into differential expression testing in the limpa R package^21,22^. Recently, proteomics imputation using self-supervision (PIMMS)^23^ was proposed, aiming to address MS proteomics data imputation with a self-supervised deep-learning framework capable of learning unique missing value patterns inherent in MS proteomics data. Nevertheless, this deep-learning-based method necessitates a considerably large sample size (*n* > 500) to surpass traditional imputation techniques like BPCA, which is often impractical in standard biomedical studies. Given the constraints of the aforementioned methods, there remains a demand for a more flexible and precise MVI method suitable for MS data from typical biological or biomedical studies.

Probabilistic dropout analysis (proDA), which explicitly models the relationship between protein abundance and missing probability using a Bayesian approach, has been shown to enhance differential expression analysis in label-free MS data by effectively accounting for mixed MAR/MNAR patterns^24^. Drawing inspiration from proDA and BPCA, here we introduce msBayesImpute, a novel MVI method that integrates probabilistic dropout models into Bayesian matrix factorization to address the unique missing value patterns in label-free MS proteomics data in a data-driven manner.

## Results

### Overview of msBayesImpute

**Fig. 1:**
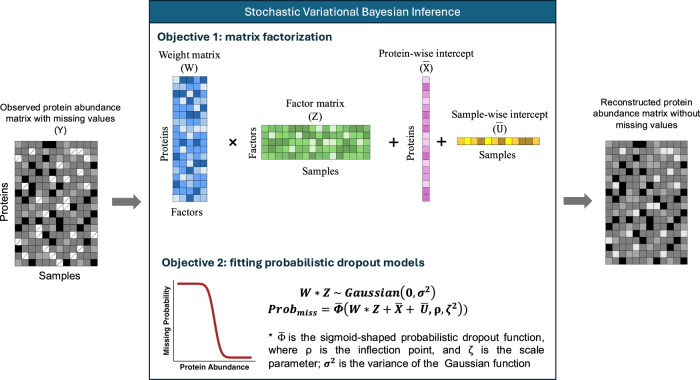
Overview of msBayesImpute. An MS-based proteomics dataset with missing values is decomposed into four components: weight matrix, factor matrix, protein-wise intercept, and sample-wise intercept. msBayesImpute integrates a probabilistic dropout function $\bar{\varPhi }$ to model abundance-dependent missingness and jointly optimizes these components through stochastic variational inference. The reconstructed matrix without missing values can be directly used in downstream analyses with standard statistical or machine-learning tools.

msBayesImpute integrates Bayesian matrix factorization with a probabilistic dropout model to impute missing values in protein abundance matrices derived from label-free MS (Fig. 1). By combining the strengths of general-purpose methods such as BPCA (which shares information across features and samples by leveraging covariance structures) with left-censored approaches that address MNAR patterns, msBayesImpute captures both the correlation structure of the data and the abundance-dependent dropout typical of proteomics. Its regularized Bayesian framework suppresses noise while retaining biologically meaningful variation, thereby reducing the risk of false discoveries. Implemented with stochastic variational inference (SVI), the method achieves high computational efficiency and scalability. The detailed description of the architecture and implementation of msBayesImpute can be found in the “Methods”.

To facilitate broad adoption, msBayesImpute is available in both R and Python, requires no complex parameter tuning or predefined experimental design, and can be accessed through an interactive Shiny application. This ease of use, combined with its ability to model complex missingness mechanisms, makes msBayesImpute suitable for diverse applications, including exploratory data analysis, normalization, differential expression testing, dynamic modeling of signaling networks, and machine learning tasks. Together, these features position msBayesImpute as a versatile and robust tool for enhancing the reliability and interpretability of label-free MS proteomics in biomedical research.

### msBayesImpute accurately predicts protein-specific dropout functions

In real proteomics datasets, the true dropout functions, which can be visualized as sigmoid-shaped curves describing the relationship between protein abundance and the probability of missingness^24^, are unknown, yet accurately estimating them is essential for reliable imputation. Moreover, proteins often exhibit distinct dropout behaviors depending on their physical and chemical properties, further complicating inference when data are limited. To address this, msBayesImpute employs an empirical Bayesian framework to estimate protein-specific dropout functions. In cases where data are sparse—for example, when sample sizes are small or when a protein has too many missing values—the model adaptively shrinks the protein-specific estimates toward global priors derived from all observed data points, thereby ensuring stable and biologically meaningful parameter inference.

To assess the ability of msBayesImpute to recover protein-specific dropout functions, we first used fully synthetic data with known parameters. Complete data matrices were generated by multiplying Gaussian-distributed weight and factor matrices, representing log-transformed protein abundance values that typically follow an approximately Gaussian distribution. Each dataset comprised 5000 proteins and 10 latent factors, with a median protein abundance of 20. To evaluate the effect of sample size, we simulated datasets containing 6, 12, 20, 100, 200, 500, and 1000 samples. Missing values were introduced at a rate of 30% using a probabilistic dropout function that assigns protein-specific missingness patterns. Details regarding synthetic data generation are described in the “Methods” section.

**Fig. 2:**
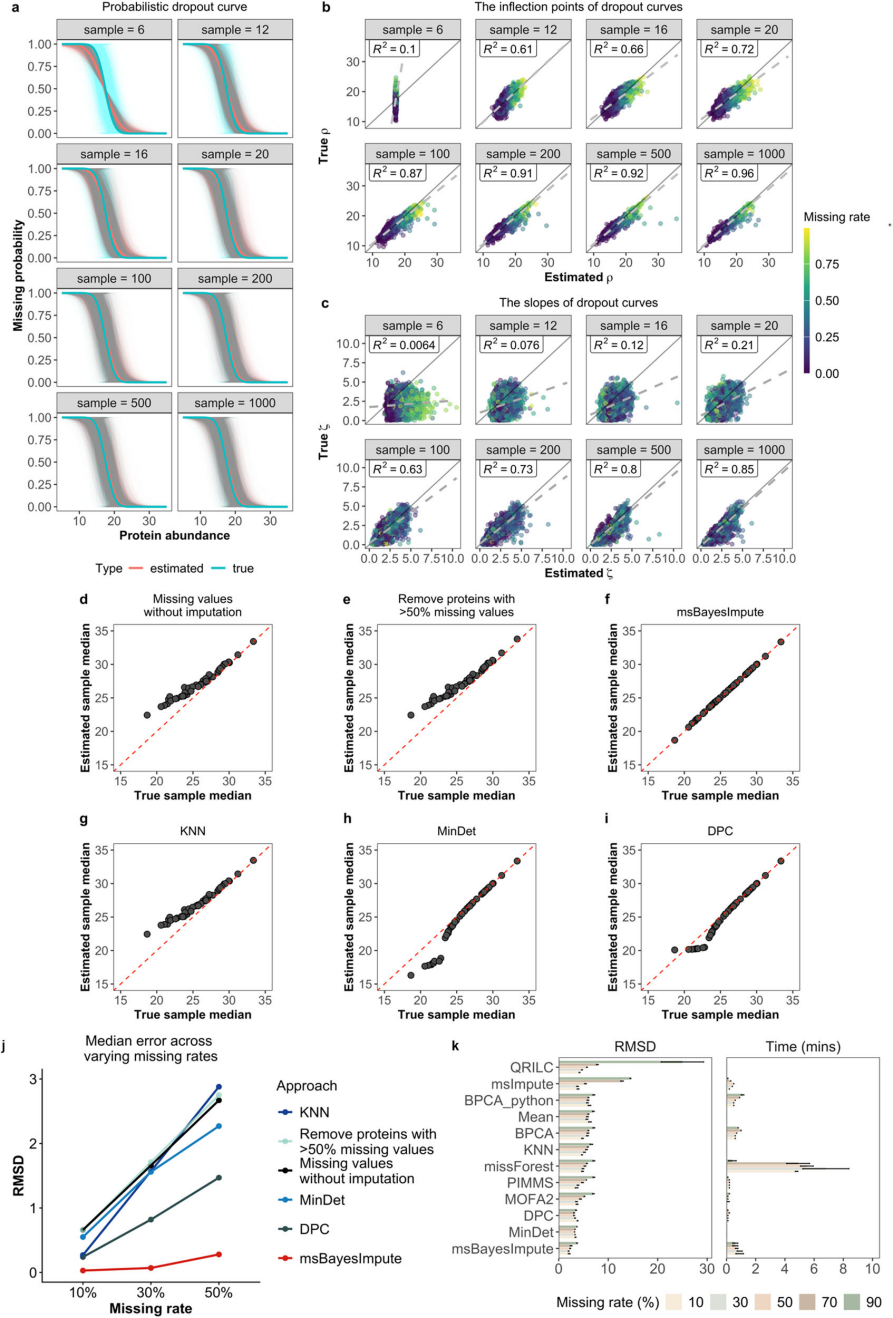
Benchmarking msBayesImpute using synthetic data. **a** Comparison of ground truth dropout curves (cyan) and curves inferred by msBayesImpute (red). Solid lines represent the average curve, and shaded lines represent individual protein-specific curves. **b**, **c** Scatter plots comparing true versus estimated dropout parameters (${{\rm{\rho }}}$ and ${{\rm{\zeta }}}$), which define the location and shape of the dropout curves. *R*² values indicate the reproducibility of parameter estimation. Each point represents a protein, with color indicating its simulated missing-value probability. Solid lines correspond to the diagonal, and dashed lines represent the linear regression lines. **d**, **e** The *x*-axis represents the sample-wise medians derived from ground truth data without missing values, and the *y*-axis represents the estimated medians directly from matrices with ~30% missing values. In (**e**), proteins with >50% missingness were excluded before median calculation. **f–i** Same analysis as in (**d**), but medians were estimated from imputed complete matrices using msBayesImpute, KNN, MinDet, and DPC. **j** Median estimation errors across datasets with 10, 30, and 50% missing values, comparing msBayesImpute to other approaches. **k** Root mean square deviation (RMSD) between the imputed and simulated ground truth values. The bars show the averaged RMSD over 5 random repetitions, and the error bars indicate the 95% confidence interval (CI). The runtimes of each method were shown under the same conditions.

msBayesImpute accurately recovered both the shape and parameters of protein-specific dropout functions—namely the inflection point ($\rho$) and slope ($\zeta$)—across all missing rates and sample sizes, except for the smallest dataset (*n* = 6) (Fig. 2a–c and Supplementary Fig. 1a). In this case, due to insufficient observations, the model automatically reverted to a global dropout curve rather than estimating protein-specific functions. A substantial improvement in imputation accuracy was observed when increasing the sample size from 6 to 12 (Supplementary Fig. 1b). While the inflection point ($\rho$) can already be reliably recovered with 12 samples, accurate estimation of the slope ($\zeta$) remains more challenging and requires larger sample sizes. Performance improves between 20 and 100 samples markedly, but only modestly beyond 100 samples, indicating that msBayesImpute can be effectively trained on relatively small cohorts. This contrasts with many deep learning–based approaches, which typically require hundreds to thousands of samples. Computational time remained relatively stable from 6 to 500 samples (ranging from seconds to approximately 3 min), but increased substantially at 1000 samples (approximately 12 min) (Supplementary Fig. 1b). A non-linear increase in run time for larger datasets is expected, as more latent factors and posterior parameters need to be estimated during model training, increasing both computational complexity and memory usage. This highlights a potential direction for future optimization of msBayesImpute for very large-scale datasets through mini-batch training and parallelization.

To further evaluate robustness under extreme missingness, we generated datasets with 50 samples and missing rates ranging from 10 to 90%. Imputation error (RMSD, root mean squared deviation) increased sharply when the missing rate rose from 70 to 80%, indicating that msBayesImpute performs robustly up to approximately 70% missingness (Supplementary Fig. 1c).

### msBayesImpute improves normalization accuracy

Most normalization methods used in MS proteomics—such as median normalization or variance stabilizing transformation (VSN)^25^—were originally developed for gene expression data, where missing values are rare. These methods assume that the overall distribution of feature values is comparable across samples, with similar medians or means. In proteomics, however, MNAR values violate this assumption: low-abundance proteins are more likely to drop out, which inflates sample medians and biases the size factors used for normalization. To quantify this effect, we simulated datasets with substantial variability in sample-specific intercepts (standard deviation = 3), representing differences in total protein abundance across 50 samples with ~30% missing values. Sample medians computed from incomplete data deviated markedly from the ground truth, particularly for samples with low total protein abundance (Fig. 2d). This non-uniform bias propagates into normalization and compromises downstream analyses. Notably, filtering proteins with >50% missingness, a common practice in data preprocessing, did not alleviate the bias (Fig. 2e).

We next benchmarked msBayesImpute against representative MAR-, MNAR-, and mixture-based methods (KNN, MinDet, and DPC, respectively) in recovering true sample median abundances (Fig. 2f–i). While these approaches partially reduced the bias, their reconstructed medians still deviated systematically and non-uniformly from the ground truth. In contrast, msBayesImpute consistently reconstructed medians that closely matched the true values. Across all tested missingness levels (10, 30, 50%), it achieved the lowest and most stable normalization error (Fig. 2j). Together, these results show that msBayesImpute accurately recovers sample-wise size factors even under substantial dropout, thereby providing a more robust foundation for downstream analyses such as differential expression.

We further evaluated whether an impute-first or normalize-first strategy should be adopted when benchmarking MVI methods. Median normalization was used as the reference procedure. Although normalization before imputation is common in practice, it can introduce bias in the presence of MNAR missingness, as shown above. Using synthetic datasets, we systematically compared both strategies across multiple missingness levels. msBayesImpute consistently benefited from an impute-first strategy, although the improvement was modest (Supplementary Fig. 1d). In contrast, PIMMS, missForest, and BPCA showed substantial improvements under a normalize-first strategy, while QRILC exhibited a smaller but consistent advantage. Accordingly, these four methods were evaluated using a normalize-first approach, whereas all other methods, including msBayesImpute, were applied in an impute-first framework.

### Benchmarking the imputation accuracy and computational efficiency with synthetic data

We next benchmarked msBayesImpute against ten commonly used MVI methods, under varying missing rates (10, 30, 50, 79, 90%; five simulated datasets each) with 50 samples per dataset. The compared methods included deterministic minimum (MinDet)^18^, quantile regression imputation for left-censored data (QRILC)^18^, BPCA^16^, MOFA^17^, missForest^15^, KNN^14^, PIMMS^23^, MsImpute^20^, DPC from limpa package^21,22^, and protein-wise mean imputation. BPCA and missForest are available in both R and Python implementations. However, the current Python implementation of missForest is outdated and significantly slower than the R version and therefore not included in the benchmarking. Among all methods msBayesImpute showed the best performance, yielding the lowest RMSD and therefore the highest imputation accuracy, with MinDet, DPC, and MOFA ranking next (Fig. 2k). MsBayesImpute showed a slow increasing of errors up to 70% missing values (Supplementary Fig. 1c). MOFA2 performed well at low missingness (10%) but degraded sharply as missingness increased, a trend also observed for PIMMS, missForest, KNN, BPCA, MsImpute, and QRILC. In contrast, msBayesImpute, MinDet, DPC, protein-wise mean, and BPCA in Python implementation displayed relatively stable RMSD across missingness levels. All methods were accessible in *R*, except for PIMMS, which was only available in Python. In addition, the runtime of BPCA in Python and R was comparable, but the *R* implementation achieved slightly higher accuracy than Python. Therefore, the *R* version of BPCA was retained for subsequent benchmarking. Notably, missForest was computationally inefficient for high-dimensional matrices, while msBayesImpute achieved completion in ~1 min (Fig. 2k). By comparison, MinDet, QRILC, KNN, and feature-wise Mean completed each job within seconds.

We further evaluate the impact of sample size on imputation accuracy and computational efficiency by varying the sample size from 6 to 1000 while fixing the missing rate at 30%. The imputation accuracy of msBayesImpute increased with larger sample sizes, as expected (Supplementary Fig. 1b). While the run time remained stable till 200 samples (less than 3 min), it increased significantly with 1000 samples and is not as computationally efficient as other methods, e.g., PIMMS (Supplementary Fig. 1e). However, msBayesImpute still outperformed all other methods in terms of imputation accuracy and the run time is in an acceptable range with 1000 samples (~ 10 min, similar to DPC) (Supplementary Fig. 1e).

### Benchmarking on semi-synthetic HeLa cell line proteomics data highlights the robust and accurate performance of msBayesImpute

To evaluate msBayesImpute on real-world data and benchmark it fairly against existing methods, we used a published HeLa cell line proteomics dataset that was originally employed to develop and assess PIMMS, a state-of-the-art deep learning–based imputation method^23^. The dataset consisted of 564 HeLa runs acquired on a Q Executive HF-X Orbitrap instrument during continuous MS quality control. In addition, a smaller dataset with 50 samples was generated to test performance dependence on sample size^23^ (Supplementary Data 1). For benchmarking, missing values were introduced using the approach of ref. ^8^, which has been widely applied in proteomics benchmarking studies^11,13,23,26^. This method provides precise control over both the overall missing rate and the mixture of MNAR and MAR patterns. In the MNAR setting, low-abundance values are preferentially removed to mimic left-censoring near the detection limit, whereas in the MAR setting, values are masked independently (see “Methods”). Based on the smaller (*N* = 50) and larger (*N* = 561) datasets, we generated a series of data matrices with MNAR proportions of 0, 25, 50, 75, and 100%, where the 100% means the artificial missing values are purely MNAR. Proteins with greater than 75% missing values and samples with greater than 50% missing values were filtered out to reproduce the workflow used by ref. ^23^.

**Fig. 3:**
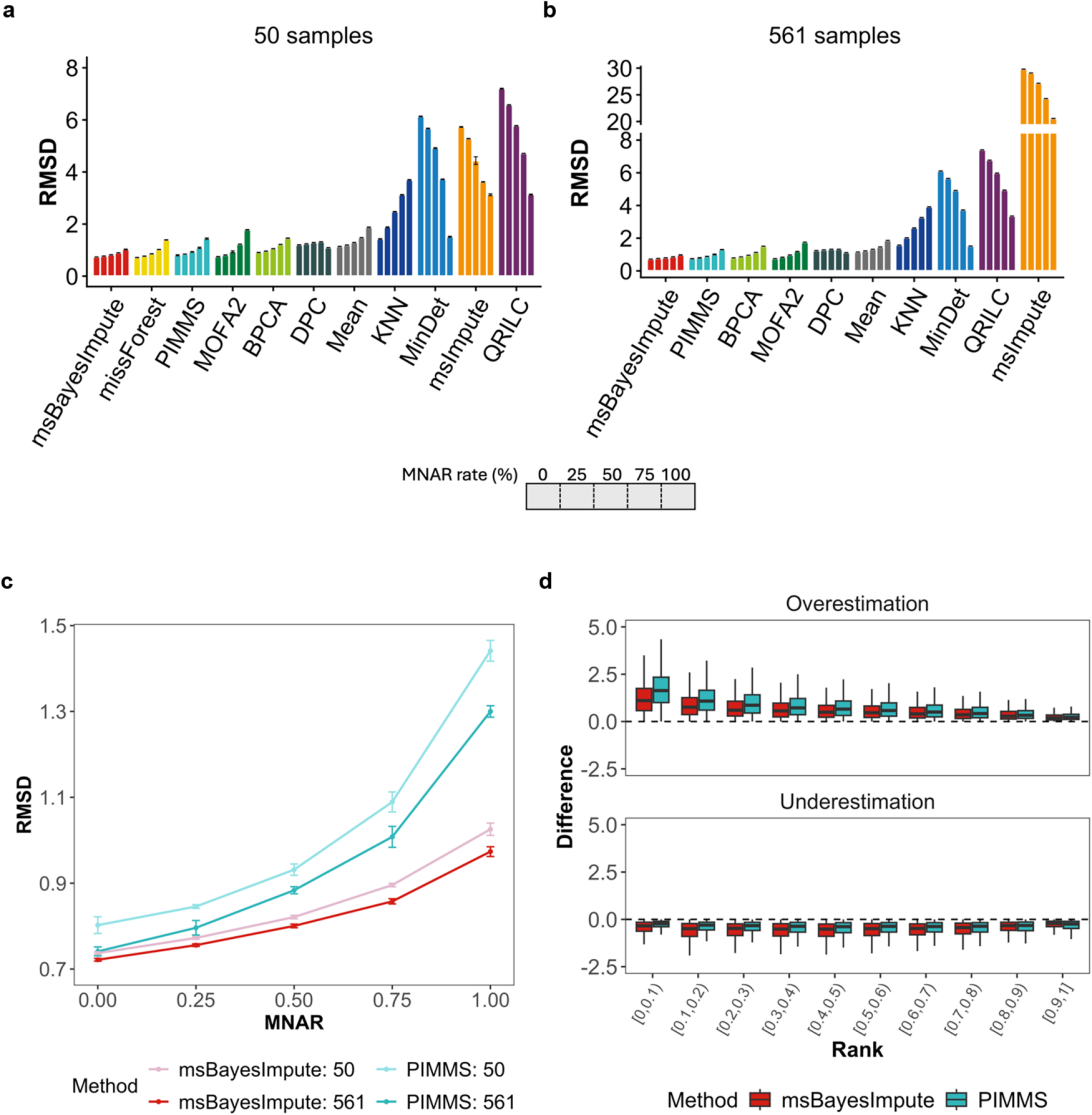
Benchmarking msBayesImpute on semi-synthetic published HeLa cell line proteomics data with varying MNAR proportions. **a** Reconstruction accuracy of eleven imputation methods across datasets with MNAR proportions ranging from 0 to 100% (25% increments from left to right), based on RMSD between imputed and ground truth values in the smaller dataset (*N* = 50). The error bars indicate the 95% CI. **b** Performance of the same methods on the larger dataset (*N* = 561), excluding missForest, which failed to converge. **c** Direct comparison of msBayesImpute and PIMMS across both small (*N* = 50) and large (*N* = 561) datasets, showing RMSD as a function of MNAR proportion with 95% CI indicated as error bars. **d** Protein-level imputation error analysis comparing msBayesImpute and PIMMS using the small dataset (*N* = 50), with over- and underestimation quantified across ten ranked protein abundance groups. difference = imputed values — true values, where Difference > 0 indicates overestimation and Difference < 0 indicates underestimation.

Across both small and large datasets, msBayesImpute demonstrated the best reconstruction accuracy and maintained stable performance across all MNAR levels. Methods based on MAR assumptions—including missForest, MOFA, PIMMS, BPCA, KNN, and mean imputation—showed steadily deteriorating accuracy as the MNAR proportion increased (Fig. 3a, b). DPC also showed stable performance in the MNAR range of 0–75% with a slight improvement under 100% MNAR. On the contrary, methods explicitly modelling MNAR, such as QRILC and MinDet, as well as mixture models like MsImpute, showed improved performance with increasing MNAR content but remained substantially less accurate overall.

In the small dataset (*N* = 50), missForest performed second best but was computationally prohibitive on the large dataset (*N* = 561), failing to converge within five hours. PIMMS performed second best on the large dataset, but its accuracy dropped sharply when the MNAR proportion increased, and it exhibited a large performance gap between small and large sample sizes (Fig. 3c). By contrast, msBayesImpute maintained stable accuracy across both scenarios, and the run time is on par with the other more computationally demanding methods (Supplementary Fig. 2a, b).

To further dissect error sources, proteins were ranked into ten abundance groups, and over- and underestimation errors were quantified (Fig. 3d). Overestimation was most pronounced among low-abundance proteins, where msBayesImpute showed marked improvements over PIMMS across all groups. Although msBayesImpute exhibited slightly higher underestimation than PIMMS on average, the difference was minimal.

Together, these results show that msBayesImpute delivers consistently strong performance on semi-synthetic HeLa proteomics data across varying MNAR proportions and sample sizes, underscoring its robustness and suitability for diverse experimental settings

### Validation of msBayesImpute using serial dilution experiments on primary lung cancer tissues

**Fig. 4:**
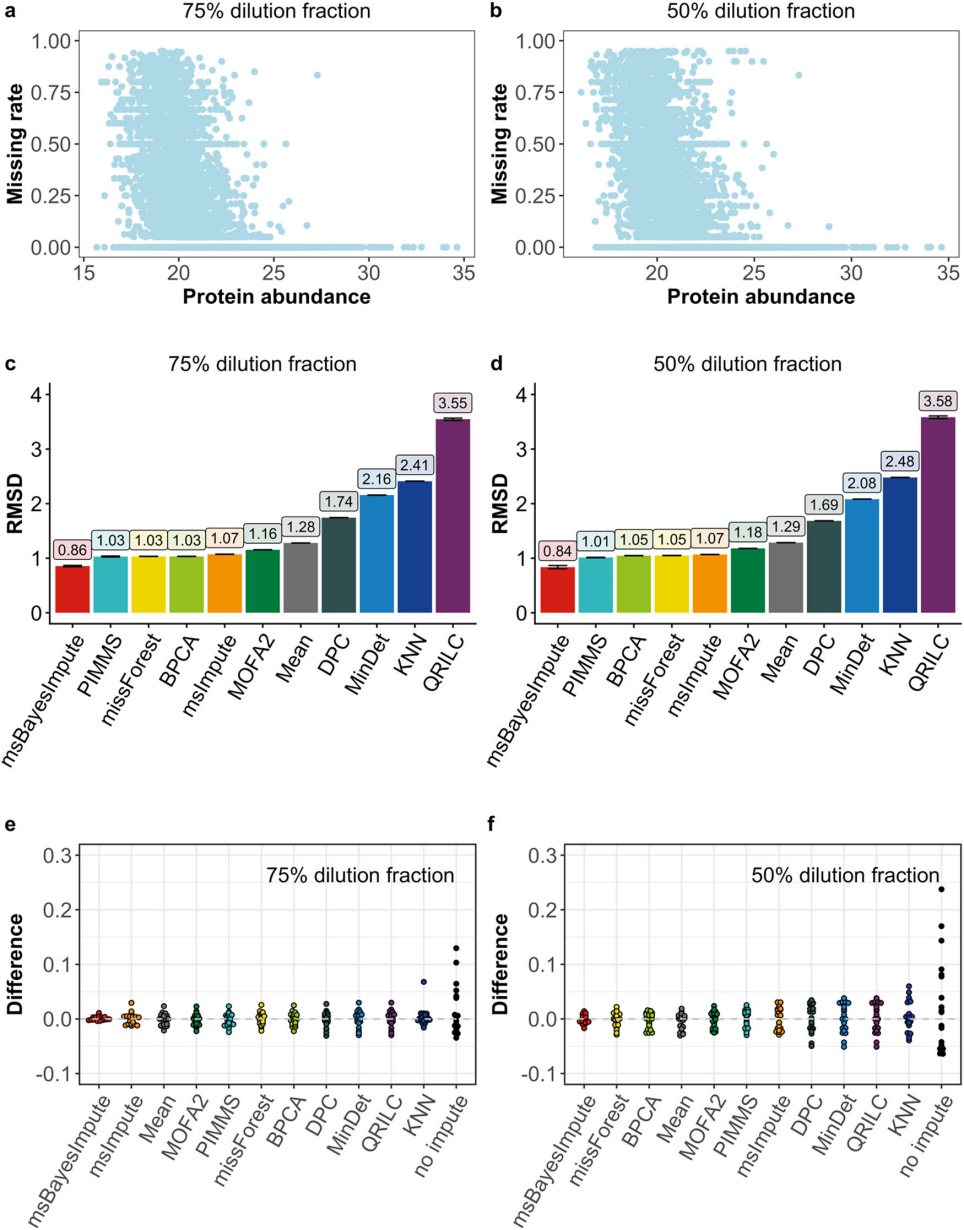
Benchmarking msBayesImpute using serial dilution experiments. **a**, **b** Inverse relationship between protein-specific missing rate and average protein abundance of the ground truth values observed in the dilution datasets. **c**, **d** Reconstruction accuracy of eleven imputation methods, evaluated on unbiased validation data generated from serial dilutions of the original sample concentration (75 and 50% relative to undiluted controls, *N* = 20). RMSD between imputed values and ground truth values from the 100% (undiluted) dataset was used as the performance metric. Error bars indicate 95% CI. **e**, **f** Errors in estimating sample-wise medians, calculated by comparing values from the original undiluted dataset (100%) with those from imputed or incomplete datasets of the 75 and 50% dilution fractions, and centered at zero.

Most existing MVI tools for mass-spectrometry proteomics have not been tested on truly experimental benchmarking data. This is largely because the ground truth for missing values is unknown, and any synthetic introduction of MNAR values inevitably relies on assumptions that may bias the evaluation toward certain methods. To minimize such bias, we employed serial dilution experiments to generate missing values with experimentally defined ground truth. Specifically, lysates of matched tumor and tumor-free tissue samples from 10 lung adenocarcinoma patients were prepared and utilized to establish three dilution fractions: 100 (undiluted), 75, and 50% of the original input concentration (Supplementary Data 2). Proteomic data from patient tissue samples are a particularly challenging matrix, often containing many missing values, making reliable information retrieval especially important. Values observed in the undiluted samples but absent in the diluted samples could therefore be regarded as ground truth. The experimentally generated missing values displayed an inverse relationship between protein abundance and dropout probability, consistent with a sigmoid-shaped dropout curve and in line with the assumptions validated in our synthetic and semi-synthetic benchmarking (Supplementary Fig. 2c, d and Fig. 4a, b).

We next compared msBayesImpute with ten other MVI methods. Based on the RMSD between imputed and ground-truth values, msBayesImpute achieved the highest reconstruction accuracy, reducing error by approximately 20% relative to the second-best method, PIMMS, followed by missForest, BPCA, MsImpute, and MOFA (Fig. 4c, d). Incorporating a two-group experimental design improved the performance of MsImpute, particularly compared to the semi-synthetic HeLa benchmark, where no experimental design was considered (Fig. 3a, b). The relative ranking of the remaining methods was largely consistent with the semi-synthetic HeLa results, with protein-wise mean imputation, DPC, MinDet, KNN, and QRILC performing the worst. In terms of computational efficiency, msBayesImpute, BPCA, and missForest required approximately 1–2 min to complete on datasets of this size, whereas the other methods finished within seconds (Supplementary Fig. 3a, b).

We further evaluated the ability of msBayesImpute to recover true sample-wise abundance, represented by the sample median, which is critical for accurate normalization. Notably, variability in estimation error is more consequential for normalization than absolute differences alone. Medians derived from msBayesImpute-imputed data were tightly concentrated around their true values and exhibited substantially lower variability compared to those obtained from the ten alternative methods or direct estimation from incomplete data (in Fig. 4e, f). Importantly, msBayesImpute was the only method that consistently outperformed protein-wise mean imputation in terms of recovering the true median abundance across both dilution experiments.

### Reliable detection of differentially expressed proteins with msBayesImpute

A central task of MS-based proteomics in biological and biomedical studies is to identify proteins whose abundances differ significantly between conditions or patient groups. However, MNAR missing values can hinder this process by reducing statistical power and introducing estimation biases, for example, by shifting group-wise means. We therefore evaluated whether msBayesImpute improves differential expression (DE) analysis compared to other MVI methods using a lung cancer serial dilution dataset (tumor versus tumor-free). DE analysis was performed using limma^27^ after imputation, except for proDA^24^, and DPC^21,22^, which directly model missingness and operate on unimputed data.

We first assessed the impact of two preprocessing strategies—impute-first and normalize-first—on DE results. Specifically, we compared t-statistics derived from imputed data with those obtained from the ground-truth data. As shown in Supplementary Fig. 3c, d, the impute-first strategy was generally preferred for msBayesImpute and MsImpute, as it yielded t-statistics more consistent with the ground truth. In contrast, the normalize-first strategy was favored for PIMMS, MOFA2, BPCA, KNN, missForest, QRILC, MinDet, and protein-wise mean imputation.

**Fig. 5:**
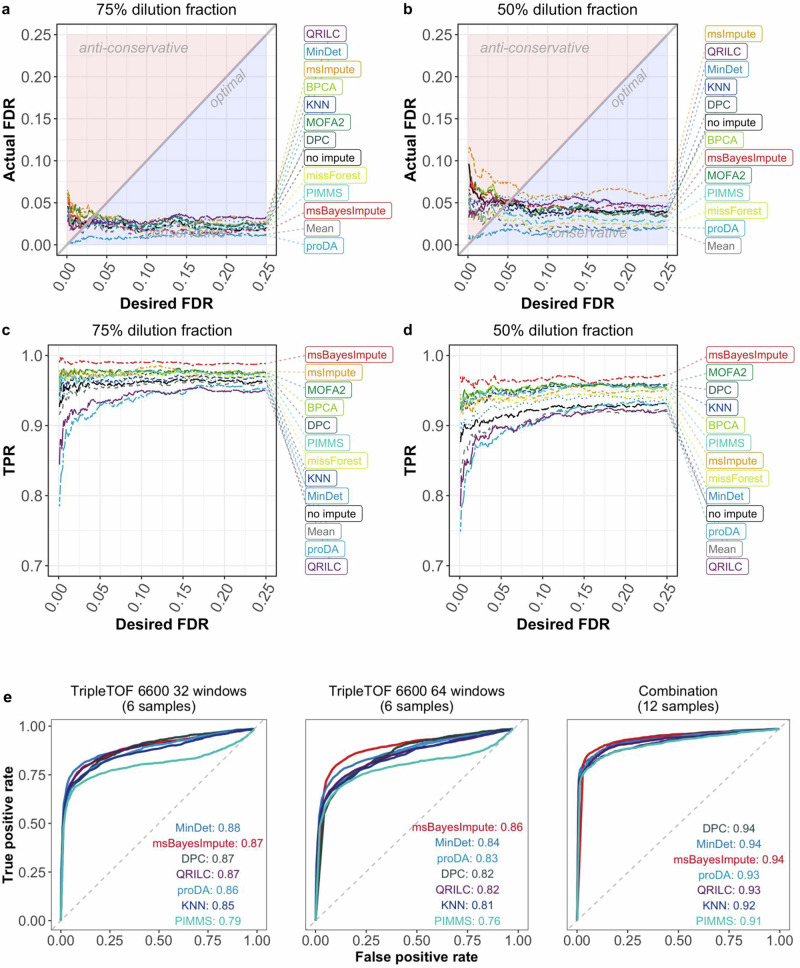
Performance comparison of differential expression analysis. **a**, **b** Relationship between desired and actual false discovery rate (FDR) in the 75 and 50% dilution datasets. Note that, due to the lack of a truly differentially expressed protein list, the desired FDR corresponds to the cutoff used to define differentially expressed proteins in the undiluted dataset (ground truth), while the actual FDR reflects the proportion of false discoveries when applying the same cutoff to imputed datasets. Therefore, the “ground truth” protein list will also grow when the desired FDR increases, which leads to a flatter look of the curves. A gray diagonal line indicates the optimal value. Light blue color indicates a conservative FDR, whereas orange indicates an anti-conservative FDR. **c**, **d** True positive rate (TPR) in the 75 and 50% dilution datasets, calculated as the proportion of ground truth positives (from the undiluted dataset) recovered in the imputed datasets. **e** Receiver operating characteristic (ROC) curves using mixed-species data across two runs (32 windows and 64 windows) with 6 samples each and one combined dataset of two runs with 12 samples. The AUROC values were used to quantitatively assess msBayesImpute alongside the other top six methods based on overall performance across three mixed-species datasets.

We next benchmarked the performance of different MVI methods in DE analysis in terms of false discovery rate (FDR) and true positive rate (TPR). Because the true set of biologically differentially expressed proteins between tumor and tumor-free tissue samples is unknown, we treated the DE proteins identified in the undiluted dataset at varying FDR thresholds as the ground truth. We then assessed the observed FDR and TPR for each method relative to this reference. In the 75% dilution dataset, where fewer values were missing, all methods controlled the FDR at both 5% (Fig. 5a). In the 50% dilution dataset, only MsImpute, QRILC, and MinDet failed to control the FDR at 5% while achieving control at 10% (Fig. 5b). Importantly, msBayesImpute achieved the highest TPR of all methods (Fig. 5c, d). Performance was further summarized using the F1 score at a target FDR of 5% (Table 1). msBayesImpute achieved the highest F1 scores in both dilution datasets (0.983 and 0.956), followed by DPC (0.975 and 0.953) and missForest (0.973 and 0.954). Protein-wise mean imputation yielded lower scores (0.970 and 0.934), while PIMMS, MOFA, BPCA, and KNN showed intermediate performance. MsImpute, MinDet, proDA, and QRILC performed comparably to unimputed data, indicating limited ability to simultaneously control FDR and maintain sensitivity, resulting in the poorest overall performance.

**Table 1:** F1 scores measurements from the serial dilution experiments of the lung cancer proteomes

Method	75% dilution fraction	50% dilution fraction
msBayesImpute	**0.983**	**0.956**
DPC	0.975	0.953
missForest	0.973	0.954
PIMMS	0.972	0.954
MOFA2	0.972	0.951
BPCA	0.972	0.949
KNN	0.969	0.951
Mean	0.970	0.934
msImpute	0.970	0.931
No impute	0.969	0.931
MinDet	0.967	0.932
proDA	0.961	0.933
QRILC	0.952	0.922

The F1 score, reflecting the balance between true positives and true negatives, was calculated with the desired false discovery rate (FDR) fixed at 0.05. Twelve imputation methods, along with no imputation, were evaluated.

To further validate these findings, we analyzed an independent real-world dataset comprising human samples spiked with yeast and *Escherichia coli* proteins. The dataset included six samples measured in two acquisition schemes (32-window and 64-window), each with balanced replicates (*n* = 3 per group) and distinct mixing ratios of yeast and *E. coli* proteins (Supplementary Data 3). This design reflects typical small-cohort experiments (e.g., 3 vs. 3). Combining both acquisition schemes yielded a dataset of 12 samples. All datasets exhibited an inverse relationship between protein abundance and missingness (Supplementary Fig. 4a, b). Ground-truth DE proteins were defined as the spiked-in yeast and *E. coli* proteins, and performance was evaluated using the area under the ROC curve (AUROC). Across the three mixed-species datasets, msBayesImpute ranked among the top-performing methods, achieving the highest AUROC in the 64-window and combined datasets and the second-highest in the 32-window dataset (Fig. 5e). MinDet showed comparable performance overall, while QRILC, DPC, proDA, and KNN achieved similar AUROC values but exhibited reduced performance in the 64-window dataset. In contrast, PIMMS consistently showed the lowest AUROC across all datasets.

Together, these results demonstrate that msBayesImpute most effectively balances sensitivity and specificity, enabling reliable detection of differentially expressed proteins and recovery of robust biological signals.

### Application of msBayesImpute to predict cell cycle stages using single-cell proteomics

**Fig. 6:**
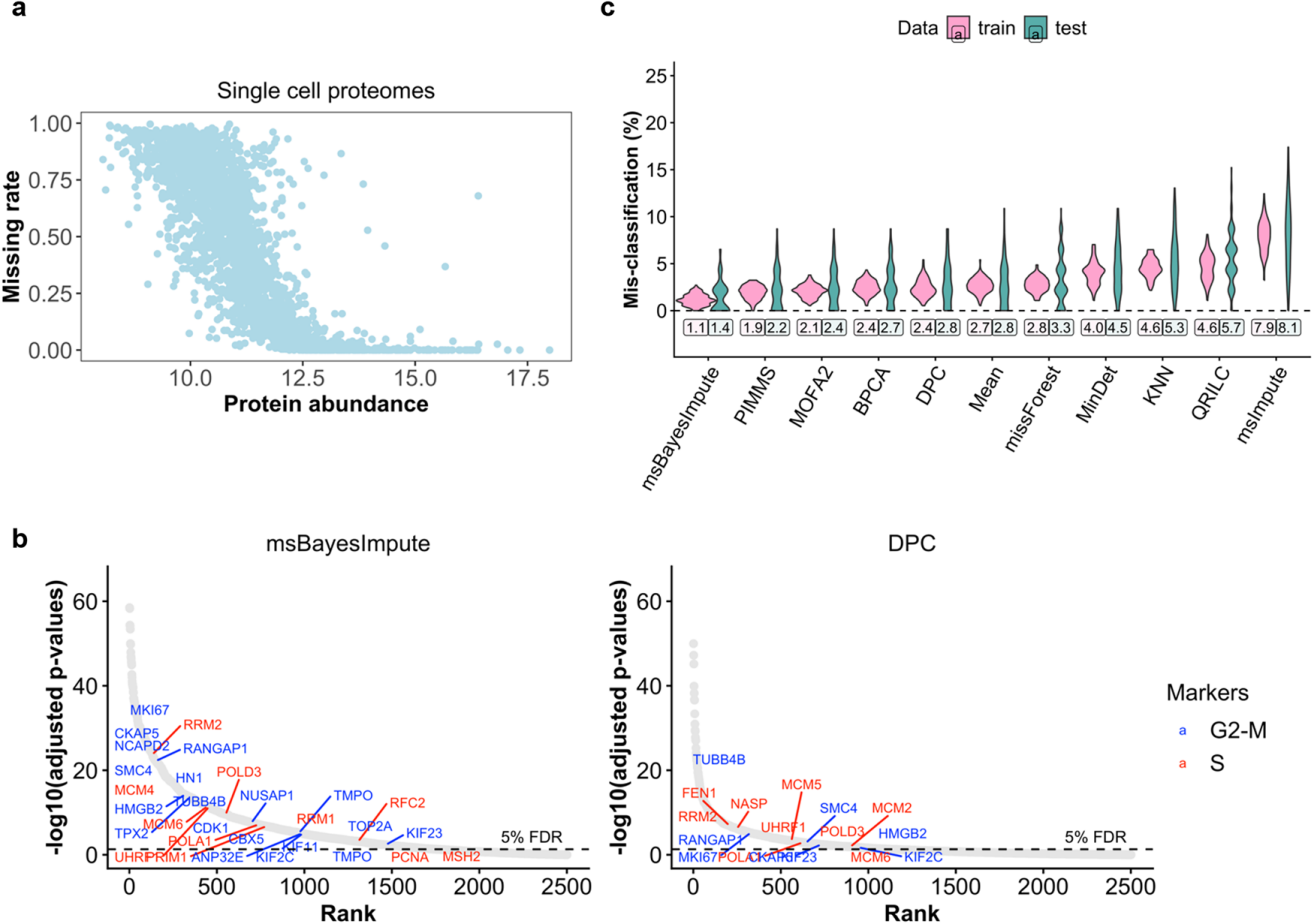
Benchmarking msBayesImpute using single-cell proteomics data across cell cycle stages. **a** Inverse relationship between protein-specific missing rate and average protein abundance. **b** Ranked negative log10 adjusted *p*-values for proteins identified by msBayesImpute and DPC, with G2–M phase markers highlighted in blue and S phase markers in red. *P*-values were calculated using two-sided moderated *t*-tests implemented in the limma *R* package, followed by Benjamini–Hochberg correction for multiple testing. **c** Misclassification error of eleven MVI methods on training and test sets, evaluated using a multivariate linear model with Lasso regularization and cross-validation for cell cycle stage prediction.

We next applied msBayesImpute to a publicly available DIA-based single-cell proteomics dataset comprising four cell cycle stages (G1, G1–S, G2, and G2–M), acquired on a timsTOF instrument and preprocessed with DIA-NN (Supplementary Data 4). This dataset was previously used by ref. ^22^ to evaluate the DPC model for differential expression (DE) analysis, using G2–M and S phase markers defined in the Seurat package^28^. The dataset exhibited high variability of total protein abundance per cell and high overall missing rate (45%), with missingness decreasing as protein abundance increased (Fig. 6a and Supplementary Fig. 5a, b). This pattern poses a significant challenge for downstream analyses if missing values are not properly handled.

msBayesImpute detected 18 of 20 G2–M phase markers at 5% FDR, whereas DPC detected only 8. For S phase markers, msBayesImpute and DPC achieved comparable performance, identifying 11 and 9 of 18 markers, respectively (Fig. 6b and Supplementary Fig. 5c–f).

To further assess performance in a downstream machine learning task, we trained a multivariate linear model with Lasso regularization to predict cell cycle stages using imputed protein abundance matrices from all MVI methods. A repeated cross-validation scheme (100 repetitions; see “Methods”) was used to ensure robust evaluation, and the distribution of misclassification errors was recorded. As shown in Fig. 6c, msBayesImpute achieved the lowest misclassification error on both training and test sets, outperforming all other methods. It was followed by PIMMS, MOFA2, and BPCA, while DPC exhibited approximately double the misclassification error of msBayesImpute. Moreover, msBayesImpute showed the lowest variability across test sets, whereas MsImpute displayed both the highest error rates and the greatest variability.

We also evaluated the sensitivity of factorization-based methods (msBayesImpute, MOFA, and BPCA) to the number of latent factors. msBayesImpute was the least affected by this parameter, followed by MOFA, whereas BPCA showed a strong dependence on the number of factors (Supplementary Fig. 5g).

The runtime of msBayesImpute for the single-cell dataset (*n* = 231) was approximately 1–2 min, consistent with that from the benchmarks on synthetic data (Supplementary Fig. 1b).

## Discussion

MS-based proteomics is now indispensable for advancing our knowledge of biological processes and their dysregulation in disease, yet missing values remain a persistent challenge that can compromise downstream analyses. Robust and accurate imputation is therefore critical. Here, we present msBayesImpute, a Bayesian matrix factorization-based method with a probabilistic dropout model that addresses both MAR and MNAR missingness in a data-driven way.

Our benchmarks across synthetic, semi-synthetic, experimental, mixed species, and single cell datasets spanning a wide range of sample sizes demonstrate that msBayesImpute offers clear advantages over existing MVI tools. It achieves higher imputation accuracy, particularly for low-abundance proteins that are most affected by MNAR dropout. Unlike many conventional methods, it also improves the recovery of sample-wise intensities (e.g., median abundance), enabling more accurate normalization and avoiding systematic biases that can propagate into downstream analyses. We therefore recommend applying imputation before normalization when using msBayesImpute. However, this advantage is modest, and the method remains effective when applied to already normalized data. Moreover, msBayesImpute consistently enhanced the detection of differentially expressed proteins, showing both stringent FDR control and superior sensitivity compared with other MVI methods and inherent DE modelling approaches such as proDA and DPC. Notably, even in single-cell proteomics—where missingness is more prevalent and often driven by MNAR mechanisms—msBayesImpute effectively reconstructs missing values, improves biomarker recovery, and supports accurate cell stage prediction using machine learning models.

Beyond its strong performance, msBayesImpute is characterized by flexibility and ease of use. It learns both dropout curves and number of latent factors directly from the data, without requiring predefined experimental designs. This distinguishes it from methods like proDA^24^, which are limited to specific designs (e.g., two-group comparisons). As a result, msBayesImpute is broadly applicable across different label-free proteomic platforms, including MaxQuant, DIA-NN, and DIA-Umpire, and support diverse applications from exploratory data analysis to machine learning workflows and signaling network modeling. It performs robustly across both small and large datasets, avoids the substantial sample size requirements typical of deep learning approaches such as PIMMS^23^, and is considerably more computationally efficient than missForest^15^. To facilitate adoption, we provide implementations in R and Python, along with an interactive Shiny application for users without programming experience.

Despite these strengths, msBayesImpute has certain limitations. Its reliance on a variational Bayesian framework means that uncertainty quantification is generally underestimated^29^, which could increase FDR in differential expression analysis and limit its integration into downstream statistical inference or modelling. Nevertheless, in our benchmarking of differential expression analysis, msBayesImpute controlled the FDR as well as, if not better than, alternative methods, suggesting that it is well suited for exploratory analyses and hypothesis generation. For applications where accurate uncertainty quantification is critical, bootstrap resampling with different random seeds—although computationally intensive—can provide more reliable uncertainty estimates. Another limitation of msBayesImpute is its reduced stability and imputation accuracy when the sample size is extremely small, e.g., <6 (Fig. 2a–c). In such cases, robust factorization and reliable estimation of protein-specific dropout curves become difficult in a purely data-driven manner. A potential solution would be to develop a pre-trained empirical model based on the growing number of large-scale proteomics datasets available in public repositories. Such a framework could provide informative priors to guide imputation in very small studies, thereby extending the applicability of msBayesImpute. Finally, although msBayesImpute is substantially more efficient than computationally heavy methods such as missForest, it remains slower than simpler approaches (e.g., MinDet, QRILC) and other factorization-based methods (e.g., PIMMS, MOFA) (Fig. 2k and Supplementary Figs. 2a, b and 3a, b). The main computational bottleneck is the inference of protein-specific dropout curves, which could be addressed through the use of pre-trained empirical models, more efficient computational engineering, and implementation in faster backends (e.g., C++ libraries instead of Python).

Several promising directions remain for extending and applying msBayesImpute. First, its probabilistic dropout model can be adapted to other MS-based data types, including phosphoproteomics, metabolomics, and lipidomics, broadening its utility. Second, integrating msBayesImpute with MOFA represents an exciting technical opportunity. While MOFA effectively models MAR missingness in multi-omics data, it does not explicitly account for MNAR patterns. Because msBayesImpute and MOFA share similar Bayesian factorization frameworks, it should be technically feasible to combine them, allowing users to optionally enable dropout modeling for MS-based datasets and thereby improve integration performance. Finally, incorporating more flexible dropout models, such as zero-inflated negative binomial functions, could extend its applicability to transcriptomics and beyond.

In conclusion, msBayesImpute provides a robust, versatile, and user-friendly framework for handling missing values in MS-based proteomics. By improving imputation accuracy, normalization, and the detection of biological signals, it contributes to more reliable and reproducible proteomics research and opens avenues for application across diverse omics domains.

## Methods

### MsBayesImpute architecture

The msBayesImpute model integrates Bayesian matrix factorization with a probabilistic dropout mechanism to impute missing values in numeric matrices representing protein abundances across samples measured by MS. Starting from MS-based data ${{\bf{X}}}\in {R}^{D\times N}$ with missing values, where *N* is the number of samples and *D* is the number of features (proteins) in the data matrix, msBayesImpute decomposes the original data matrix into lower-dimensional latent factor and weight matrices,

1 $${{\rm{X}}}={{\rm{W}}}{{\rm{Z}}}^{{\rm{T}}}+{\bar{{\rm{X}}}}+{\bar{{\rm{U}}}}^{{\rm{T}}}+{{\rm{E}}}$$

Where ${{\bf{W}}}\in {R}^{D\times K}$ denotes the weight matrix that maps latent factors to the observed feature space, and ${{\bf{Z}}}\in {R}^{N\times K}$ denotes the factor matrix that contains the low-dimensional latent representations of the samples. $\bar{{{\bf{X}}}}\,\in {R}^{D\times N}$ is a matrix with identical columns, representing the mean value (intercept) per feature. Due to the presence of missing values, the mean or median abundance of all features in a sample, which is important for sample normalization, could not be accurately calculated by only taking the observed values. Therefore, $\bar{{{\bf{U}}}}\,\in {R}^{N\times D}$ is introduced as a matrix of identical rows to model the true difference of mean abundance across samples. The residual noise matrix is represented by ${{\bf{E}}}$. ${{\bf{W}}}$, ${{\bf{Z}}}$, $\bar{{{\bf{X}}}}$, and $\bar{{{\bf{U}}}}$ are the parameters to be estimated with the variational Bayesian approach. After the inference of ${{\bf{W}}}$, ${{\bf{Z}}}$, $\bar{{{\bf{X}}}}$, and $\bar{{{\bf{U}}}}$ the complete data matrix without missing values can be reconstructed with Eq. (1). If the missing data points are MAR, they can be simply ignored during the inference process, which is how BPCA or MOFA performs MVI. However, in mass-spectrometry data, the missing values were usually not generated at random. Proteins with lower abundance tend to contain more missing data points. This trend can be described using a probabilistic model introduced by ref. ^24^, revealing that the likelihood of missing value decreases as protein abundance increases. The probabilistic dropout model $\bar{\varPhi }(\bullet )$ is mathematically represented by the cumulative distribution function of a Gaussian distribution $\varPhi (\bullet )$ characterized by feature-specific inflection $\rho$ and scale $\zeta$, and can be incorporated into the likelihood of a Bayesian factorization model:

2 $$\bar{\varPhi }\left({x|}\rho ,{\zeta }^{2}\right)=1-\varPhi \left({x|}\rho ,{\zeta }^{2}\right)=1-\frac{1}{\sqrt{2\pi \zeta }}{\int }_{-\infty }^{x}\exp \left(-\frac{{\left(t-\rho \right)}^{2}}{2{\zeta }^{2}}\right){dt}$$

### Likelihood models

The Bayesian matrix factorization framework of msBayesImpute combines two likelihood models: a Gaussian distribution for modelling observed values and the probabilistic dropout distribution (Eq. (2)) for modelling missing patterns. The observed entries in **X** follow Gaussian distributions ${{\mathscr{N}}}(\bullet )$ while other entries are ignored:

3 $${x}_{{dn}}\sim {{\mathscr{N}}}\left({\mu }_{{dn}},1/{\tau }_{d}\right){{\rm{If}}}\,{x}_{{dn}}{{\rm{is\; observed}}}$$

4 $${\mu }_{{dn}}={\sum }_{k\,=\,1}^{K}{w}_{{dk}}\cdot {z}_{{nk}}+{\bar{x}}_{d}+{\bar{u}}_{n}$$

Where $k\in \left\{1,\ldots ,K\right\}$ are latent factors, ${\mu }_{{dn}}$ corresponds to the reconstructed values using a list of factorization parameters, including ${w}_{{dk}}$, ${z}_{{nk}}$, ${\bar{x}}_{d}$ and ${\bar{u}}_{n}$, and ${\tau }_{d}$ stands for precision per feature.

On the other hand, the probabilistic dropout distribution, $\bar{\varPhi }\left(\bullet \right)$, is modelled as the cumulative density function of a Gaussian distribution, forming a sigmoid-shaped curve characterized by two key parameters, i.e., the inflection point, ${\rho }_{d}$, and the scale, ${\zeta }_{d}$, that are learnt per protein. Specifically, we established feature-wise probabilistic dropout distributions to model the missing likelihood, accounting for the varying extent of missing patterns associated with each protein. Since both missing and observed entries in **X** carry missingness information, all entries are fitted using probabilistic dropout models:

5 $${x}_{{dn}}\sim \bar{\varPhi }\left({\rho }_{d},{\zeta }_{d}^{2}\right)$$

### Probabilistic dropout priors

We assume the missing information can be inferred from the given data ${{\bf{X}}}$, with the dropout patterns varying slightly across features. Therefore, we sample the inflection point per feature from a Normal distribution using the global inflection point ${\rho }_{0}$ derived from **X** as the location,

6 $${\rho }_{d}{{\mathscr{\sim }}}{{\mathscr{N}}}\left({\rho }_{0},\,1/{{\mathcal{m}}}\right)$$

7 $$m\sim {{\mathscr{G}}}\left({\mathrm{100,10}}\right)$$

Where ${{\mathscr{G}}}(\bullet )$ represents a gamma distribution. For the scale of each dropout mode, we assume they do not directly stem from the global scale of ${\zeta }_{0}$, but rather from hierarchical priors, reflecting the high heterogeneity in scales as compared to the inflections.

8 $${\zeta }_{d}\sim {{\mathscr{G}}}\left({n}_{d}\bullet {z}_{d},{z}_{d}\right)$$

9 $${n}_{d}\sim {{{\mathscr{N}}}}^{+}\left({\zeta }_{0},1\right)$$

10 $${z}_{d}\sim {{\mathscr{G}}}\left({\mathrm{200,10}}\right)$$

Where ${n}_{d}\in {R}^{+}$ indicates the constant negative relationship between MS-based abundance and missing rates to represent the missing value patterns observed in MS proteomics data. The global ${\rho }_{0}$ and ${\zeta }_{0}$ can be directly achieved from the given data ${{\bf{X}}}$,

11 $${p}_{d}={R}_{d}/N$$

12 $${{argmin}}_{{\rho }_{0},\,{\zeta }_{0}}{\sum }_{d=1}^{D}{\left({p}_{d}-\bar{\varPhi }\left({\bar{y}}_{d}|{\rho }_{0},{\zeta }_{0}\right)\right)}^{2}$$

Here, $d\in \left\{1,\ldots ,D\right\}$ are features, ${R}_{d}$ corresponds to the number of missing values per feature, and ${\bar{y}}_{d}$ denotes complete values per protein imputed by MinDet method, which serves as an initialization step prior to training (see parameter inference for details).

Moreover, the hyperpriors for the rate of inflection and the rate of scale, i.e., $m$ and ${z}_{d}$, can influence performance as protein-specific missing patterns are assumed to be heterogeneous. We benchmarked these hyperpriors using synthetic data through a grid search. The combinations of (100, 10) for the gamma distribution of the rate $m$, and (200, 10) for the rate ${z}_{d}$, indicated in Eqs. (7) and (10) led to the best and most stable performance.

### Matrix factorization priors

The parameters in Eqs. (4) and (5) are inferred from a list of priors. The precision ${\tau }_{d}$ of the Gaussian likelihood can be modelled using the following Gamma and Gaussian hyperpriors,

13 $${\tau }_{d}\sim {{\mathscr{G}}}({{a}_{d}}\cdot {{b}_{d}},{{b}_{d}})$$

14 $${b}_{d}\sim {{\mathscr{G}}}\left({\mathrm{1,1}}\right)$$

15 $${a}_{d}{{\mathscr{\sim }}}{{\mathscr{N}}}\left(c,1\right)$$

16 $$c\sim {{\mathscr{G}}}\left(1{e}^{-14},1{e}^{-14}\right)$$

Where the hyperparameter for ${b}_{d}$ was determined by a grid search. An uninformative prior was initially used for the hyperparameter $c$ governing the protein-specific precision ${\tau }_{d}$, following a strategy similar to MOFA^17^. However, in settings with small sample sizes and high levels of missingness, such uninformative priors can lead to convergence issues. To address this, msBayesImpute estimates an initial global residual and uses it as an informative prior to replace $c$ in Eq. (16). Specifically, msBayesImpute first performs PCA on data imputed using MinDet. It then derives a noise matrix by removing components that explain more than 5% of the variance. Sample-specific variances are subsequently computed from this noise matrix and averaged to obtain a global residual, which serves as an informative prior for $\tau$.

Among ${w}_{{dk}}$, ${z}_{{nk}}$, ${\bar{x}}_{d}$ and ${\bar{u}}_{n}$, Gaussian priors were assigned to ${z}_{{nk}}$, ${\bar{x}}_{d}$ and ${\bar{u}}_{n}$, while a shrinkage prior was employed for ${w}_{{dk}}$ to reduce spurious correlations and false positives (FP). We provided the Horseshoe prior^30^ for feature-wise sparsity represented by ${{\eta }}_{k}$ and ${\lambda }_{{dk}}$. ${{\eta }}_{k}$ are factor-specific variables used to estimate the general level of activeness for each factor, while ${\lambda }_{{dk}}$ is a feature-specific shrinkage parameter allowing each element in **W** to escape the factor-specific sparsity.:

17 $${\bar{x}}_{d}\sim {{\mathscr{N}}}\left({e}_{d},1\right)$$

18 $${\bar{u}}_{n}\sim {{\mathscr{N}}}\left(0,{v}_{n}\right)$$

19 $${z}_{{nk}}\sim {{\mathscr{N}}}\left({\mathrm{0,1}}\right)$$

20 $${w}_{{dk}}\sim {{\mathscr{N}}}\left(0,{{\eta }}_{k}\cdot {\lambda }_{{dk}}\right)$$

Here, ${e}_{d}$ stands for empirical mean and ${v}_{n}$ is empirical variance, both computed per feature. ${\lambda }_{{dk}}$ was sampled from a positive Half–Cauchy distribution $C\left(\cdot \right)$, and ${{\eta }}_{k}$ was sampled from a Beta distribution. To automatically learn ${{\eta }}_{k}$, and ${\lambda }_{{dk}}$, we used uninformative fixed priors introduced in ref. ^31^,

21 $${\lambda }_{{dk}}\sim {{{\mathscr{C}}}}^{+}\left({\mathrm{0,1}}\right)$$

22 $${{\eta }}_{k}\sim {Beta}\left({\mathrm{0.5,0.5}}\right)$$

Finally, the joint probability density function is generated by these prior and likelihood distributions:

23 $$p= 	 {{\prod }_{{d}=1}^{D}{\prod }_{{n}=1}^{N}{{\mathscr{N}}}\left({x}_{{dn}}|{\mu }_{{dn}},1/{\tau }_{d}\right)}^{\left\{{{{\rm{x}}}}_{{{\rm{dn}}}}{\mbox{observed}}\right\}}\\ 	 \times {\prod }_{d=1}^{D}{\prod }_{n=1}^{N}\bar{\varPhi }\left({x}_{{dn}}|{\rho }_{d},{\zeta }_{d}^{2}\right)\\ 	 \times {\prod }_{n=1}^{N}{\prod }_{k=1}^{K}{{\mathscr{N}}}\left({z}_{{nk}}|{\mathrm{0,1}}\right)\\ 	 \times {\prod }_{d=1}^{D}{\prod }_{k=1}^{K}{{\mathscr{N}}}\left({w}_{{dk}}|0,{{\eta }}_{k}\cdot {{{\rm{\lambda }}}}_{{dk}}\right)\\ 	 \times {\prod }_{d=1}^{D}{{\mathscr{N}}}\left({\bar{x}}_{d}|{E}_{d}\left[X\right],1\right)\\ 	 \times {\prod }_{n=1}^{N}{{\mathscr{N}}}\left({\bar{u}}_{n}|0,{Va}{r}_{n}\left[X\right]\right)\\ 	 \times {\prod }_{k=1}^{K}{{{\mathscr{C}}}}^{+}\left({\lambda }_{{dk}}|0,\,1\right)\\ 	 \times {\prod }_{d=1}^{D}{{\mathscr{G}}}\left({\tau }_{d}|{a}_{d}\cdot {b}_{d},{b}_{d}\right)\\ 	 \times {\prod }_{d=1}^{D}{{\mathscr{G}}}\left({b}_{d}|{\mathrm{1,1}}\right)\\ 	 \times {\prod }_{d=1}^{D}{{\mathscr{N}}}\left({a}_{d}|c,1\right)\\ 	 \times {\prod }_{d=1}^{D}{{\mathscr{G}}}\left({\zeta }_{d}|{n}_{d}\cdot {z}_{d},{z}_{d}\right)\\ 	 \times {\prod }_{d=1}^{D}{{\mathscr{N}}}\left({n}_{d}|{\zeta }_{0},1\right)\\ 	 \times {\prod }_{d=1}^{D}{{\mathscr{G}}}\left({z}_{d}|{\mathrm{200,10}}\right)\\ 	 \times {\prod }_{d=1}^{D}{{\mathscr{N}}}\left({\rho }_{d}|{\rho }_{0},1/m\right)\\ 	 \times {{\mathscr{G}}}\left(c|1{e}^{-14},1{e}^{-14}\right)\\ 	 \times {{\mathscr{G}}}\left(m|{\mathrm{100,10}}\right)\\ 	 \times {Beta}\left({{\eta }}_{k}|{\mathrm{0.5,0.5}}\right)$$

### Data simulation

To evaluate whether the msBayesImpute model could capture the ground truth parameters, we first simulated data from the generative model msBayesImpute was based on with varying numbers of features and factors. For each simulated dataset without missing values, a weight matrix and a factor matrix with specific dimensions were drawn from a standard Gaussian distribution. After the multiplication of weight and factor matrices, intercepts for each feature, sampled from Gaussian distributions parameterized by specified means and variances, were added. To mimic the variability observed in real proteomics, Gaussian noise was also introduced, with precision parameters per feature drawn randomly from a Gamma distribution.

To introduce missing values that resemble the real-world missing pattern in label-free MS proteomics, two methods were used. One method generates missing values with the probabilistic dropout function on which msBayesImpute is based, where feature-wise inflection and scale parameters are drawn from a Gaussian distribution with a chosen location and standard deviation. This method is useful for validating our model, but may introduce a favourable bias towards our model when benchmarked against other MVI models. On the other hand, this kind of probabilistic dropout likelihood is currently only suitable for label-free methods rather than others like TMT- or SILAC-labelled MS data. Therefore, another method, which was introduced by ref. ^8^, generates missing values based on mixing two missing type mechanisms, i.e., MAR and MNAR, with a manual specific of mixture proportions. The MAR mechanism creates missing values randomly in the quantitative dataset. For the MNAR mechanism, a threshold matrix with the same dimension of the original dataset is sampled from a Gaussian distribution, centred at a chosen quantile of the original data and with a standard deviation set to 0.3 times that of the original distribution. Values in the original dataset are designated as missing if they fall below their corresponding thresholds. A key strength of this method is its precise control over the missing rate associated with each type of missingness. It also avoids bias during benchmarking.

To examine the effect of missing rates and sample sizes on prediction by msBayesImpute, datasets were systematically generated with missingness rates ranging from 10 to 90% in 10% increments and sample sizes from 6, 12, 20, 100, 200, 500, to 1000, using the probabilistic dropout function, as missing rate and sample sizes are critical factors influencing the performance of imputation methods^13^. Next, we generated 50 samples with 10, 30, 50, 70, and 90% to evaluate the impact of missingness across different imputation methods. For assessing normalization accuracy, the variance of sample-wise intercepts was further artificially increased and added to the data.

### Hyperparameter tuning

A grid search was performed over three fixed hyperpriors: the rate parameter $m$ for the inflection parameter ${\rho }_{d}$, the rate $z$ for the scale parameter ${\zeta }_{d}$, and the rate $b$ for the precision parameter ${\tau }_{d}$. Candidate values ranged from (1e−14, 1e−14), representing diffuse (“flat”) priors, to (2000, 100), corresponding to more concentrated (“narrow”) priors. Five simulated datasets (20 samples, 5000 features) with known ground-truth parameters were generated following the procedure described in the *Data Simulation* section. Performance was evaluated based on the ability to recover the true parameters ($\rho$, $\zeta$, $\tau$) and overall imputation accuracy, quantified using RMSD between inferred and ground-truth values. Results for all parameter combinations are shown in Supplementary Fig. 6. Some combinations demonstrated improved recovery of $\zeta$ and $\tau$ (left panel; green lines), particularly when $z$ ≠ (1, 1) and $b$ = (1, 1). Among these, we further prioritized combinations that yielded stable performance across simulations, as indicated by lower variability in RMSD (right panel; red lines). From these candidates, we selected (100, 10) for $m$, (200, 10) for $z$, and (1, 1) for $b$ (highlighted in blue), as this combination achieved the lowest imputation error while maintaining stable performance. Although the grid search is not exhaustive and cannot cover all possible real-world scenarios with varying sample and feature sizes, our results suggest that the choice of hyperpriors does not substantially affect performance as long as they fall within reasonable ranges.

### Parameter inference

The msBayesImpute is a matrix factorization-based model using a probabilistic Bayesian framework and SVI. The main idea is to approximate the intractable posteriors by maximizing the evidence lower bound. The msBayesImpute employs Adam, a stochastic gradient descent optimizer with a learning rate of 0.05 and also employs a Normal distribution with a diagonal covariance matrix as an automatic guide to approximate the posterior distribution. The convergence includes two modes, slow and fast, with training terminating when the supervised loss difference remains below a specified threshold associated with the convergence mode for three consecutive iterations. A random seed is used to control stochastic processes and ensure reproducibility across model runs. The msBayesImpute model consists of two modules, the initialization block and the refinement block. The maximum number of training iterations per block is 30,000.

An overview of the msBayesImpute algorithm and parameter inference procedure is depicted in Supplementary Fig. 7. The process begins by estimating global parameters$\,\rho$_0_ and $\zeta$_0_ given data matrix **X** using values initially imputed with MinDet, as defined in Eqs. (11) and (12). This is followed by an initial training step in which dropout and matrix factorization parameters are optimized simultaneously. During this initialization phase, the model can automatically determine the number of latent factors by iteratively removing factors that explain less than a user-defined minimum proportion of variance (default: 1%). Alternatively, users may specify the number of latent factors a priori; in this case, the variance threshold is ignored and the initialization is performed in a single iteration. At least one latent factor is always retained.

To further refine the model and improve imputation accuracy, msBayesImpute performs three additional refinement iterations in which dropout and factorization parameters are optimized separately. Specifically, factorization parameters are updated in the first and third iterations, while dropout parameters are updated in the second iteration. When estimating one set of parameters, the other is held fixed. The same convergence criterion used during initialization is applied in each iteration, with a maximum of 10,000 epochs per optimization step.

The parameter inference procedure is implemented in Python using the probabilistic programming framework Pyro, with an accompanying R wrapper provided for accessibility.

### Comparator methods and parameter settings

The MVI methods benchmarked against msBayesImpute included PIMMS, missForest, MOFA, BPCA, MsImpute, KNN, MinDet, QRILC, DPC, and proDA, with feature-wise mean imputation and no-imputation used as baselines. To reflect realistic biomedical applications where ground truth is unknown, parameter settings were not tuned across datasets, thereby avoiding data leakage and enabling unbiased performance evaluation.

PIMMS was only available in Python, whereas BPCA and missForest had both R and Python implementations. The Python implementation of missForest was excluded due to substantially lower computational efficiency compared to the *R* version. All other methods were executed in *R*.

Default or recommended parameters were used for all methods unless otherwise required. For PIMMS^23^ (pimmslearn package), the DAE model was selected due to its higher reconstruction accuracy. MOFA (MOFA2, v1.20.2) was run with default settings, including automatic relevance determination and a 1% variance-explained threshold. BPCA^16^ (pcaMethods v2.2.0; Python implementation by Diedrich L.: https://github.com/lucas-diedrich/bpca.git) was applied with centering enabled, and the number of factors was fixed to match that used in MOFA and msBayesImpute.

KNN, MinDet, and QRILC were implemented via MsCoreUtils (v1.22.1). For KNN, the number of neighbours was set to the default value (*k* = 10), and the maximum allowed missingness per feature was adjusted (80–100%) to accommodate different datasets. For MinDet and QRILC, default parameters were used (quantile *q* = 0.01; tune.sigma = 1), as MNAR distributions cannot be reliably specified a priori.

missForest (v1.6.1) was applied with default parameters (ntree and maxiter unchanged) to balance accuracy and runtime; a minimum of two observed values per feature was required. MsImpute (v1.20.0) required at least four observed values per feature and was run with default MAR/MNAR weights (0.8/0.2 or vice versa depending on design), as the true proportion is unknown.

proDA was applied with default moderation parameters, including moderate_location and moderate_variance. DPC (limpa v1.2.5) was used in automatic mode (dpcCN) to estimate dropout parameters, followed by dpcQuant for imputation or dpcDE for differential expression analysis.

### Generation of the semi-synthetic benchmarking dataset

The semi-synthetic benchmarking dataset was derived from a label-free MS-based proteomics dataset of HeLa cells^32^, processed with MaxQuant 1.6.12 (ProteomeXchange ID: PXD042233), to which artificial missing values were introduced. The dataset comprises two protein expression matrices with 564 and 50 samples, respectively, and was originally used to benchmark the PIMMS method, enabling evaluation across different sample sizes. The missing value generation mechanism that mixes MAR and MNAR with a fixed proportion by ref. ^8^, which has been used for benchmarking PIMMS^23^ and by others in similar benchmarking studies^11,13,26^, was used to introduce missing values in the semi-synthetic data for a fair comparison among different MVI methods. In addition, this missing generation approach can control MNAR rates, which can be employed to assess the robustness of imputation methods when the composition of the missing pattern changes.

### Generation of the benchmarking dataset with serial dilution experiment

To better recapture the missing value pattern in a real-world situation, the missing values were experimentally introduced through mass-spectrometry measurements of lysates of 20 paired tumour/tumour-free tissues from 10 lung cancer patients without dilution and with dilution fractions of 75 and 50%.

Human lung tissue samples were obtained from patients with lung adenocarcinoma and provided by the Lung Biobank Heidelberg, a member of the accredited Tissue Bank of the National Center for Tumor Diseases Heidelberg. The study was approved by the Ethics Committee of the Medical Faculty of the University of Heidelberg (approval number: S-270/2001 (biobank vote) and S-154/2018 (study vote). Written informed consent was obtained from all patients. Experienced pathologists oversaw tumor dissection, selecting tumor-enriched regions as well as corresponding tumor-free areas. Samples were received snap-frozen and were kept on dry ice during processing. For sample preparation, two slices each were transferred to separate tubes containing a small spoon of protein extraction beads (Protein Extraction Beads, diagenode #C20000021) for tissue lysis. Tissue was stored at −80 °C. After thawing on ice, between 50 μL to 150 μL of 1× RIPA lysis buffer with 1% NP40 and 0.1% SDS was added (1% NP40, 0.1% SDS, 1% sodium deoxycholate, 25 mM Tris-HCl pH 7.6, 150 mM NaCl, 10 mM NaF, 1 mM Na3VO4, 250 U/mL benzonase, 10 U/mL DNase, 0.1% AEBSF, 0.1% aprotinin), depending on the size of tissue slices. To ensure a full coverage with lysis buffer, samples were spun down 1 min at 14,000 rpm at 4 °C prior to incubation at 60 °C for 2 h. Subsequently, samples were centrifuged for 1 min at 14,000 rpm at 4 °C. Samples were sonicated in a Bioruptor Pico (Diagenode) for 10 cycles (30 s on /30 s off, easy mode, 4 °C). After an additional centrifugation step (14,000 rpm, 10 min, 4 °C), the supernatant was collected, and the concentration of tissue lysates were determined by using the BCA Assay Kit (Thermo Scientific Pierce # A55864).

Protein reduction, alkylation, cleanup, and tryptic digestion were performed by following the SP3 protocol on an automated liquid handling platform (Agilent Bravo). 55 μg protein was used for the further procedure and was prepared in a 96-well plate (Superplate, Thermo #AB-2800) in a total volume of 30 μL of 100 mM triethylammonium bicarbonate (TEAB, Sigma Aldrich #T7408-100ml). Prior to running the protocol on an Agilent Bravo, 40 mM tris-(2-carboxyethyl)-phosphine (TCEP, Sigma Aldrich # C4706-2G) and 160 mM 2-chloroacetamide (CAA, Sigma Aldrich # C0267-100G) were prepared as stock solutions for the reduction of disulfide bonds and alkylation. For sample cleanup, master mixes of Sera-Mag Speed Beads A (hydrophobic coating) and B (hydrophilic coating) (GE Healthcare, Beads A #65152105050250, Beads B # 45152105050250) were prepared in a ratio of 1:1 by using 100 μL of each bead suspension. To remove the acidified solution in which the beads are stored in, the bead master mix was cleaned by placing the tubes on a magnetic rack until all beads were located to the magnet (approx. 1 min) and the solution appeared clear. Supernatant was removed, beads were resuspended in 200 μL MS-compatible H_2_O and put back to the magnetic rack. This procedure was performed in total three times. Beads of each master mix were resuspended in a volume of 90 μL to achieve a final volume of 100 μL. To enhance protein binding to the beads surface, 100% MS-compatible ethanol was prepared as well as 80% MS-compatible ethanol for the protein cleanup procedure. For protein digestion buffer a solution containing 2.2 μg Trypsin Gold (PROMEGA # V5280)/75 μL 100 mM TEAB was prepared (1:25 protease to protein ratio). After preparation of the deck overlay on the Agilent Bravo, the following steps were executed. Reduction and alkylation were performed by adding 15 μL of TCEP (final conc. 10 mM) and CAA (final conc. 40 mM), followed by an incubation step at 95 °C for 5 min. Protein cleanup was initiated by the addition of 5 μL beads master mix and 65 μL 100% MS-compatible ethanol to a final concentration of 50% (v/v). The sample plate was shaking for 15 min at 800 rpm to allow sufficient protein binding. After incubation, the sample plate was moved to a magnetic rack, supernatant was removed after 1 min and beads were washed by resuspending in 80% MS-compatible ethanol. Wash steps were executed three times. As a final step 75 μL of digestion buffer was added to the beads and were incubated 16 h at 37 °C on a heating block of Agilent Bravo. To avoid sample evaporation, the plate was sealed with sealing foil as soon as tryptic digestion started. After incubation, the sample plate was centrifuged for 1 min at 14,000 rpm. Sealing foil was removed and digested proteins were transferred to a new plate using the magnet and 96LT pipetting head on Agilent Bravo deck. After lysate collection peptides were dried down by vacuum centrifugation at 45 °C. Samples were reconstituted in 130 μL MS-compatible 80% ACN + 0.1% FA. Afterwards, 5 μg (equal to 12.3 μL) were transferred to a new 96-well plate and dried down by vacuum centrifugation at 45 °C for full proteome measurement and dilution series. The remaining 50 μg were used for a different procedure. Finally, the sample plate was stored at −20 °C until reconstitution for MS measurement. For reconstitution and dilution, loading buffer (0.1% formic acid (FA), 2% ACN in MS-compatible H_2_O) was used. Digested, undiluted peptide samples (100%) were dissolved in 15 μL loading buffer. Out of this initial reconstitution, a 75% dilution was prepared, followed by the 50% dilution based on the previous generated dilution (75%).

Nanoflow LC-MS/MS was performed by coupling an UltiMate 3000 to an Orbitrap Eclipse Tribrid mass spectrometer (Thermo Scientific). Reconstituted and diluted samples were spiked in by using an injection volume of 2 µL. Peptides were loaded to a trapping column (PEPMAP NEO C18 5 µm 0.3 × 5 mm, 1500 bar) at a flow rate of 30 µL/min in 100% buffer A (0.1% FA in MS-compatible H2O). After loading onto an analytical column (100 μm ID × 15 cm length, packed in-house with Reprosil-Pur 120 C18-AQ, 1.9 μm resin, Dr. Maisch), peptides were separated using a 85 min gradient from 2 to 38% of buffer B (0.1% FA, 80% ACN in MS-compatible H2O) at a 300 nL/min flow rate. The Orbitrap Eclipse™ Tribrid™ mass spectrometer was operated in data-independent mode (DIA), automatically switching between full scan MS and MS2. Full scan MS spectra were acquired in the Orbitrap at 120,000 resolution, m/z ranging from 350 to 1400, after accumulation to the set target value of 300% (100% = 1.2e6) with a maximum injection time of 45 ms. The DIA method covers 30 isolation windows with a dynamic range of 400–1000 m/z and uses a higher energy collisional dissociation (HCD) for fragmentation at normalized collision energy (N)CE of 28%. MS2 spectra were acquired in the Orbitrap at a resolution of 30,000 with a target value of 1000% (100% = 5e5) charges after accumulation for a maximum of 54 ms. Generated data was measured in positive ion mode.

Protein identification and quantification were performed using DIA-NN (v1.9) in library-free mode with deep learning–based spectral prediction. An in silico spectral library was generated directly from FASTA sequences of reviewed proteins from Uniprot, using tryptic digestion (cleavage at K/R, up to two missed cleavages), allowing peptides of 7–30 amino acids, precursor m/z 300–1800, fragment m/z 200–1800, and precursor charge states of 1–4. Carbamidomethylation of cysteine was set as a fixed modification, while methionine oxidation (UniMod:35) was included as a variable modification (maximum one variable modification per peptide), with peptidoform-level scoring enabled. DIA-NN was configured to optimise mass accuracy and scan window settings independently for each run. Protein inference was performed using heuristic protein grouping. FDR was controlled at 1% at precursor and protein levels. Quantification was based on precursor-level intensities without additional normalisation or match between runs to ensure independence of search results of each dilution fraction, and protein group level quantification matrices across samples were exported for downstream analysis in *R*. The values of data points that were observed in the undiluted samples, while unobserved in the diluted samples, were used as the ground truth values for benchmarking.

### Mixed species proteomics

The dataset generated by ref. ^33^ (ProteomeXchange ID: PXD002952) was used as an additional real-world benchmarking dataset alongside the lung cancer serial dilution dataset. In this study, hybrid proteome samples were created by spiking yeast and *E. coli* lysates into a constant human protein background. Samples were analyzed using SWATH-MS, a data-independent acquisition (DIA) method for label-free quantification, at two different mixing ratios, each with three technical replicates.

Raw MS data acquired on a TripleTOF 6600 instrument were processed using the DIA-Umpire platform under two acquisition schemes: 32 fixed windows and 64 variable windows. To increase sample size for benchmarking, we additionally combined both datasets, resulting in a total of 12 samples.

The expected log₂ fold changes between the two groups were defined as 0 for human proteins, −1 for yeast proteins, and 2 for *E. coli* proteins (Supplementary Fig. 4a).

### Single-cell proteomics across cell cycle stages in HeLa cells

The single-cell MS-based proteomics dataset was generated from HeLa cells across four cell cycle stages, induced by drug treatment^34^ (ProteomeXchange ID: PXD024043). Data were acquired on a timsTOF instrument and processed using DIA-NN (v1.8). The dataset comprises 231 single cells, including 93, 41, 52, and 45 cells from the G1, G1–S, G2, and G2–M phases, respectively, with an overall missing rate of approximately 45% (Supplementary Fig. 5a, b).

### Data pre-processing

A standardized set of preprocessing steps was applied to datasets for all imputation methods to ensure comparability. Proteins that were missing in all samples after introducing missing values were first removed. Certain imputation methods, including MissForest and MsImpute, require a minimum of 2 and 4 observations per protein, respectively. For specific comparisons, such as with PIMMS, we followed the same processing steps outlined in their published papers to ensure comparable and reliable results, like removal of proteins with a missing rate greater than 75% and samples with a missing rate greater than 50%. For the semi-synthetic, experimental, mixed species and single cell data, a log transformation of base 2 was applied to the spectra intensities prior to imputation.

Two normalization strategies were used depending on the analysis task: median normalization for reconstruction accuracy and variance stabilizing normalization (VSN) for differential expression and machine learning analyses. For fair error estimation, outputs from impute-first methods were normalized in the same way as the ground-truth data. Prior to downstream analyses, imputed values from impute-first methods were transformed back to the raw abundance scale to enable application of VSN, which has been shown to outperform commonly used approaches such as median normalization, in differential expression analysis^35^.

### Performance evaluation

For benchmarking the accuracy of imputation, Root Mean Squared Deviation (RMSD) was used to compare the ground truth values and imputed values:

24 $${RMSD}=\sqrt{\frac{{\sum \left({x}_{i}-{y}_{i}\right)}^{2}}{N}}$$

Where $x$ and $y$ stand for ground true and imputed values, respectively. Lower RMSD reflects greater imputation accuracy. All MVI methods were performed under different random seeds for 5 repetitions to obtain 95% confidence intervals and to assess the stability of each method.

Moreover, applying normalization before imputation may introduce bias, making it essential to assess how well different imputation methods handle subsequent normalization compared to the original datasets with missing values as a baseline dataset using synthetic datasets. As an additional baseline, we also considered a prefiltered dataset in which proteins with more than 50% missing values were removed, since 50% filtering is a common analytical strategy. In this study, we assessed performance by comparing sample-wise median measurements from the imputed datasets to those from the baseline datasets. To assess normalization accuracy using experimental data, we calculated the differences in sample-wise intercepts between imputed and ground-truth values and centered these differences to evaluate their dispersion around zero.

To perform differential expression on ground truth and imputed expression matrices, linear models with Empirical Bayes moderated t-statistics implemented in the limma package (*R* version 3.60.6)^27^ was used, except for ProDA and DPC, which performs differential expression analysis directly on an unimputed matrix. The measurements, including the TPR, FDR, false positive rate, F1 score, and AUROC, are used to evaluate the accuracy of a statistical test.

In the undiluted expression data, proteins with adjusted *p*-values less than 0.05 were considered as actual positives, and those identified from the imputed matrices were treated as predicted positives. Predicted positives that overlapped with actual positives were classified as true positives and those that did not were considered FP, while proteins in actual positives rather than in predicted positives were designated as false negatives. Therefore,

25 $${FDR}=\frac{{FP}}{{Predicted\; Positives}}=\frac{{FP}}{{TP}+{FP}}$$

26 $${TPR}=\frac{{TP}}{{Actual\; Positives}}=\frac{{TP}}{{TP}+{FN}}$$

27 $$F1 {score}=\frac{2}{(1-{FD}{R})^{-1}+{TP}{R}^{-1}}=\frac{2{TP}}{2{TP}+{FP}+{FN}}$$

The F1 score, which balances precision and recall, was used as an overall measure of imputation performance, with higher values indicating better accuracy.

For the mixed-species dataset, where the ground truth set of differentially expressed proteins is known, the area under the receiver operating characteristic curve (AUROC) was used to assess discriminative performance independent of threshold selection:

28 $${FPR}=\frac{{FP}}{{Actual\; Negatives}}=\frac{{FP}}{{TN}+{FP}}$$

29 $${AUC}=\int {TPR}({FPR}){dFPR}$$

The AUROC is used to summarize the trade-off between sensitivity and specificity, with a value of 0.5 indicating random classification and a value of 1 representing perfect classification performance.

For cell cycle prediction, a multivariate linear model with Lasso regularization was trained using the glmnet package (R version 4.1−10)^36,37^. Model performance was evaluated using ten-fold cross-validation and was repeated 100 times for each MVI method. In each run, the data were randomly split into a training set (80% of samples) and a test set (20%). Performance was assessed by computing the mean cross-validated misclassification error on the training set and the prediction error on the test set using the best-performing model.

## Reporting summary

Further information on research design is available in the Nature Portfolio Reporting Summary linked to this article

## Supplementary information

Transparent Peer Review file

Supplementary Information

Description of Additional Supplementary Files

Supplementary Data 1

Supplementary Data 2

Supplementary Data 3

Supplementary Data 4

Machine learning reporting summary

## Data Availability

The public HeLa cell line datasets used in this study (564 and 50 samples) are available at (10.5281/zenodo.10854544), with the corresponding raw data deposited in PRIDE under the identifier PXD042233. The mixed-species and single-cell proteomics datasets for cell cycle analysis are also available in PRIDE under the identifiers PXD002952 and PXD024043, respectively. The raw proteomics dataset of paired tumor and tumor-free tissue samples from 10 lung cancer patients is available in PRIDE under the identifier PXD077257. Processed data are available at https://github.com/Lu-Group-UKHD/msBayesImpute_manuscript (10.5281/zenodo.20493062). The source data for Fig. 3 are provided in Supplementary Data 1, those for Figs. 4 and 5 are provided in Supplementary Data 2 and 3, and those for Fig. 6 are provided in Supplementary Data 4.

## Code availability

The *R* code for msBayesImpute package can be found at: https://github.com/Lu-Group-UKHD/msBayesImpute (10.5281/zenodo.20492993). The Python code can be found at https://github.com/Lu-Group-UKHD/msBayesImpute_Py (10.5281/zenodo.20492725). A workflowR project with the analysis code, figures, and results associated with the manuscript is available at https://github.com/Lu-Group-UKHD/msBayesImpute_manuscript (10.5281/zenodo.20493062). A Shiny app demo for msBayesImpute is available at: https://mozi.embl.de/public/msBayesImpute/.
