# Supplementary material for: msBayesImpute as a versatile framework for addressing missing values in biomedical mass spectrometry proteomics data
Source: Commun Chem. 2026 Jul 7;9:236. doi: 10.1038/s42004-026-02106-3 (PMC13342297; doi:10.1038/s42004-026-02106-3)
Supplement: Supplementary file 2 — Supplementary Information [file 42004_2026_2106_MOESM2_ESM.pdf]

Supplementary Information for "msBayesImpute as a Versatile Framework for Addressing Missing Values in Biomedical Mass Spectrometry Proteomics Data"

Authors

Jiaojiao He[1], Barbara Helm[2,3], Franziska Gödtel[2], Katharina Büchner[2,3], Marcel Schilling[2,3], Marc A. Schneider[3,4], Laura V. Klotz[3,5], Jana Braunger[6,7], Hauke Winter[3,5], Britta Velten[6,7], Ursula Klingmüller[2,3], Junyan Lu[1*]

Affiliations

[1]Medical Faculty Heidelberg, Heidelberg University.
[2]System Biology of Signal Transduction, German Cancer Research Center (DKFZ).
[3]Translational Lung Research Center Heidelberg (TLRC), Member of the German Center for Lung Research (DZL)
[4]Translational Research Unit, Thoraxklinik at Heidelberg University Hospital.
[5]Department of Thoracic Surgery, Thoraxklinik at Heidelberg University Hospital
[6]Biological Data Science, Center for Organismal Studies (COS), Heidelberg University
[7]Interdisciplinary Center for Scientific Computing (IWR), Heidelberg University

Supplementary Figures

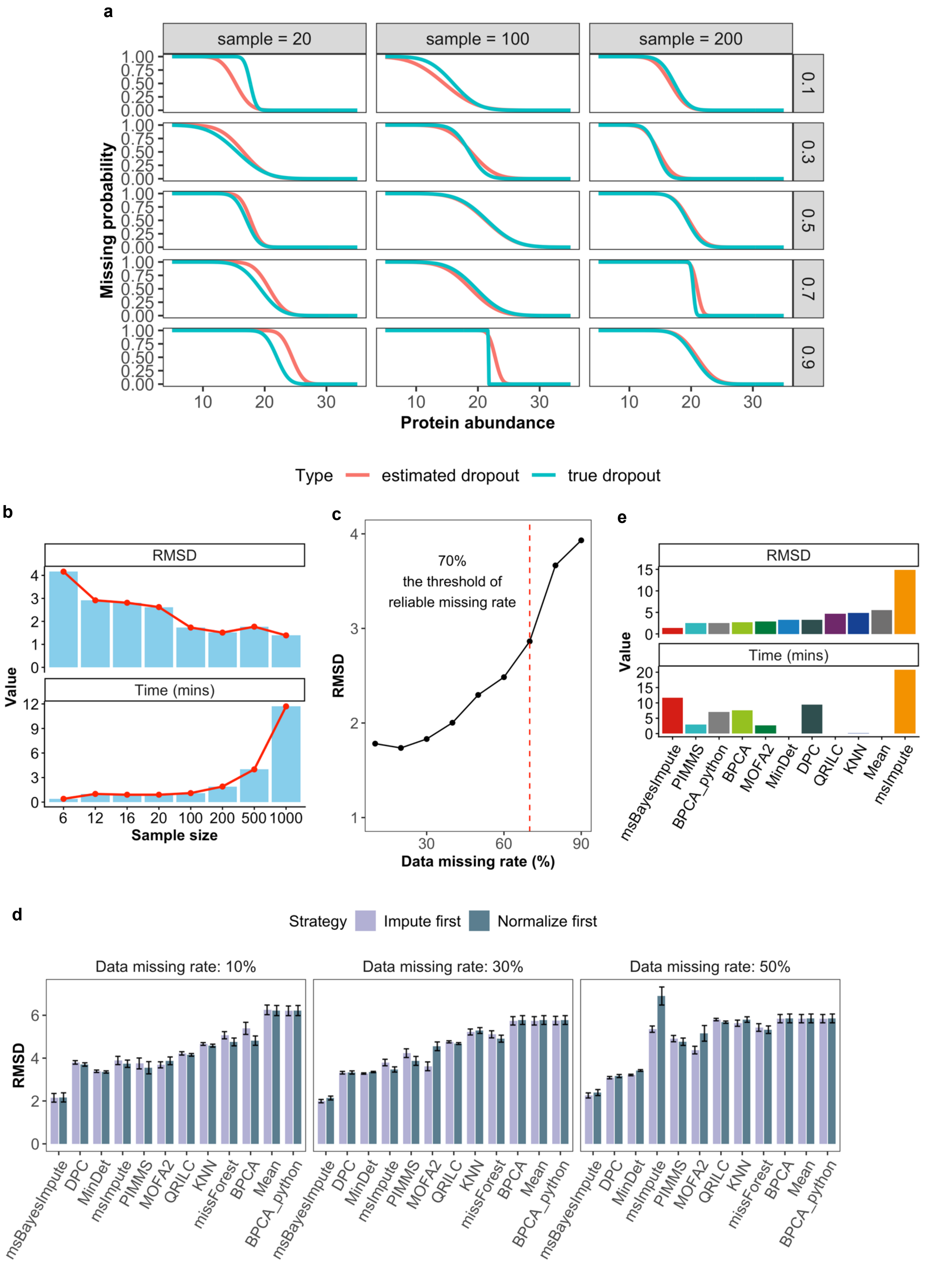

Supplementary Figure 1. Imputation accuracy evaluated using synthetic datasets. (a) Fifteen protein-specific dropout patterns randomly selected across different missing rates for datasets with sample sizes of 20, 100, and 200. **(b)** Root mean squared deviation (RMSD) for missing value reconstruction and computational time of msBayesImpute across synthetic datasets ranging from 6 to 1,000

samples. **(c)** RMSD for missing value reconstruction as a function of overall missingness using msBayesImpute. The sharp increase between 70% and 80% missingness is highlighted in red. **(d)** Comparison of impute-first and normalize-first strategies across twelve MVI methods under varying missing rates using synthetic datasets. **(e)** As in **(b)**, but including ten MVI methods (excluding missForest) to evaluate RMSD and computational time on synthetic datasets with 1,000 samples.

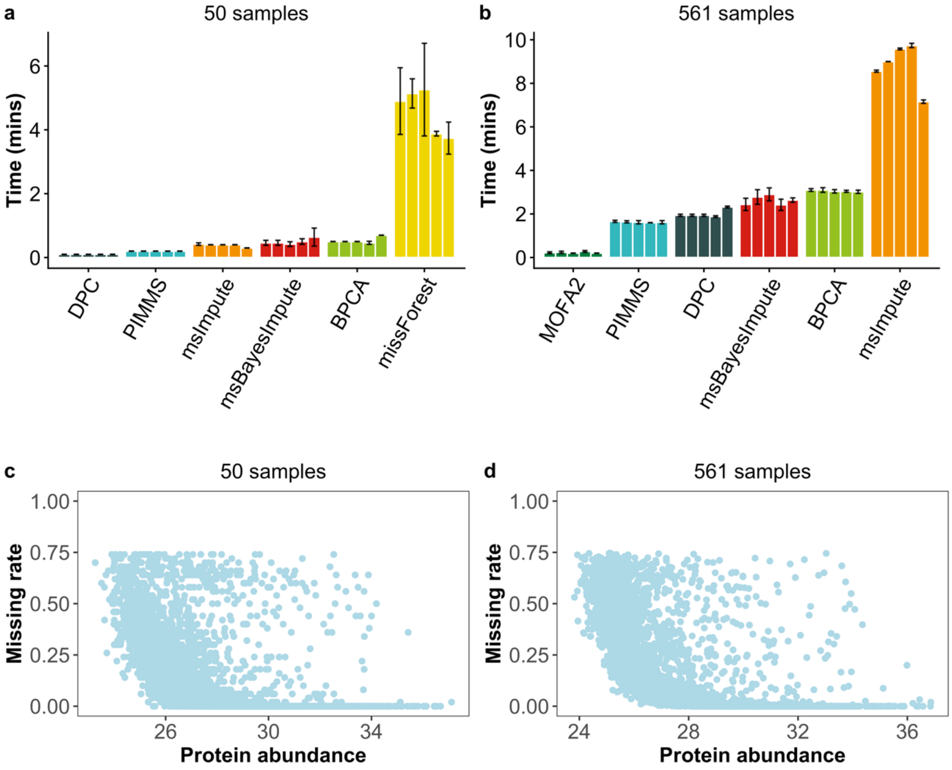

Supplementary Figure 2. Computational time and missingness patterns in semi-synthetic HeLa proteomics data. (a) Runtime comparison of computationally intensive MVI methods (PIMMS, msBayesImpute, DPC, MsImpute, missForest, and BPCA) on HeLa cell line datasets. In small-sample datasets, msBayesImpute completed within ~1 minute, whereas missForest required ~4.5 minutes. **(b)** Runtime comparison for large-sample datasets (excluding missForest). msBayesImpute completed in ~2 minutes and MsImpute in ~9 minutes, while simpler methods (protein-wise mean, KNN, MinDet, and QRILC) completed in <1 second. **(c, d)** Scatter plots showing the relationship between missing rate and average protein abundance across datasets with different sample sizes.

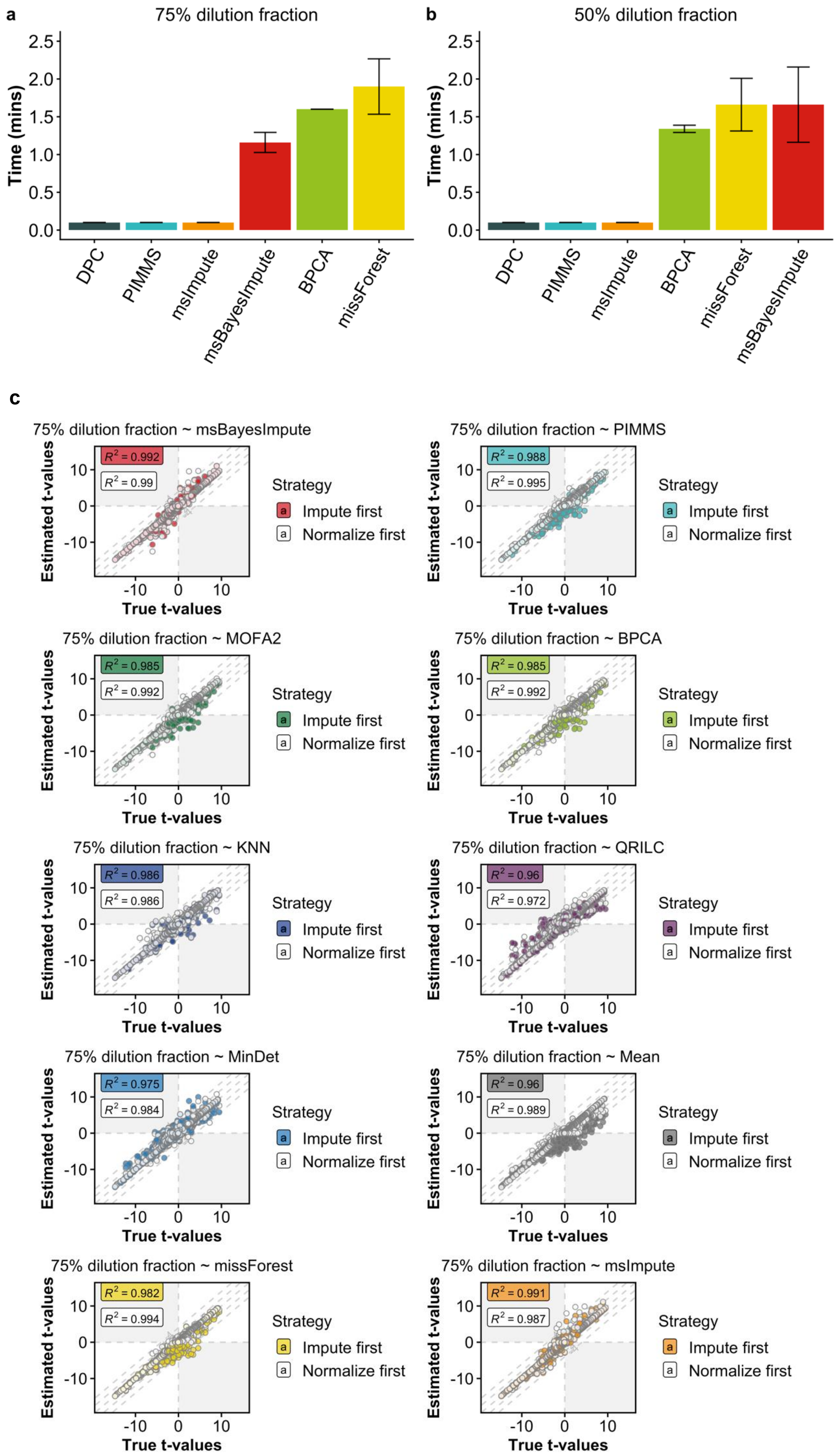

a
75% dilution fraction
Time (mins)
2.5
2.0
1.5
1.0
0.5
0.0
DPC
PIMMS
msImpute
msBayesImpute
BPCA
missForest
b
50% dilution fraction
Time (mins)
2.5
2.0
1.5
1.0
0.5
0.0
DPC
PIMMS
msImpute
BPCA
missForest
msBayesImpute
c
75% dilution fraction ~ msBayesImpute
R^2 = 0.992
R^2 = 0.99
75% dilution fraction ~ PIMMS
R^2 = 0.988
R^2 = 0.995
75% dilution fraction ~ MOFA2
R^2 = 0.985
R^2 = 0.992
75% dilution fraction ~ BPCA
R^2 = 0.985
R^2 = 0.992
75% dilution fraction ~ KNN
R^2 = 0.986
R^2 = 0.986
75% dilution fraction ~ QRILC
R^2 = 0.96
R^2 = 0.972
75% dilution fraction ~ MinDet
R^2 = 0.975
R^2 = 0.984
75% dilution fraction ~ Mean
R^2 = 0.96
R^2 = 0.989
75% dilution fraction ~ missForest
R^2 = 0.982
R^2 = 0.994
75% dilution fraction ~ msImpute
R^2 = 0.991
R^2 = 0.987
Estimated t-values
True t-values
10
0
-10
Strategy
Impute first
Normalize first

d

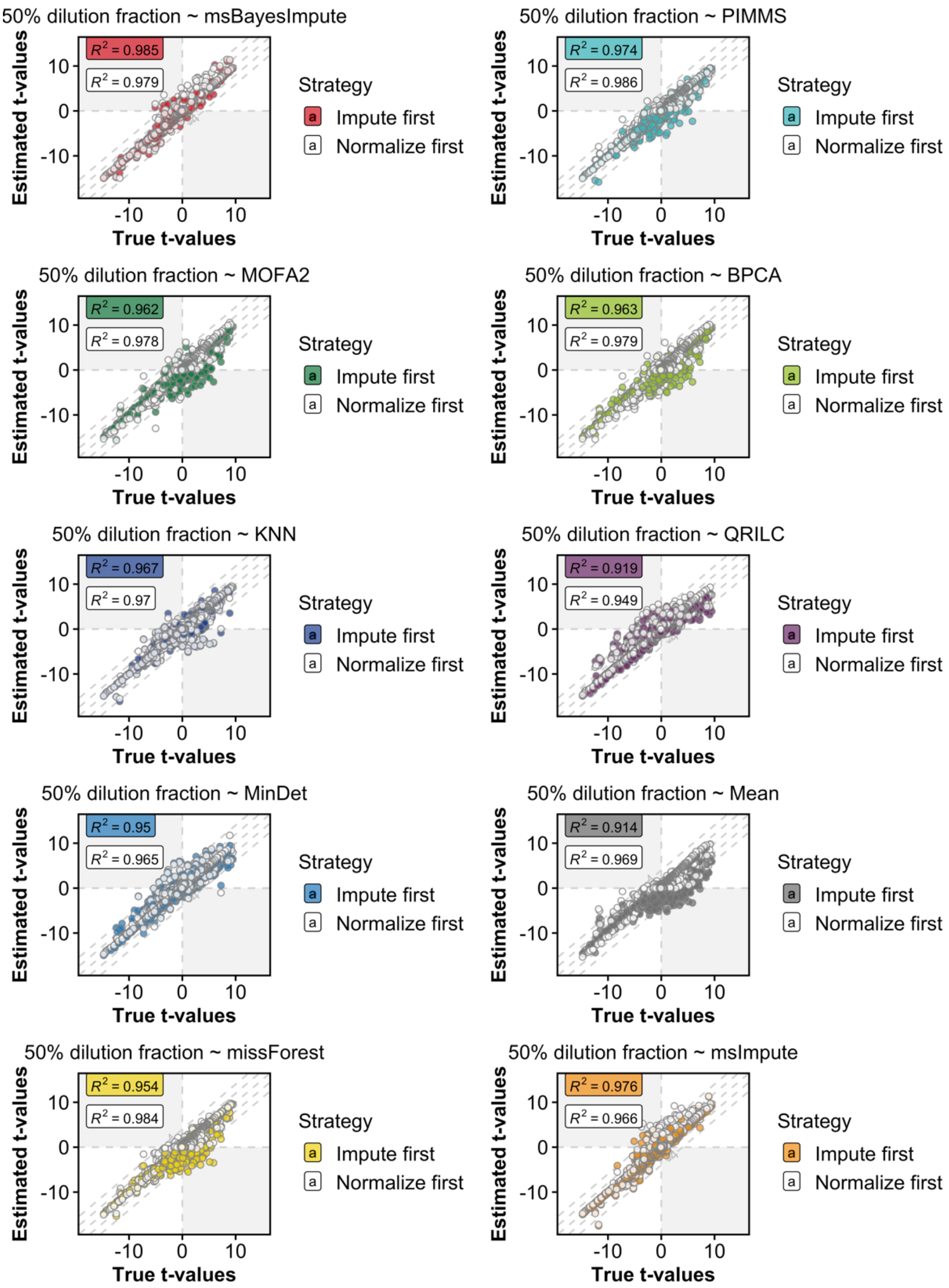

Supplementary Figure 3. Computational time and preprocessing strategy comparison using lung cancer dilution datasets. (a, b) Runtime of MVI methods on lung cancer serial dilution datasets. msBayesImpute required ~1 minute for the 75% dilution dataset and ~1.5 minutes for the 50% dilution dataset. BPCA and missForest showed comparable runtimes. **(c, d)** Scatter plots comparing true versus estimated t-statistics derived from limma under impute-first and normalize-first strategies across ten MVI methods. R^2 values indicate the agreement between estimated and ground-truth t-statistics for each strategy.

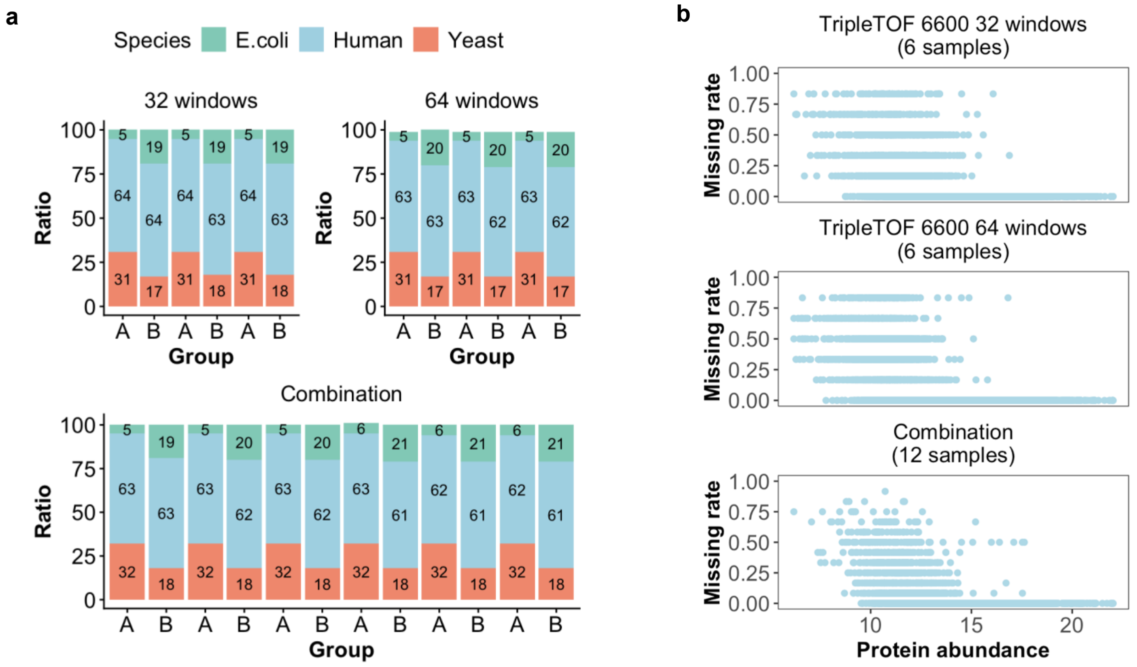

Supplementary Figure 4. Performance variability of msBayesImpute in mixed-species proteomics datasets. (a) Experimental design showing two mixing ratio groups under 32- and 64-window acquisition configurations, and combined dataset. **(b)** Scatter plots illustrating the relationship between missing rate and average protein abundance across the two configurations and the combined dataset.

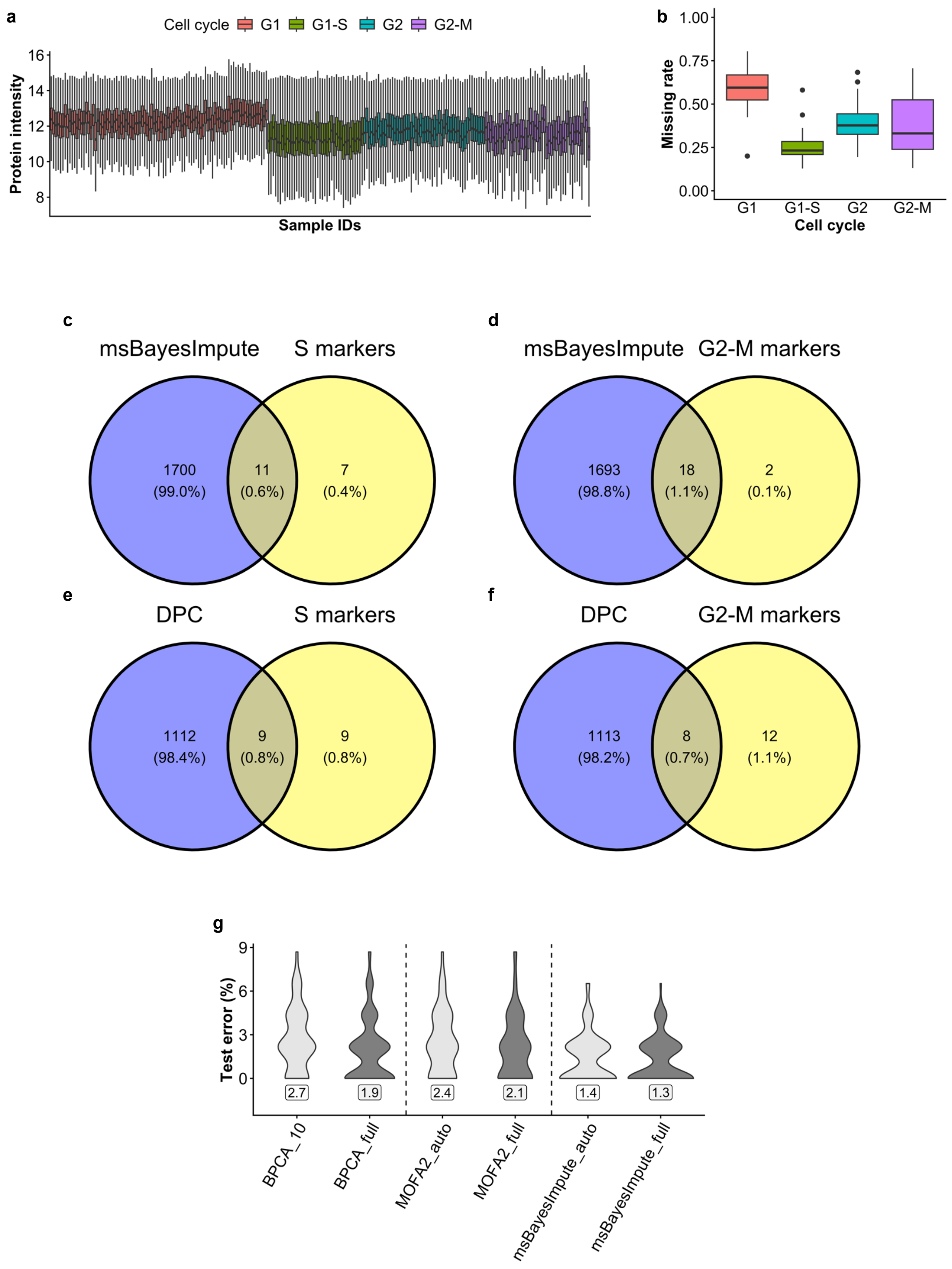

Supplementary Figure 5. Differential expression and machine learning analyses using single-cell proteomics data across cell cycle stages. (a) Distribution of protein abundance across samples, with cells colored by cell cycle stage. **(b) As in (a)**, showing the relationship between missing rate and cell cycle stage. **(c–f)** Venn diagrams comparing true S-phase and G2–M phase markers with those identified by msBayesImpute and DPC. **(g)** Misclassification error on test sets was evaluated using a multivariate linear model with Lasso regularization and cross-validation. Three factorization-based methods were assessed to examine the impact of the number of latent factors on predictive performance. For msBayesImpute, the number of factors can either be user-defined or automatically inferred. Models labeled "_full" indicate that the number of factors was fixed to the number of samples, whereas "_auto" denotes automatic factor selection. For BPCA, automatic factor selection is not supported; therefore, the number of factors was either fixed to the number of samples ("_full") or set to 10, corresponding to the number of factors inferred by msBayesImpute and MOFA2 under automatic tuning.

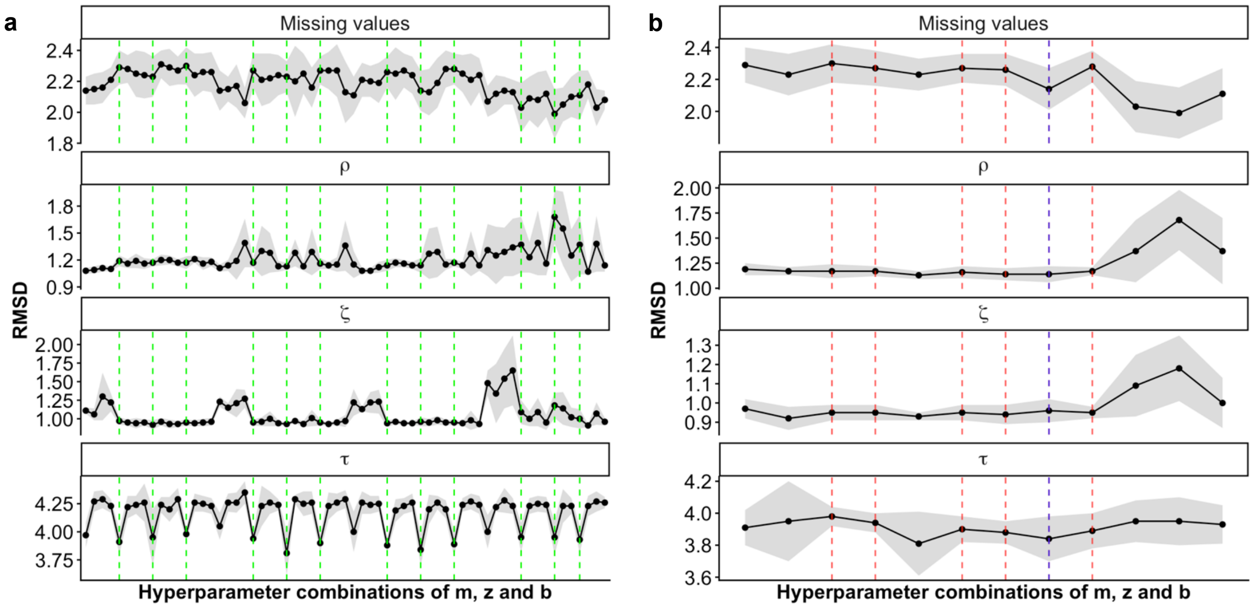

Supplementary Figure 6. Grid search for hyperparameters m, z, and b. (a) Root mean squared deviation (RMSD) between true and inferred parameter values across different combinations of the three hyperpriors. Green lines indicate combinations achieving relatively optimal performance for the rate parameters z (scale parameter ζ) and b (precision parameter τ). **(b)** Refinement of candidate combinations identified in **(a)**. Grey shading represents the standard deviation of RMSD across five simulated datasets. Red lines denote combinations with the lowest variability (i.e., most stable performance), and the blue line highlights the final selected combination, which yields the lowest imputation error among these candidates.

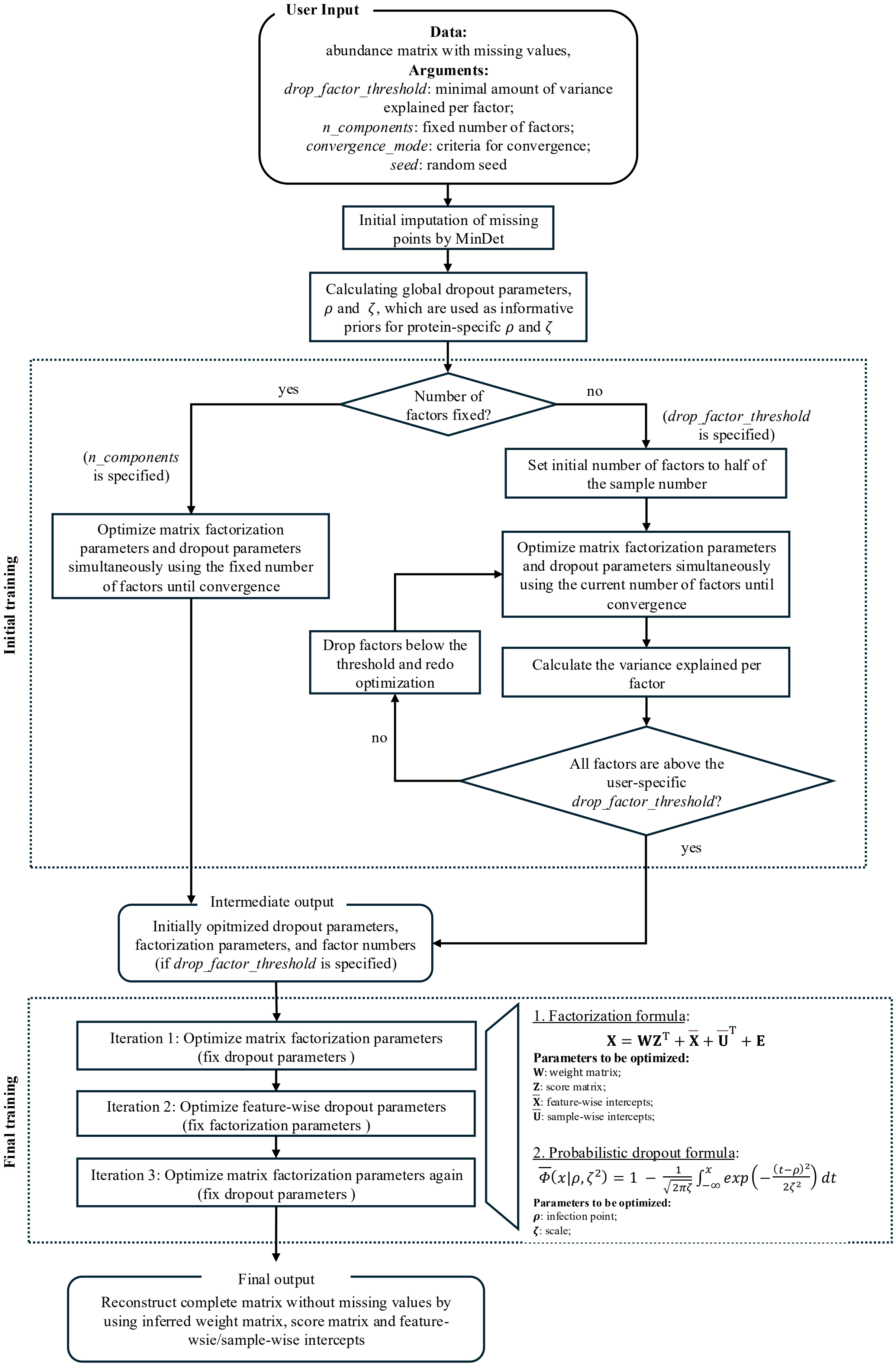

Supplementary Figure 7. Overview of the msBayesImpute algorithm.
