# Supplementary material for: msBayesImpute as a versatile framework for addressing missing values in biomedical mass spectrometry proteomics data
Source: Commun Chem. 2026 Jul 7;9:236. doi: 10.1038/s42004-026-02106-3 (PMC13342297; doi:10.1038/s42004-026-02106-3)
Supplement: Supplementary file 3 — Description of Additional Supplementary Files [file 42004_2026_2106_MOESM3_ESM.pdf]

Description of Additional Supplementary Files:

File: Supplementary Data 1

Description: The source data for Figure 3

File: Supplementary Data 2

Description: The source data for Figure 4-5

File: Supplementary Data 3

Description: The source data for Figure 4-5

File: Supplementary Data 4

Description: The source data for Figure 6
