# Supplementary material for: msBayesImpute as a versatile framework for addressing missing values in biomedical mass spectrometry proteomics data
Source: Commun Chem. 2026 Jul 7;9:236. doi: 10.1038/s42004-026-02106-3 (PMC13342297; doi:10.1038/s42004-026-02106-3)
Supplement: Supplementary file 1 — Transparent Peer Review file [file 42004_2026_2106_MOESM1_ESM.pdf]

nature portfolio

Peer Review File

msBayesImpute as a Versatile Framework for Addressing Missing Values in Biomedical Mass Spectrometry Proteomics Data

Corresponding Author: Dr Junyan Lu

This file contains all reviewer reports in order by version, followed by all author rebuttals in order by version.

Attachments originally included by the reviewers as part of their assessment can be found at the end of this file.

Version 0:

Reviewer comments:

Reviewer #1

(Remarks to the Author)
The authors present a novel approach to data imputation for mass spectrometry proteomics data. These data sets typically exhibit gaps due to MAR and MNAR cases, which hinders the application of various analysis techniques. Given synthetic and few actual "real-world" data sets, the authors demonstrate the capabilities of their Bayesian inspired approach in terms of accuracy and run time which both are reported to be comparable or superior compared to existing methods.
The manuscript is written all in all well. The topic is of relevance in the field, the statistical analyses are mostly carried out in a sound way.

Nevertheless, several points are not adequately addressed and require clarification before publication. While a detailed list of concerns is provided below, some major points are:
(1) It is not clear to what extent the synthetic data examples are sufficient to underpin the capabilities of the method in current, but particularly also in future data sets. The latter is essential, given the deluge of data we expect in the upcoming years in this sector.
(2) Some notations are mathematically sloppy and need to be corrected.
(3) The authors claim their method is parameter-free. Looking into its details, it seems to the reviewer that there are parameters, but the authors simply froze them to fixed values. Similarly, for the comparison with other methods, those methods were also fixed in terms of their parameter choices. While these choices might be chosen in a reasonable range, it renders the comparison only partially valid. This issue becomes further pronounced by choosing mostly R-based methods which are then compared to actual Python code (which is only wrapped so that it can be included in the R environment), which is not necessarily fair considering run time comparisons. This comparison requires at least a more solid discussion, or optimally a more detailed evaluation and discussion of parameter spaces and choices. Furthermore, some of the methods, that the comparison is carried out with, could also be used to evaluate gappy data or a minimum set of data extracted from the gappy set -- well aware, that this will not necessarily shed perfect insight into the biology, this can still yield valuable insights into errors the methods make by filling in gaps.

Further and more detailed comments:
- L61: "addressing missing values MS proteomics data is imputation,"
-> weird sentence structure, please re-phrase

- L145: "Each matrix contained 5,000 proteins and 10 latent factors, with a median protein abundance of 20 (see Methods). To assess the effect of sample size, we simulated datasets with 20, 100, and 200 samples. Missing values were then introduced at rates of 10%, 30%, and 50% using a probabilistic dropout function that assigned distinct missingness patterns to proteins."
-> in which regard is this choice covering the relevant range of typically missing value patterns? Higher missingness is not uncommon. How can it be guaranteed that the method still performs reasonably well under such and other extreme conditions, that are out of range of the provided parameters? Also, how well does it perform for bigger data sets in terms of accuracy but also run time?

- Fig 2f,g,h,i: what is the x-axis showing in detail?

- L219: We generated a series of data matrices with MNAR proportions of 0%, 25%, 50%, 75%, and 100% for both the smaller (N=50) and larger (N=561) datasets after filtering out proteins with greater than 75% missing values and samples with greater than 50% missing values.“
-> what does 100% mean here? Not clear! Further it would be interesting, how the method performs on the entire data set, including the “>75%” proteins and “>50%” samples cases!

- Fig3: What are the run times in these practical applications? Are they comparable to the ones mentioned for the artificial data set?

- L412: “X [bar] is a vector…”
-> this cannot be true, same for U [bar]. Both can only be matrices of shape N x D, which might have identical rows/ columns, respectively. The authors should be concise in the notation. Also, dimensions of W and Z are not provided in the section.

- L458: “The combinations of (100, 10) for the gamma distribution of the variance m and (200, 10) for the rate z, indicated in equations (7) and (10) led to the best and most stable performance.“
-> how has this been deduced? The authors should provide proof for this and an explanation how this is justified for arbitrary data sets

- L463-464: Eq. 13-16: In L114-115, it is argued that the presented method does not require parameter tuning. Considering Eq 13-16 and following modeling assumptions, various variables have been chosen by the authors fixed, but are actually tunable. The authors should re-phrase their statement in L114-115 and motivate the parameter choices, as well as potential variations thereof.

- L493: “Additionally, if variances differ significantly across features, but the sample size is too small for the model to capture this variation, an additional initialization step is applied.”
-> which additional initialization step? What is being done in this case?

- L498-504: this is a very high level description of an iterative procedure. The authors should provide a sketch of their algorithm, incorporating the most essential mathematical steps and this iterative procedure in the manuscript for the sake of self-containedness.

- L511-512: “All methods, except for PIMMS, which was only available in Python, were accessible in R.“
-> this should also be stated when run times are compared. R is sometimes rather slow, a comparison with mostly R-based code sounds like an apple-and-oranges comparison.

- L522-531: here the parametrization of the other approaches that msBayesImpute is compared with are laid out. How sensitive are the reported data of the existing methods with regard to these parameter choices with regard to accuracy and run time?

- L542-543: inconsistent structure of sentence

- The code on github is only available after login. Furthermore, documentation of the code could be improved; the high-level description of the methods is provided, yet the actual implementation is partially not commented at all, making it hard to understand the details.

Reviewer #2

(Remarks to the Author)
MSBayesImpute is an elegant solution and relies on a solid theoretical foundation. It is implemented in both R and Python, and is available as a Shiny app and a Docker image, providing easy access to both bioinformaticians and scientists who prefer a graphical user interface. The authors benchmark their method extensively against eight state-of-the-art missing value imputation methods.

I have one major comment and several minor comments. The major comment relates to the fact that, as of 2025, MSImpute seems no longer to be confined to a two-sample experimental design. The minor comments are mainly suggestions to improve the readability even further, and are an expression of my excitement about the work. I would like to congratulate the authors not only on providing an excellent software tool that is grounded in solid statistical theory and addresses a real need in the field, but also on the robustness of the analyses and the clarity of the manuscript. In my opinion, this is a high-quality scientific work, and I highly recommend publication in Communications Chemistry, provided that the authors address my comments, which I describe in the attached reviewer report.

Reviewer #3

(Remarks to the Author)
The authors describe a novel imputation method, msBayesImpute, for proteomics data. It is made available both as R and python packages, as well as through a shiny app. The method looks very promising compared to other imputation methods

in simulation datasets and in new experimental data.
I still have some major concerns:

Missing value imputation incorporates a lot of guessing and should in general be avoided, but is still needed in some circumstances, as the authors discuss well in the introduction. However, they should also refer to recent work by Li and Smyth , https://doi.org/10.1093/bioinformatics/btad200 and the preprint https://doi.org/10.1101/2025.04.28.651125 when putting their work into context.

While simulated data may be needed, they come with assumptions that do not always reflect real data. The final evaluation using new data is thus very important for assessment of the algorithm and whether it is widely applicable. The approach to inject less of all samples and compare them to LC-MS/MS runs with more of the samples injected is interesting, but I am not sure that it actually reflects real-life scenarios, where it is more likely that different amounts of protein may be analyzed for different samples. It would thus be very beneficial for the study to evaluate the algorithm also using at least one more publicly available experimental dataset. Dilution series experiments or mixed organisms as in Li et al 2025 could here help to assess the algorithm. Specifically, it is important to evaluate TPR and FPR and see how differential expression analysis is affected using such data and also to compare results to data without imputation.

In the final experiment (Figure 5, Table 1) it would be very interesting to include baseline data without imputation, only performing VSN or cyclic loess normalization and see how it compares. As LIMMA is used for the testing, this should work fine, both for the undiluted and the diluted data.

The experimental data should be made available through ProteomeXChange. Currently only the processed data are available as an R object, and preprocessing cannot be followed.

Were all data files processed and normalized together in the final experiment, or how was it performed? Please describe this in the methods section, as it will affect the evaluation.

In most studies normalization is performed before imputation, could the authors describe if their approach of imputation before normalization gives better or worse results compared to imputation after normalization?

Version 1:

Reviewer comments:

Reviewer #1

(Remarks to the Author)
The reviewers have successfully addressed most of my comments, and I therefore basically recommend a publication.
On a side note, the reviewer very much appreciates the very clear and structured form of the rebuttal letter and marking of changes made!

There are only few remaining points to be clarified:
- L163-165: Computational time increases significantly for larger sets at non-linear rate. A one-liner to explain this behavior, e.g. linking to the complexity of the method, would help here to point at potential limits or future optimization directions.
- L544: \bar{U} can only be R^N\times D.
- Response Figure 2: Why do MinDet, QRILC, KNN and few others not show any run time values?
- L248: typo "misssing"
- L 250: running time --> run time
- Suppl. Figure 7: typo "opitmized"

Reviewer #2

(Remarks to the Author)
The authors have addressed all my comments. I would like to congratulate the authors on their hard work and recommend publication in Communications Chemistry.

I just have one minor comment, which I believe the authors can address during the proofing stage:

- Can the authors please double-check line 340 - 341: "...medians derived from msBayesImpute-imputed data were tightly centered around zero and exhibited..."? I think Fig. 5e,f shows the difference between the values from the original undiluted dataset and the imputed or unimputed values of the dataset where missingness was introduced. I think they mean to say that this difference is tightly centered around zero, so I think they'd rather mean to say something like this: "...medians derived from msBayesImpute-imputed data were tightly centered around their true value and exhibited..."

Reviewer #3

(Remarks to the Author)
The authors have addressed all my concerns in a satisfactory way.

Open Access This Peer Review File is licensed under a Creative Commons Attribution 4.0 International License, which permits use, sharing, adaptation, distribution and reproduction in any medium or format, as long as you give appropriate credit to the original author(s) and the source, provide a link to the Creative Commons license, and indicate if changes were made.
In cases where reviewers are anonymous, credit should be given to 'Anonymous Referee' and the source.
The images or other third party material in this Peer Review File are included in the article's Creative Commons license, unless indicated otherwise in a credit line to the material. If material is not included in the article's Creative Commons license and your intended use is not permitted by statutory regulation or exceeds the permitted use, you will need to obtain permission directly from the copyright holder.
To view a copy of this license, visit https://creativecommons.org/licenses/by/4.0/

Item-by-item response to reviewers' comments for " msBayesImpute: A Versatile Framework for Addressing Missing Values in Biomedical Mass Spectrometry Proteomics Data" by J.He et al.

Reviewer #1:

The authors present a novel approach to data imputation for mass spectrometry proteomics data. These data sets typically exhibit gaps due to MAR and MNAR cases, which hinders the application of various analysis techniques. Given synthetic and few actual "real-world" data sets, the authors demonstrate the capabilities of their Bayesian inspired approach in terms of accuracy and run time which both are reported to be comparable or superior compared to existing methods. The manuscript is written all in all well. The topic is of relevance in the field, the statistical analyses are mostly carried out in a sound way.

We sincerely thank the reviewer for the time and effort spent on our work and for the positive feedback. We highly appreciate their insightful comments. Based on their suggestions, we have performed extensive additional experiments and conducted additional analyses, as described in the following.

Major Comments:
Nevertheless, several points are not adequately addressed and require clarification before publication. While a detailed list of concerns is provided below, some major points are:
(1) It is not clear to what extent the synthetic data examples are sufficient to underpin the capabilities of the method in current, but particularly also in future data sets. The latter is essential, given the deluge of data we expect in the upcoming years in this sector.

We thank the reviewer for raising the question. In the revised manuscript, we included additional synthetic and real-world datasets. On the one hand, we generated synthetic data with relatively small sample sizes, namely, 6, 12, and 16 samples, as well as large datasets with 500 and 1000 samples, to mimic the range of typical sample sizes in the current and future proteomic studies. Furthermore, we incorporated two more real-world cases: single-cell proteomes from cell stages of the HeLa cell line (Brunner *et al*., 2022), and human samples spiked with E.coli and yeast proteins (Navarro *et al*., 2016). Now our benchmarking data cover multiple acquisition sources, including MaxQuant, DIA-NN, and DIA-Umpire; the sample sizes range from 6 to 561 samples; and consist of both bulk and single-cell data types. **The updated benchmarking results are included in Lines 143-170, 388-401 and 434-452** and are briefly summarised below.

As shown in the Response Figure 1a (main Fig. 2a), for the synthetic data with a very small sample size (n = 6), msBayesImpute could not predict the ground truth parameters for the per-protein probabilistic dropout curves, which is expected as when the data points are scarce, the empirical Bayesian model will automatically regress to a global dropout curve instead of protein-specific dropout curves. As the sample size increases, the ability of msBayesImpute to recapture the ground truth parameters also increases. The inflection point (ρ) can already be well reproduced with 12 samples, while the accurate recapitulation of the slope (ζ) is more challenging and requires more samples (Response Figure 1b-c) (main Fig. 2b-c). Nevertheless, msBayesImpute demonstrates favorable performance in downstream analysis, even for experiments with small sample sizes, e.g., differential expression analysis of human samples spiked with yeast and E.coli (Response Figure 1d) (main Fig. 5e). The AUROC shows good prediction of Yeast and E.coli signals by msBayesImpute with low variability across small sample sizes (5 repetitions per dataset). Overall, msBayesImpute doesn't require large samples to accurately estimate missing patterns, but the accurate modelling of the protein-specific dropout curves requires at least 12 samples. However, the ability to estimate a global dropout curve when the sample size is small is already beneficial for downstream analysis, as it can still reflect the relationship between protein abundance and the probability of missingness.

In addition to the previous lung cancer datasets, We showed that msBayesImpute worked well in another real-world dataset of spiked human samples (Response Figure 1d) (main Fig. 5e). Furthermore, in the single-cell proteomics data (sample = 231), we evaluated the imputation method by building a

multivariate linear model with Lasso penalty and a repeated cross-validation scheme. Response Figure 1e (main Fig. 6c) showed that msBayesImpute outperformed other methods both in the train/test datasets. These results emphasized that our method is also future-proof and can facilitate single-cell proteomic data analysis.

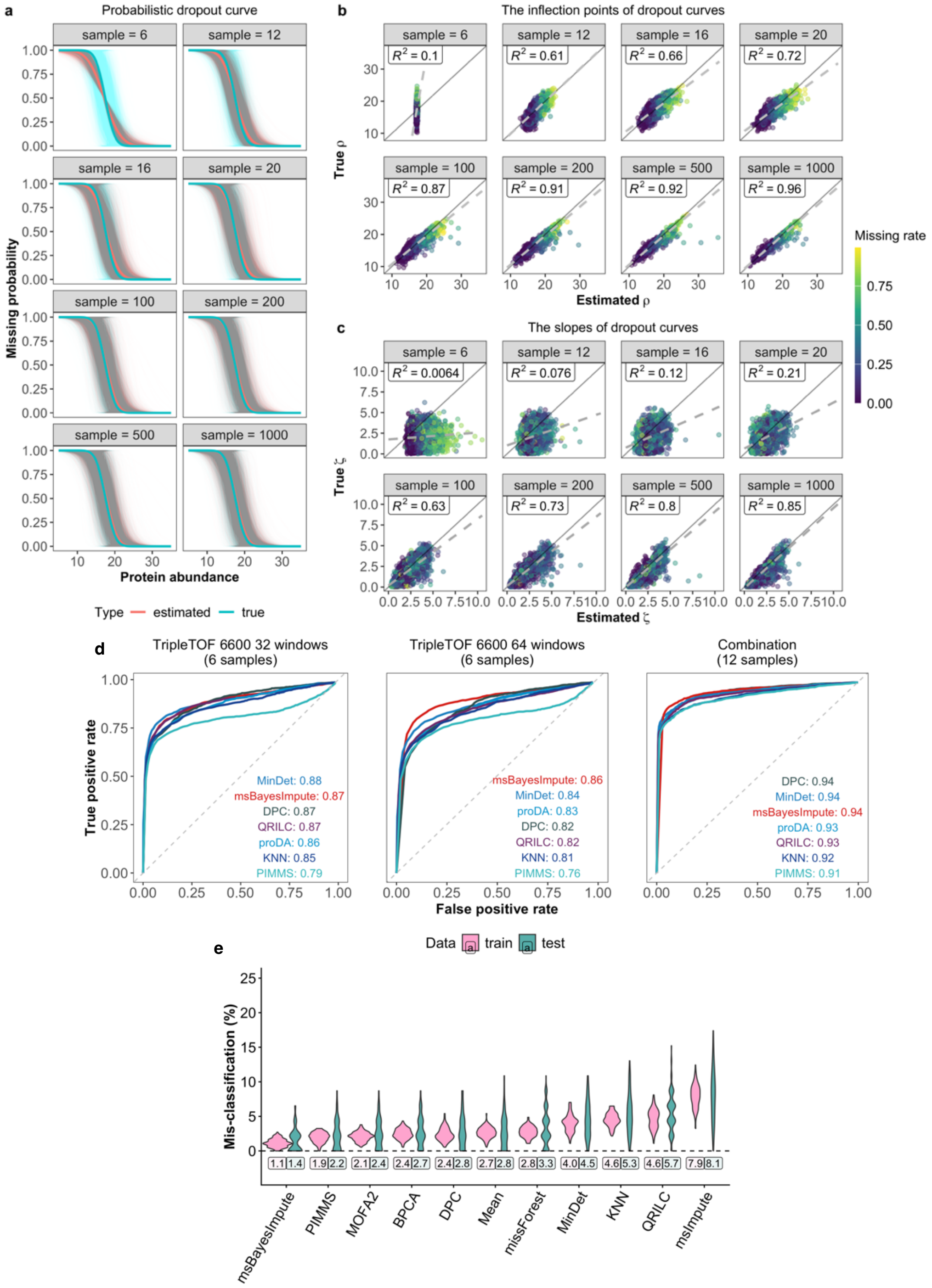

Response Figure 1. Assessing msBayesImpute in synthetic and new public datasets. (a) Comparison of ground truth dropout curves and inferred curves by msBayesImpute from 6 to 1000 samples. (b, c) Scatter plots comparing true versus estimated dropout parameters (ρ and ζ). (d) AUROC values of msBayesImpute alongside the other top seven methods for human samples spiked with yeast and *E. coli* proteins across two experiments with 6 samples each and one experiment with 12 samples. (e) Multivariate linear models with Lasso penalty and a cross-validation scheme were used to predict cell stages using single-cell proteomes across 11 methods. The y-axis represents the misclassification error, with results shown separately for the training and test datasets for each method.

(2) Some notations are mathematically sloppy and need to be corrected.

We thank the review to bring this up. We carefully reviewed our notions and formulas and corrected them in the **revised Methods section (Lines 532-616)**.

(3) The authors claim their method is parameter-free. Looking into its details, it seems to the reviewer that there are parameters, but the authors simply froze them to fixed values. Similarly, for the comparison with other methods, those methods were also fixed in terms of their parameter choices. While these choices might be chosen in a reasonable range, it renders the comparison only partially valid. This issue becomes further pronounced by choosing mostly R-based methods which are then compared to actual Python code (which is only wrapped so that it can be included in the R environment), which is not necessarily fair considering run time comparisons. This comparison requires at least a more solid discussion, or optimally a more detailed evaluation and discussion of parameter spaces and choices. Furthermore, some of the methods, that the comparison is carried out with, could also be used to evaluate gappy data or a minimum set of data extracted from the gappy set -- well aware, that this will not necessarily shed perfect insight into the biology, this can still yield valuable insights into errors the methods make by filling in gaps.

We thank the reviewer for raising this important issue.

Firstly, we agree our model is not paramter-free and we revised related text **(Lines 116-118 in the revised manuscript)** to avoid confusion. What we want to emphasize is that we tried to reduce the parameters users need to tune to achieve a good performance. For the hyperparameters, e.g. priors, we tested different values with synthetic data and fixed them to a sensible range comparable to similar models, e.g. MOFA (**Lines 651-668**). A critical tunable parameter is the factor number. Our model can automatically determine latent factors based on a variance-explained threshold, or users can specify a fixed number of components according to their study design. The 1% explained variance threshold is consistent with that used in MOFA2 to enable fair comparison. In addition, the convergence mode affects the stopping criterion during training, but the differences between modes are minimal. We have also introduced a new parameter, seed, to ensure reproducibility. (**Lines 670-690**)

Overall, msBayesImpute can run well with default parameters for different benchmarking datasets. For the other imputation methods included in this study, parameters were generally kept at their defaults. We did not extensively tune parameters across datasets in order to assess the robustness of each method across different datasets. An exception is KNN, which requires specifying the maximum missing percent per column/sample due to implementation constraints. (**Lines 701-731**)

To further ensure a fair comparison of running time, we updated all packages in R. Although BPCA and missForest have Python implementations, the most recent Python version of missForest by Yuen, S. Y. H. *et al* 2024, is substantially slower than the R implementation, and earlier Python versions are no longer maintained. For BPCA, we compared the Python and R implementations (Response Figure 2) (main Fig. 2k); the R version showed higher prediction accuracy with comparable computational time to the Python implementation by Diedrich, L. 2026. Therefore, the Python version of BPCA was excluded from the subsequent benchmarking. (**Lines 242-246**)

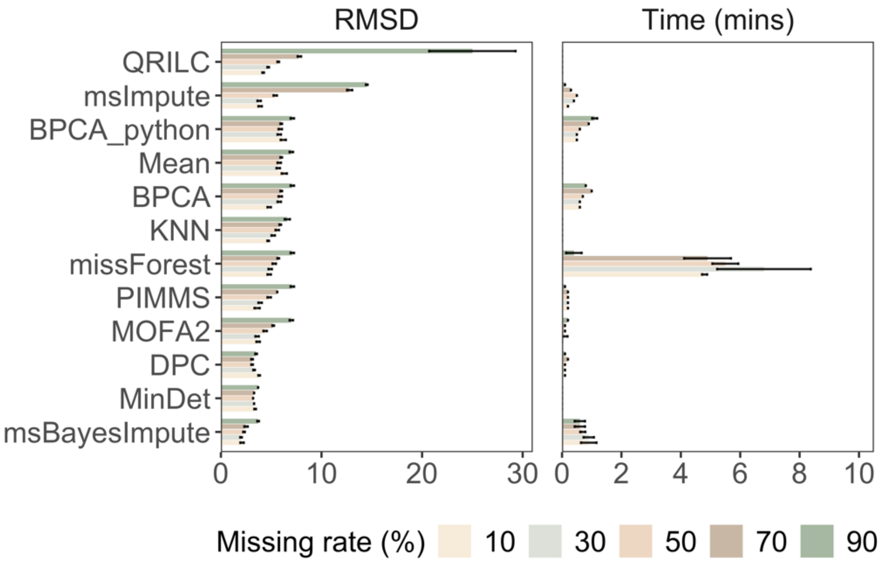

Response Figure 2 Benchmarking using synthetic data with missing rates varing. Root mean square deviation **(**RMSD) and runtime between the imputed and simulated ground truth values. The bars show the averaged RMSD/runtime over 5 random repetitions, and the error bars indicate the 95% confidence interval (CI).

Minor Comments:
Further and more detailed comments:
- L61: "addressing missing values MS proteomics data is imputation," -> weird sentence structure, please re-phrase

We thank the reviewer for this suggestion. We have rephrased the sentence in the manuscript to "*a common approach for addressing missing values in MS proteomics data is imputation*" **in Line 60-61.**

- L145: "Each matrix contained 5,000 proteins and 10 latent factors, with a median protein abundance of 20 (see Methods). To assess the effect of sample size, we simulated datasets with 20, 100, and 200 samples. Missing values were then introduced at rates of 10%, 30%, and 50% using a probabilistic dropout function that assigned distinct missingness patterns to proteins." -> in which regard is this choice covering the relevant range of typically missing value patterns? Higher missingness is not uncommon. How can it be guaranteed that the method still performs reasonably well under such and other extreme conditions, that are out of range of the provided parameters? Also, how well does it perform for bigger data sets in terms of accuracy but also run time?

We thank the reviewer for raising this point. In a study published by Albrecht *et al* in 2010 pointed out that the missing rate varies between 10 and 50% in the case of LC-MS/MS approaches, which is largely in line with our observations. That's why in the primary benchmarking, we only generated missing rates within 50%. To further evaluate the stability of msBayesImpute, we also generated missing rates ranging from 10 to 90% by 10% (sample = 50). msBayesImpute showed a slow increasing of errors within 70% missing values (Response Figure 3a) (Supplementary Fig. 1c). For benchmarking, we created artificial datasets with 70% and 90% missingness (Response Figure 2) (main Fig. 2k). As observed in the first experiment, msBayesImpute performed consistently between 10% and 70% missingness, but showed a marked increase in errors at 90%.

Regarding the accuracy and running time when the sample size increases significantly, we enlarged the sample size to 500 and 1000 samples, although in biomedical research, samples up to 1000 are not common. In Response Figure 1a-c (main Fig. 2a-c), the accuracy of dropout parameters increased slowly from 100 to 1000 samples, which can also be observed with respect to the RMSD of missing values in Response Figure 3b below (Supplementary Fig. 1b). However, the running time rose

significantly in comparison to 500 and 1000 samples. In benchmarking using 1000 samples, (Response Figure 3c) (Supplementary Fig. 1e), while msBayesImpute wasn't the most computationally efficient one, it still outperformed all other methods in terms of imputation accuracy and the run time is in an acceptable range (~10 mins, similar to DPC).

Altogether, msBayesImpute doesn't require a large sample size and outputs quite stable predictions within 70% overall missing rate. **The new bechmarking results are described in Line 156-170, 247-254 in the revised manuscript.**

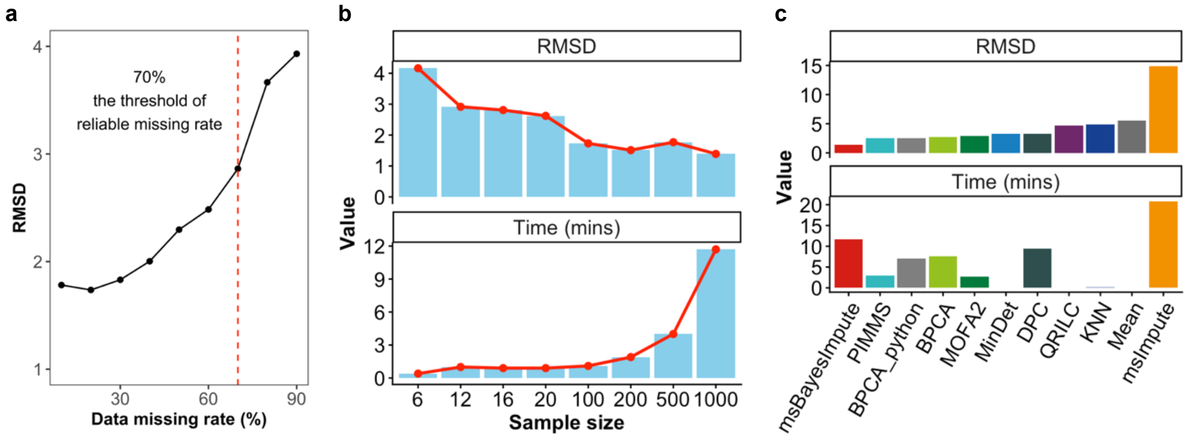

Response Figure 3 Assesssing msBayesImpute with respect to missing rates and sample sizes. (a) RMSD of msBayesImpute across 10–90% missing values. A 70% missing rate is indicated as the reliability threshold for msBayesImpute. (b) Sensitivity of msBayesImpute to sample size, shown with respect to both RMSD and computation time. (c) As of b, eleven MVI methods were compared using the 1000-sample synthetic data.

- Fig 2f,g,h,i: what is the x-axis showing in detail?

The x-axis is the sample-wise medians from the ground-truth dataset without missing values, and the y-axis is the estimated medians from the gappy dataset or imputed values from the gappy dataset. We have revised the legends of those figures (now figure 2d-2i) to clarify the meaning of the x-axis.

- L219: We generated a series of data matrices with MNAR proportions of 0%, 25%, 50%, 75%, and 100% for both the smaller (N=50) and larger (N=561) datasets after filtering out proteins with greater than 75% missing values and samples with greater than 50% missing values." -> what does 100% mean here? Not clear! Further it would be interesting, how the method performs on the entire data set, including the ">75%" proteins and ">50%" samples cases!

We thank the reviewer for this suggestion. Here, "100%" indicates that artificial missing values were generated entirely under a pure MNAR mechanism, rather than a mixture of MNAR and MAR, following the missing value generation model described by Webel *et al.* (2024a) for benchmarking PIMMS. **We have now clarified the meaning of "100%" in the revised manuscript (Line 271).**

To ensure a fair comparison and reproduce the workflow of Webel *et al.* (2024a), we filtered out samples and proteins with more than 50% and 75% missingness, respectively. As suggested, we also repeated the analysis using the full datasets and observed that the relative performance ranking remained unchanged (Response Fig. 4a,b). For simplicity, these additional results are included in the response letter but not in the revised manuscript.

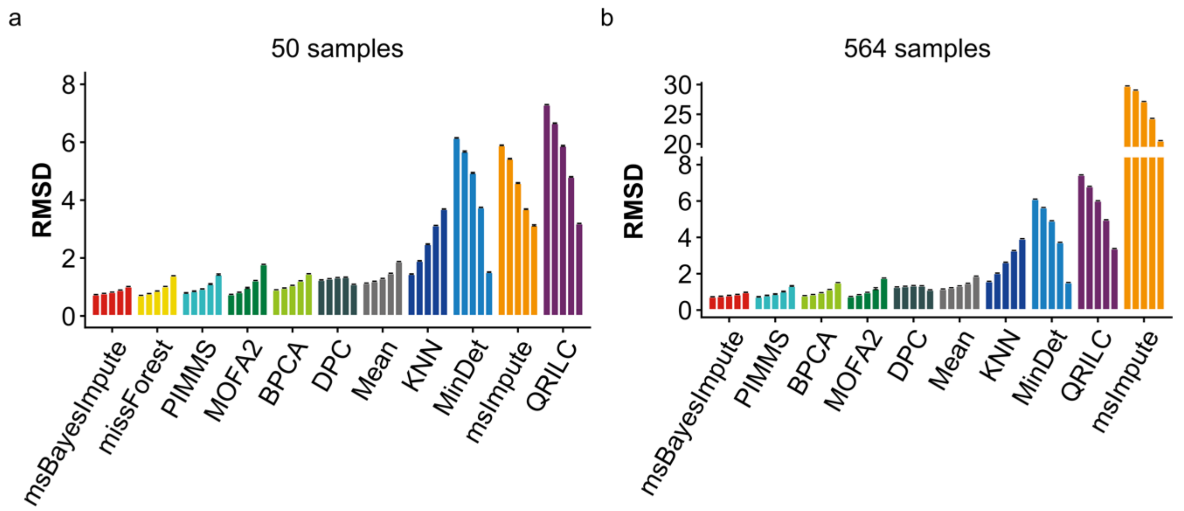

Response Figure 4 Accuracy in semi-synthetic HeLa cell lines (no prefiltering). (a-b) RMSD required by the eleven MVI tools for 50 and 564 samples of HeLa cell line proteomes without filtering high missing proteins/samples.

- Fig3: What are the run times in these practical applications? Are they comparable to the ones mentioned for the artificial data set?

We thank the reviewer for this suggestion. We have now included additional runtime comparisons in the revised manuscript. As shown in Response Fig. 5a–b **(also presented as Supplementary Fig. 2a–b),** the runtime of msBayesImpute on the HeLa cell line dataset is <1 minute for 50 samples and approximately 2–3 minutes for 561 samples. For the 20 lung cancer tissue samples, Response Fig. 5c–d **(also Supplementary Fig. 3a–b)** shows runtimes of approximately 1–2 minutes. We further evaluated two additional public datasets: a single-cell proteomics dataset of HeLa cells across cell cycle stages (231 samples), and a mixed-species dataset of human samples spiked with E. coli and yeast (6 and 12 samples). In both cases, msBayesImpute required approximately 1–2 minutes to complete.

Overall, these runtimes in practical datasets are consistent with the scaling trends observed in synthetic benchmarks **(Response Fig. 3b; also Supplementary Fig. 1b).**

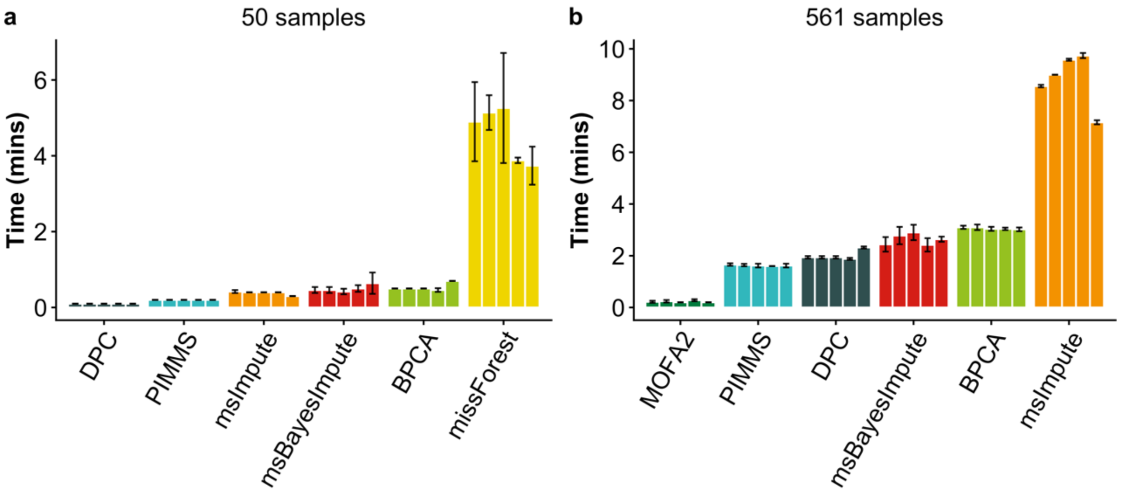

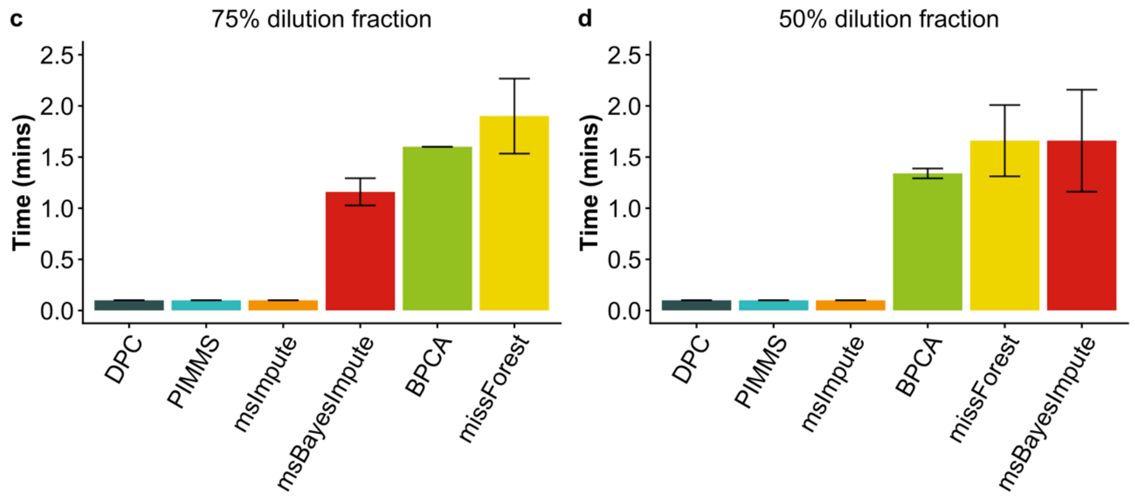

Response Figure 5 Computation time in semi-synthetic HeLa cell lines and lung cancer tissue samples. (a-b) Time required by the six most time-intensive MVI tools for 50 and 561 samples of HeLa cell line proteomes. (c-d) Same assessment as in (a–b), but for lung cancer tissue samples at 75% and 50% dilution fractions.

- L412: "X [bar] is a vector…" -> this cannot be true, same for U [bar]. Both can only be matrices of shape N x D, which might have identical rows/ columns, respectively. The authors should be concise in the notation. Also, dimensions of W and Z are not provided in the section.

We thank the reviewer for this suggestion. We have rephrased the sentence in the manuscript (**Line 539-545**) to "*Where* $\boldsymbol{W} \in R^{D\times K}$ *denotes the weight matrix that maps latent factors to the observed feature space, and* $\boldsymbol{Z} \in R^{N\times K}$ *denotes the factor matrix that contains the low-dimensional latent representations of the samples.* $\overline{\boldsymbol{X}} \in R^{D\times N}$ *is a matrix with identical columns …* $\overline{\boldsymbol{U}} \in R^{D\times N}$ *is introduced as a matrix of indentical rows*".

- L458: "The combinations of (100, 10) for the gamma distribution of the variance m and (200, 10) for the rate z, indicated in equations (7) and (10) led to the best and most stable performance." -> how has this been deduced? The authors should provide proof for this and an explanation how this is justified for arbitrary data sets.

We thank the reviewer for this important point. Selecting hyperpriors for hierarchical Bayesian models is indeed non-trivial. To address this, we have now included a grid search procedure to guide the selection of the three fixed hyperpriors using simulated data. We found that while different combinations yield broadly similar performance, certain settings provide a modest advantage. This procedure is now described in the **revised Methods section (Line 651–668) and illustrated in Supplementary Fig. 6.** The added text is as follows:

"A grid search was performed over three fixed hyperpriors: the rate parameter m *for the inflection parameter* ρ_d*, the rate* z *for the scale parameter* ζ_d*, and the rate* b *for the precision parameter* τ_d*. Candidate values ranged from (1e−14, 1e−14), representing diffuse ("flat") priors, to (2000, 100), corresponding to more concentrated ("narrow") priors. Five simulated datasets (20 samples, 5000 features) with known ground-truth parameters were generated following the procedure described in the* Data Simulation *section.*

Performance was evaluated based on the ability to recover the true parameters (ρ, ζ, τ) and overall imputation accuracy, quantified using RMSD between inferred and ground-truth values. Results for all parameter combinations are shown in Supplementary Fig. 6. Some combinations demonstrated improved recovery of ζ and τ (left panel; green lines), particularly when z ≠ (1, 1) and b = (1, 1).

Among these, we further prioritized combinations that yielded stable performance across simulations, as indicated by lower variability in RMSD (right panel; red lines).

From these candidates, we selected (100, 10) for m, (200, 10) for z, and (1, 1) for b (highlighted in blue), as this combination achieved the lowest imputation error while maintaining stable performance. Although the grid search is not exhaustive and cannot cover all possible real-world scenarios with varying sample and feature sizes, our results suggest that the choice of hyperpriors does not substantially affect performance as long as they fall within reasonable ranges."

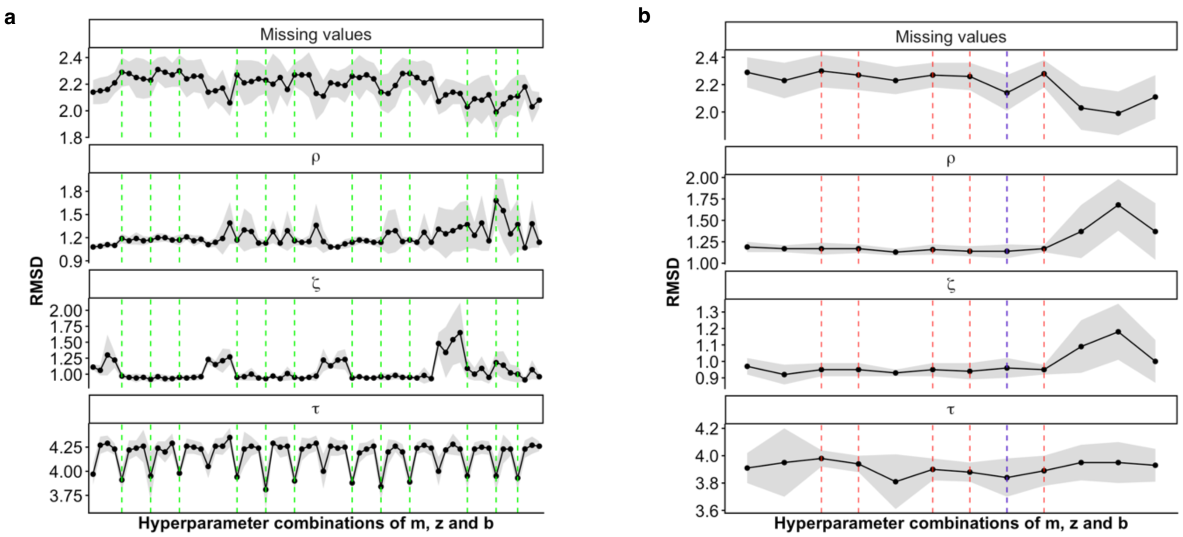

Response Figure 6. Grid search for hyperparameters m, z, and b. (a) Root mean squared deviation (RMSD) between true and inferred parameter values across different combinations of the three hyperpriors. Green lines indicate combinations that achieve relatively optimal performance for the rate parameter z (scale parameter ζ) and b (precision parameter τ). (b) Refinement of candidate combinations identified in (a). The grey shading represents the standard deviation of RMSD across the five simulated datasets. Red lines denote combinations with the lowest variability (i.e., most stable performance), and the blue line highlights the final selected combination, which achieves the lowest imputation error among these candidates.

- L463-464: Eq. 13-16: In L114-115, it is argued that the presented method does not require parameter tuning. Considering Eq 13-16 and following modeling assumptions, various variables have been chosen by the authors fixed, but are actually tunable. The authors should re-phrase their statement in L114-115 and motivate the parameter choices, as well as potential variations thereof.

We thank the reviewer for this suggestion. We have revised the sentence L114-115 into: "To facilitate broad adoption, msBayesImpute is available in both R and Python, requires no complex parameter tuning or predefined experimental design, and can be accessed through an interactive Shiny application." (**now Lines 116-118 in the revised manuscript**).

To further clarify parameterization, we now provide a detailed description of the hyperparameter tuning strategy in the Methods section **(Lines 651–668),** along with a new **Supplementary Figure 7** illustrating the algorithm and parameter inference workflow. We also explicitly describe the key tunable parameters, including the number of latent factors (either user-defined or automatically determined via a variance-explained threshold; **Lines 685–690**), the convergence mode (**Lines 675–677**), and the random seed for reproducibility (**Lines 677–678**).

In addition, we have expanded the documentation in both the R and Python package vignettes to guide users on how to select and interpret these parameters in practice.

- L493: "Additionally, if variances differ significantly across features, but the sample size is too small for the model to capture this variation, an additional initialization step is applied." -> which additional initialization step? What is being done in this case?

We thank the reviewer to point this out. We added a description about this additional initialization step to estimate the hyperprior for the protein-specific precision parameter , τ_d, **in the revised manuscript (Line 594-603):** *"An uninformative prior was initially used for the hyperparameter c governing the protein-specific precision τ_d, following a strategy similar to MOFA (Argelaguet et al., 2018). However, in settings with small sample sizes and high levels of missingness, such uninformative priors can lead to convergence issues. To address this, msBayesImpute estimates an initial global residual and uses it as an informative prior to replace c in Equation (16). Specifically, msBayesImpute first performs principal component analysis (PCA) on data imputed using MinDet. It then derives a noise matrix by removing components that explain more than 5% of the variance. Sample-specific variances are subsequently computed from this noise matrix and averaged to obtain a global residual, which serves as an informative prior for τ"*

- L498-504: this is a very high level description of an iterative procedure. The authors should provide a sketch of their algorithm, incorporating the most essential mathematical steps and this iterative procedure in the manuscript for the sake of self-containedness.

We thank the reviewer for this very helpful suggestion. We have now included a sketch of our algorithm as below (also included as the new **Supplementary Fig. 7**).

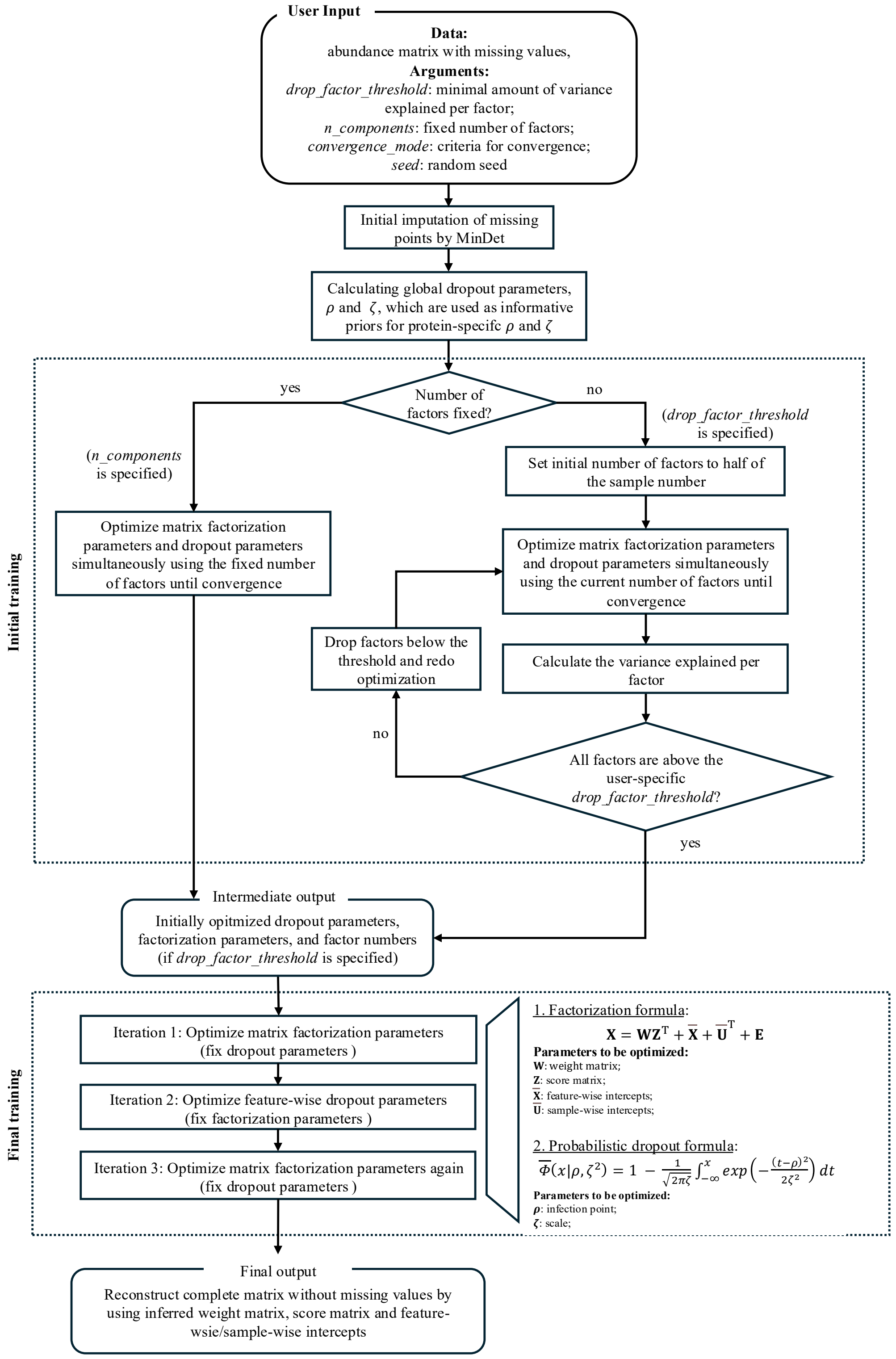

Response Figure 7: Overview of the msBayesImpute algorithm.

- L511-512: "All methods, except for PIMMS, which was only available in Python, were accessible in R." -> this should also be stated when run times are compared. R is sometimes rather slow, a comparison with mostly R-based code sounds like an apple-and-oranges comparison.

We thank the reviewer for this suggestion. **We added this statement in our revised manuscript regarding the run time comparison (Line 242).**

- L522-531: here the parametrization of the other approaches that msBayesImpute is compared with are laid out. How sensitive are the reported data of the existing methods with regard to these parameter choices with regard to accuracy and run time?

We thank the reviewer for highlighting this issue. Default parameter settings were primarily used for all comparator methods. To reflect realistic biomedical applications, where ground truth is typically unknown, parameters were not tuned across datasets. This approach avoids data leakage and enables an unbiased evaluation of predictive performance. Detailed descriptions of parameter choices that may influence the performance of comparator methods have been added to the revised manuscript **(Lines 702–731).**

- L542-543: inconsistent structure of sentence

We have polished the sentence in the revised manuscript (**Lines 627-630**) to *"One method generates missing values with the probabilistic dropout function on which msBayesImpute is based, where feature-wise inflection and scale parameters are drawn from a Gaussian distribution with a chosen location and standard deviation"*.

- The code on github is only available after login. Furthermore, documentation of the code could be improved; the high-level description of the methods is provided, yet the actual implementation is partially not commented at all, making it hard to understand the details.

We thank the reviewer for this constructive comment. We have made our repository publicly available, and note that accessing and viewing the code does not require a GitHub login. In addition, we have improved the documentation by adding more detailed comments throughout the source code in both the R and Python implementations to enhance readability and usability.

Reviewer #2:
MSBayesImpute is an elegant solution and relies on a solid theoretical foundation. It is implemented in both R and Python, and is available as a Shiny app and a Docker image, providing easy access to both bioinformaticians and scientists who prefer a graphical user interface. The authors benchmark their method extensively against eight state-of-the-art missing value imputation methods.

I have one major comment and several minor comments. The major comment relates to the fact that, as of 2025, MSImpute seems no longer to be confined to a two-sample experimental design. The minor comments are mainly suggestions to improve the readability even further, and are an expression of my excitement about the work. I would like to congratulate the authors not only on providing an excellent software tool that is grounded in solid statistical theory and addresses a real need in the field, but also on the robustness of the analyses and the clarity of the manuscript. In my opinion, this is a high-quality scientific work, and I highly recommend publication in Communications Chemistry, provided that the authors address my comments, which I describe in the attached reviewer report.

We sincerely thank the reviewer for the positive feedback and for their insightful comments. Based on their suggestions, we have performed extensive additional experiments and analyses, as described in the following.

Major comment
1. On multiple occasions, the authors write that MsImpute is limited to two-group experimental designs. See e.g. Line 82: "Another approach, MsImpute (Hediyeh-Zadeh, Webb and Davis, 2023), can automatically identify protein subsets adhering to MAR or MNAR mechanisms, though its application is confined to two-group experimental designs. " Line 267: "We next compared msBayesImpute with nine other MVI methods, including MsImpute, which models MAR/MNAR mixtures but is restricted to two-group experimental designs (e.g., tumor vs. tumor-free). " At present, this statement is no longer correct. The 2023 manuscript that describes Msimpute (Hediyeh-Zadeh et al MCP) states "Note that although 2-group designs were considered in the DA benchmark analyses covered here, the method is applicable to complex experimental designs (e.g., time-series, multiple conditions, combination of groups and time course, etc.) if the experimental groups of interest can be discretized. " Since March 2025, their GitHub page now deprecates the use of the "group" argument in favor of a design matrix, which indicates that MsImpute can now also handle more complex experimental designs. Hence, unless there is another reason, it would be a good idea to include MsImpute also in the analyses in Fig. 2 and Fig. 3.

We thank the reviewer for raising this point. We updated our manuscript text to reflect the fact that MsImpute accepts complex designs via a design matrix rather than only accepting a two-group design, and incorporated MsImpute into the benchmarking on synthetic and semi-synthetic datasets. The benchmarking results of MsImpute on synthetic data are shown in Response Figure 2 **(main Fig. 2k),** and those on the semi-synthetic data are shown in Response Figure 8a-b **(main Fig. 3a-b).** We also need to point out that, based on the implementation of MsImpute, when no experimental information is available, MsImpute fixed the proportion of MAR/MNAR across all proteins instead of learning this proportion in a data-driven way, which significantly reduces imputation accuracy, as shown in our benchmarking results.

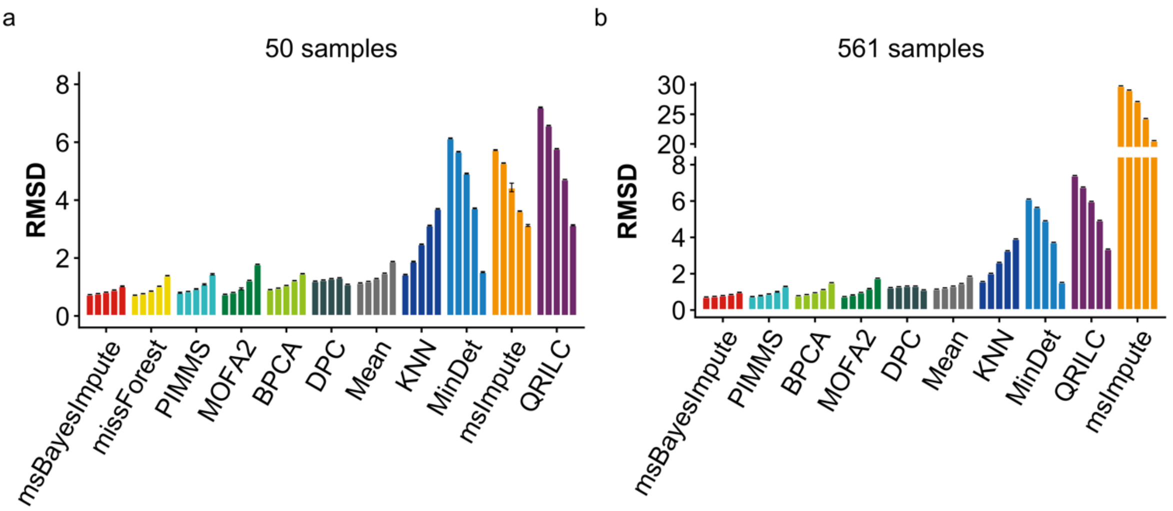

Response Figure 8 Benchmarking msBayesImpute on semi-synthetic published HeLa cell line proteomics data with varying MNAR proportions. (a) Reconstruction accuracy of nine imputation methods across datasets with MNAR proportions ranging from 0% to 100% (25% increments from left to right), based on RMSD between imputed and ground truth values in the smaller dataset (*N*=50). **(b)** Performance of the same methods on the larger dataset (*N*=561), excluding missForest, which failed to converge.

Minor comments

2. Fig.1: I would somewhere specify that (I assume): ρ is the inflection point, and ζ is the scale parameter of the dropout function. I would optionally also specify that $\boldsymbol{\sigma}^2$ is the variance of the Gaussian function (even if that is perhaps more obvious).

We thank the reviewer for this suggestion. We have added the specification in **Fig.1.**

3. A suggestion for clarity. On line 163: the authors write: "Each condition was repeated on five datasets with the same missingness profile. " To me, this sentence came as a surprise, and I could not immediately figure out which datasets the authors were talking about. Only by re-reading and checking Fig. 2d, it became clear to me that these are five simulations. To make this clearer, I would suggest to re-write the sentence on line 156 for example as: "We next benchmarked msBayesImpute against eight commonly used missing value imputation (MVI) methods under varying missing rates (10%, 30%, 50%; five simulated datasets each) with 50 samples per dataset." (or anything else that makes it clear that there are five simulations per missingess rate). I would then simply drop the sentence "Each condition was repeated on five datasets with the same missingness profile. " from line 163.

We thank the reviewer for rephrasing the sentences. We have incorporated the suggested change in the revised manuscript in **Lines 223-225.**

4. Fig. 2a: the authors simulated datasets with 20, 100 and 200 samples. In practice, datasets with 100 or 200 samples are usually already quite large experiments. I was just curious what happens in a very small experiment of, say, 6 samples (i.e. the "classic" 3 control vs 3 treated samples design)? Could the authors add a simulation with 6 samples too, to show the behavior of msBayesImpute in very small experiments?

We thank the reviewer for raising this question. As shown in Response Figure 1a-c (main Fig. 2a-c), for the synthetic data with a very small sample size (n = 6), msBayesImpute could not predict the ground truth parameters for the per-protein probabilistic dropout curves, which is expected, as when the data points are scarce, the empirical Bayesian model will automatically regress to a global dropout curve instead of protein-specific dropout curves. As the sample size increases, the ability of msBayesImpute to recapture the ground truth parameters also increases. The inflection point (ρ) can already be well

reproduced with 12 samples while the accurate recapitulation of the slope (ζ) is more challenging requires more samples (Response Figure 1b-c) (main Fig. 2b-c). Nevertheless, msBayesImpute demonstrates favorable performance in downstream analysis, even for experiments with small sample sizes, e.g., differential expression analysis of human samples spiked with yeast and E.coli (Response Figure 1d) (main Fig. 5e). The AUROC shows good prediction of Yeast and E.coli signals by msBayesImpute across small sample sizes (5 repetitions per dataset). We have now included the updated benchmarking results with very small sample sizes in the revised manuscript (**main Fig. 2a-c, Lines 143-165; main Fig. 5e, Lines 388-401**)

5. In Fig. 2b, the authors show coefficients of determination (R2), which I assume are based on an ordinary linear regression. I was wondering why the authors are plotting the diagonal line instead of the actual regression line. Would the latter not be more informative?

We thank the reviewer for raising this point. The diagonal line is helpful to observe whether estimated parameters accurately recapture the true ones, not just correlating with them, i.e. the intercept should be zero. But we agree that the regression line can also be informative, and therefore **we added it to the revised main Fig. 2b-c (Response Figure 1b-c).**

6. Line 179: I would specify that this is a 95% confidence interval.

We have specified it in the legend of Figure 2k **(Line 219 in the revised manuscript)**.

7. Fig. 2(f,g), in line 180, I would also specify in the legend "derived from ground truth data versus matrices with ~30% missing values".

We have specified this in the legends of Figure 2 (**Lines 213-214 in the revised manuscript)**.

8. On line 192, the authors write: "To assess this effect, we simulated datasets with ~30% missing values. " I was wondering if these are the same datasets with 30% missing values mentioned on line 157? If yes, perhaps the authors could briefly clarify this in the text. If not, could they specify in the text with what method the missing values were introduced?

We thank the reviewer for pointing out this question. The ~30% missing values in the normalization test are different from those in 10-90% simulations. In this case, we simulated a relatively large variation (standard deviation = 3) of sample-specific intercepts with the same 30% missing values in order to test the ability of different MVI tools to recapture this variation. We have now clarified this in the revised manuscript (**Lines 178-180**).

9. Fig. 3d: it is not immediately clear to me how over- and underestimation are calculated. Could the authors clarify this? As a differences closer to zero are better and differences away from zero are worse, I would highlight the 0-level with a horizontal line to clarify this in the figure. I would also clarify in the figure and/or in the figure legend what "differences" means exactly.

We thank the reviewer for raising this question and the suggestion. The zero-horizontal line has been added to the plot in the **main Fig. 3d (also shown as the Response Figure 9 below).** The difference = imputed values – true values, so if the differences are greater than zero, the imputation method has an overestimation issue; otherwise, it has an underestimation error. We have added the explanation in the legend of Figure 3d (**Lines 305-306**).

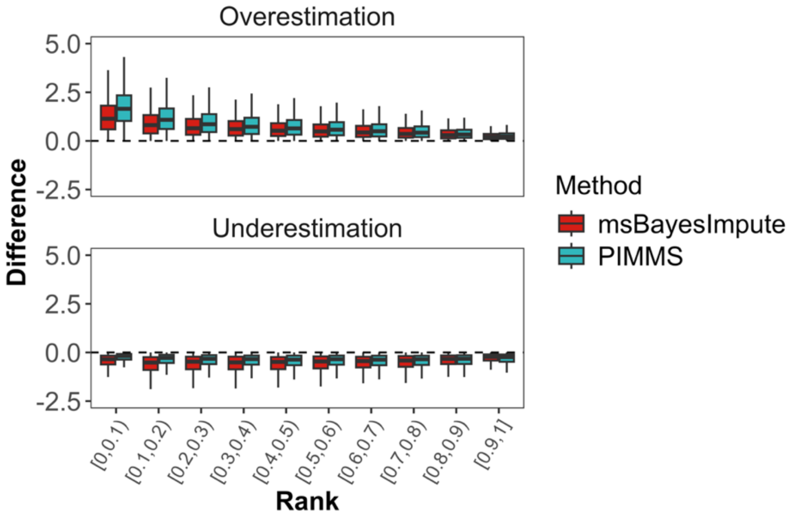

Response Figure 9 Comparing msBayesImpute to PIMMS in terms of protein-specifice error. Protein-level imputation error analysis comparing msBayesImpute and PIMMS, with over- and underestimation quantified across ten ranked protein abundance groups.

10. Line 278: the authors write: "MsImpute yielded comparably accurate normalization, whereas KNN and unimputed data performed the poorest. " I was wondering if the authors actually performed the subsequent normalization or if this is based on MsImpute having a low bias in Fig. 4e and f? For normalization purposes, I could imagine that the most important thing is not the bias, but the variance of the differences shown in Fig. 4e,f. Imagine all the sample median abundances are strongly biased, but with a fixed, constant bias, then I would imagine the subsequent differential abundance analysis will not be biased, because when comparing two samples, the constant biases will cancel each other out. I could be wrong here, but if the authors agree that my interpretation is correct, perhaps they should either (1) reword to specify that they are talking about the variability of the differences, or (2) reword to avoid the word "normalization", or (3) if possible perform some kind of analysis to show that normalization is effectively more accurate here.

We thank the reviewer for pointing out an important issue. We agree that fixed, constant bias won't influence the subsequent result, and the variability of the differences plays a more important role. Therefore, we performed VSN normalization in both ground-truth and imputed datasets, then calculated the bias/ difference between them and centered these points. If the variance of differences is small, i.e., distributed together around the zero line in Response Figure 10 **(main Fig. 4e-f)**, indicating the higher accuracy of normalization following this imputation method. msBayesImpute was still superior to other methods since all points were concentrated near the zero line. **The updated result is now described in Lines 338-344 in the revised manuscript.**

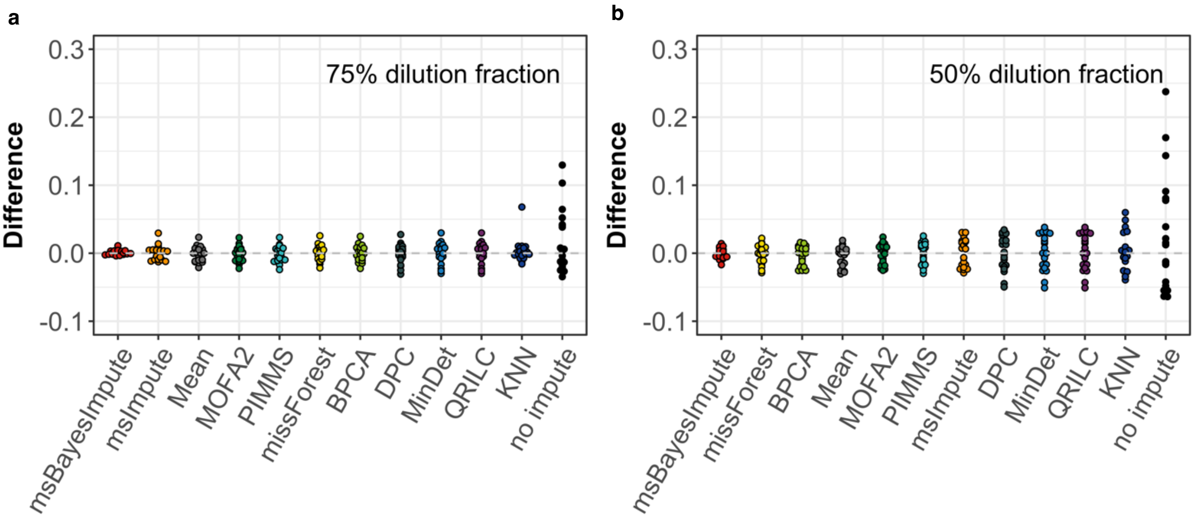

Response Figure 10 Benchmarking msBayesImpute using serial dilution experiments in terms of normalization accuracy. Errors in estimating the variability of sample-wise medians were calculated by comparing values from the undiluted dataset (100%) with those from imputed or incomplete datasets at 75% and 50% dilution fractions. These differences were also VSN and median-centered to provide an intuitive visualization of variability for each method.

11. Purely out of curiosity, and I do not necessarily expect the authors to address this in the manuscript, as it is somewhat out of scope, but I was quite shocked to see in Fig. 5 a and b that the actual FDR remains overall quite stable and, for msBayesImpute, even decreases, over a desired FDR that ranges from 0 to 25%. What do you think would be the explanation for this phenomenon?

We thank the reviewer for raising this important question. In experimental datasets, the true set of differentially expressed (DE) proteins between normal and tumor tissues is unknown. To address this, we defined the "ground truth" as all proteins identified as DE in the complete (undiluted) dataset at a given FDR threshold. As the target (desired) FDR increases, the corresponding set of "ground truth" DE proteins also expands. This leads to increased overlap between the "ground truth" and the DE proteins identified in datasets with missing values at the same FDR threshold. Consequently, the desired-versus-actual FDR curves shown in main Fig. 5a–b differ from the typical scenario where the ground truth set remains fixed.

This also explains the observed pattern: the actual FDR decreases between 0% and 5% desired FDR, reflecting a rapid increase in overlap, and then remains relatively stable between 5% and 25%, where the overlap grows more gradually. An illustrative example is provided in Response Fig. 11.

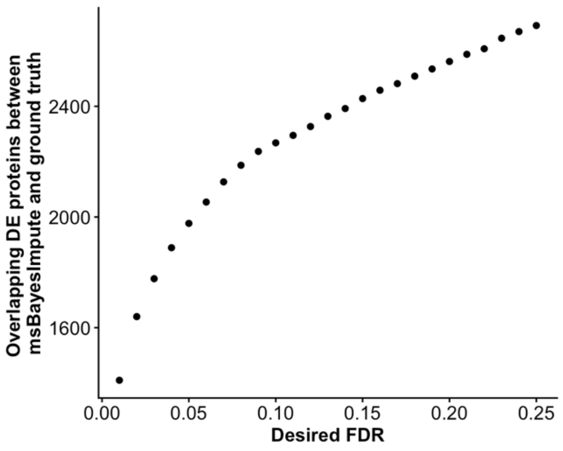

Response Figure 11 Overlapping DE proteins between msBayesImpute and ground truth.

12. In the section from lines 290 – 302, the authors compare DE analyses based on different imputation methods, as well as the standard proDA workflow. However, I think they should explicitly mention in the main text and not only in the Methods section that they used limma for DE analysis for all methods except proDA (which has its own dedicated workflow). Otherwise, I am left wondering from which analysis the FDR values were derived.

We thank the reviewer for the suggestion. We have added this important information in the main text in **Lines 362-364**.

13. In equation (9), is "N+" a positive half Gaussian distribution?

Yes. The positive sampling enables the slope to be positive, indicating the constant negative relationship between protein abundances and missing rates.

14. The authors are very thorough in their discussion of the limitations of msBayesImpute, which I really appreciate. They write: "Its use of a variational Bayesian framework means that uncertainty quantification is generally underestimated (Blei, Kucukelbir and McAuliffe, 2017), which could limit its integration into downstream statistical inference or modelling." Out of curiosity, I was wondering if the software would allow small perturbations of the hyperparameters, such as those of the gamma distribution of the variance (100, 10) and/or the rate zd (200,10) and/or if changing the seed produces different results? This would then allow users to perform multiple imputation. Could such an approach mitigate some of these problems? If yes, maybe it is also an interesting future direction to mention in the discussion section? If not, there is probably no need to discuss this.

We thank the reviewer for this suggestion. Indeed, one way to estimate the uncertainty is through bootstrap resampling with different seeds. However, this process will be time-consuming. One potential danger of underestimated uncertainty is the increased false positive rate in differential expression. Therefore, we paid special attention to it in our benchmarking (main Fig. 5a and 5b). It turns out that our method can control the FDR equally well, if not better than, other methods. Therefore, we think for exploration and hypothesis generation, it's not necessary to very accurately estimate the uncertainty. However, if the users desire, they can choose bootstrapping or other statistical methods for this purpose. **We added this suggestion in the discussion part (Lines 497-501).**

15. Line 617, the authors write: "For the semi-synthetic and experimental data, a log transformation of base 2 was applied to the spectra counts prior to imputation." This comes as a surprise to me. Throughout the manuscript, I assumed the authors worked with spectral intensities (see e.g. line 274). It is known that intensity-based approaches are more powerful than count-based approaches in differential abundance analysis for MS-based proteomics. Could the authors please clarify whether they used summarized protein-level intensities, spectral count data, or something else (e.g. ion-level or peptide level intensities, peptide counts, …)?

We apologize for the unclear description. The HeLa cell line proteome data used in Webel *et al.* 2024b were acquired by MaxQuant, and the Lung cancer tissue samples were derived from DIA-NN. The two new benchmarking datasets are single-cell analysed by DIA-NN and human mixtures with yeast, and E.coli analysed by DIA-Umpire. They are all intensity-based quantification (maxLFQ) rather than spectra count. We have revised this part in the manuscript (**Lines 732-737, 828-841, 845-856**).

Spelling/grammar suggestions
- Line 542: "One method generates missing values with the probabilistic dropout function on which msBayesImpute is based,"
- Line 412: "with the length of D" -> "with length D"
- Line 548: "TMT- or SILAC labelled MS data. " -> "TMT- or SILAC-labelled MS data. "
- Line 557: "…missingness and to avoid bias during benchmarking." -> "…missingness. It also avoids bias during benchmarking. "
- Line 561: "function as missing rate" -> "function, as missing rate"

- Line 564: “For benchmarking, a semi-synthetic dataset, where…”-> “For benchmarking, we used a semi-synthetic dataset where…”
- Line 588: “For sample preparation two slices each” -> “For sample preparation, two slices each”
- Line 674: “which was proven to outperform other commonly used normalisation methods in differential expression tasks, such as the median normalisation approach (Valikangas, Suomi and Elo, 2016). ” -> “which was proven to outperform other commonly used normalisation methods, such as the median normalisation approach, in differential expression tasks (Valikangas, Suomi and Elo, 2016). ”

We thank the reviewer for polishing these sentences (**Lines 672-630, 633, 641-642, 646, 757, 879-881 in the revised manuscript**).

Reviewer #3:

The authors describe a novel imputation method, msBayesImpute, for proteomics data. It is made available both as R and python packages, as well as through a shiny app. The method looks very promising compared to other imputation methods in simulation datasets and in new experimental data. I still have some major concerns:

Missing value imputation incorporates a lot of guessing and should in general be avoided, but is still needed in some circumstances, as the authors discuss well in the introduction. However, they should also refer to recent work by Li and Smyth , https://doi.org/10.1093/bioinformatics/btad200 and the preprint https://doi.org/10.1101/2025.04.28.651125 when putting their work into context.

We thank the reviewer for the time and effort spent on our work. Indeed, the recent work by Li and Smyth (Li and Smyth, 2023; Li, Cobbold and Smyth, 2025), is very relevant in this context. We have thus incoporated their DPC model from the Limpa package into our benchmarking pipeline across all datasets, including synthetic data (Response Figure 2) **(main Fig. 2k**), semi-synthetic HeLa cell lines (Response Figure 8) **(main Fig. 3)**, Lung cancer tissue samples (Response Figure 10, 12) (**main Fig. 4, 5a-d**), single cell proteomics (Response Figure 1e) (**main Fig. 6**), human mixture with yeast and E.coli (Response Figure 1d) (**main Fig. 5e**). While the DPC model showed quite good performance compared to older methods in differential expression tasks, our msBayesImpute still outperformed DPC in those tasks.

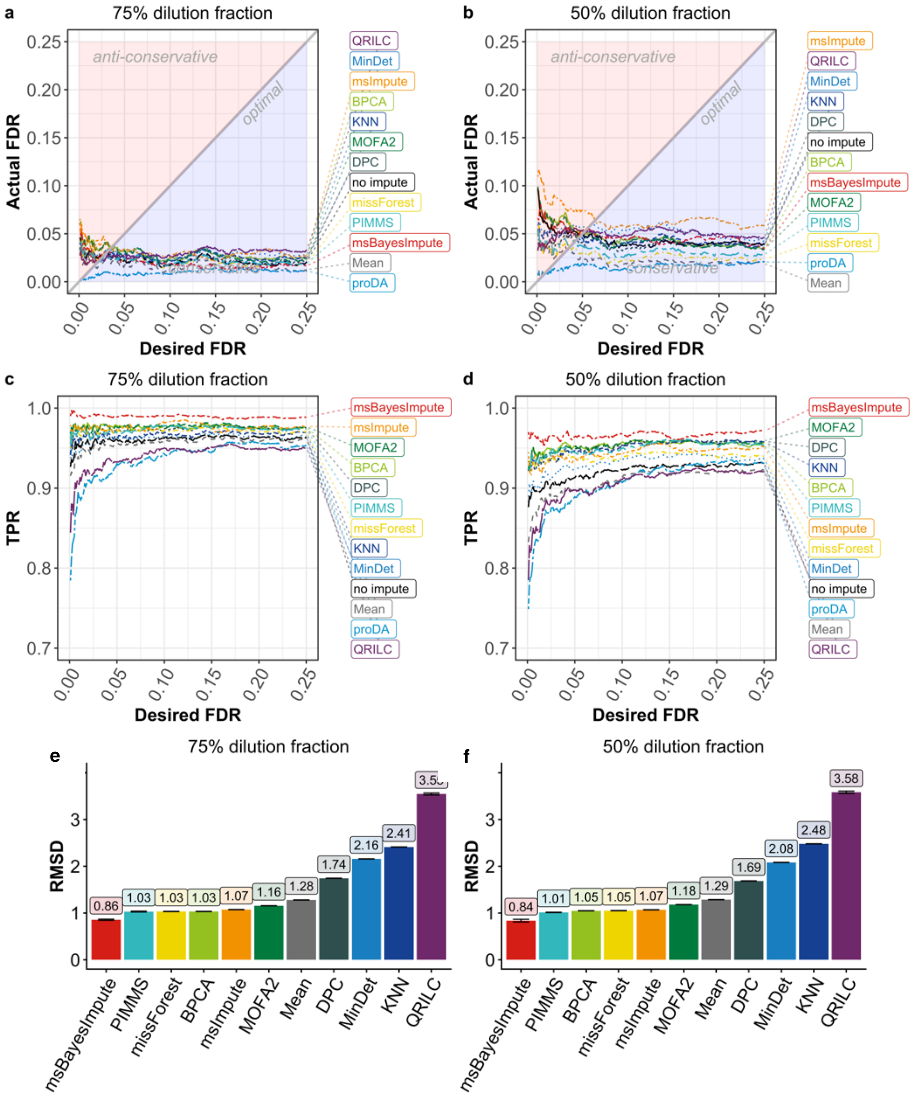

Response Figure 12 Benchmarking msBayesImpute using serial dilution experiments in terms of differential expression analysis and reconstruction accuracy. (a, b) Relationship between desired and actual false discovery rate (FDR) in the 75% and 50% dilution datasets. Note that, due to the lack of a truly differentially expressed protein list, the desired FDR corresponds to the cutoff used to define differentially expressed proteins in the undiluted dataset (ground truth), while the actual FDR reflects the proportion of false discoveries when applying the same cutoff to imputed datasets. Therefore, the "ground truth" protein list will also grow when the desired FDR increases, which leads to a flatter look of the curves. A gray diagonal line indicates the optimal value. Light blue color indicates a conservative FDR, whereas orange indicates an anti-conservative FDR. **(c, d)** True positive rate (TPR) in the 50% and 75% dilution datasets, calculated as the proportion of ground truth positives (from the undiluted dataset) recovered in the imputed datasets. (**e, f**) Reconstruction accuracy of eleven imputation methods, evaluated on unbiased validation data generated from serial dilutions of the original sample concentration (75% and 50% relative to undiluted controls).

While simulated data may be needed, they come with assumptions that do not always reflect real data. The final evaluation using new data is thus very important for assessment of the algorithm and whether it is widely applicable. The approach to inject less of all samples and compare them to LC-MS/MS runs with more of the samples injected is interesting, but I am not sure that it actually reflects real-life scenarios, where it is more likely that different amounts of protein may be analyzed for different samples. It would thus be very beneficial for the study to evaluate the algorithm also using at least one more publicly available experimental dataset. Dilution series experiments or mixed organisms as in Li et al 2025 could here help to assess the algorithm. Specifically, it is important to evaluate TPR and FPR and see how differential expression analysis is affected using such data and also to compare results to data without imputation.

We thank the reviewer for this very helpful suggestion and point us to more benchmarking datasets. We added two new benchmarking datasets: a single cell proteomic dataset with 231 samples, and human mixture with yeast and E.coli of 6 and 12 samples, both also analysed by Li *et al.* 2025. MsBayesImpute showed top performance in both benchmarks. Particularly, msBayesImpute outperformed other methods, including DPC, in recovering cell-cycle stage markers as well as predicting cell-cycle stages, with a single-cell proteomic dataset, highlighting the usefulness of our model in the analysis of single-cell proteomic data, which is the state-of-the-art of mass-spectrometry proteomics. **The updated benchmark results are included in main Fig. 5e, Fig. 6 and Supplementary Fig. 4-5.** As suggested, we also added a "no impute" approach in differential expression analysis in Lung cancer cohorts **(main Fig. 5a-d, Lines 377-387)**

In the final experiment (Figure 5, Table 1) it would be very interesting to include baseline data without imputation, only performing VSN or cyclic loess normalization and see how it compares. As LIMMA is used for the testing, this should work fine, both for the undiluted and the diluted data.

We thank the reviewer for this suggestion. **We have now included un-imputed dataset processed with only VSN in the revised Figure 5 and Table 1.** The updated table below shows that no imputation method was inferior to most of the imputation methods.

Method	75% dilution fraction	50% dilution fraction
msBayesImpute	**0.983**	**0.956**
DPC	0.975	0.953
missForest	0.973	0.954
PIMMS	0.972	0.954

Method	75% dilution fraction	50% dilution fraction
MOFA2	0.972	0.951
BPCA	0.972	0.949
KNN	0.969	0.951
Mean	0.970	0.934
msImpute	0.970	0.931
no impute	0.969	0.931
MinDet	0.967	0.932
proDA	0.961	0.933
QRILC	0.952	0.922

Response Table 1: F1 scores measurements from the serial dilution experiments of the lung cancer proteomes. The F1 score, reflecting the balance between true positives and true negatives, was calculated with the desired false discovery rate (FDR) fixed at 0.05. Thirteen imputation methods were evaluated.

The experimental data should be made available through ProteomeXChange. Currently, only the processed data are available as an R object, and preprocessing cannot be followed.

We thank the reviewer for the suggestion. We have uploaded experimental data to PRIDE (https://www.ebi.ac.uk/pride/) with the accession code: PXD077257. Reviewers can access the dataset using the following account details:
Username: reviewer_pxd077257@ebi.ac.uk
Password: KH75pu6j1W34
The dataset will be made publicly available once the manuscript is published. The access is also provided in the "Data Availability" section in the revised manuscript **(Line 931)**

Were all data files processed and normalized together in the final experiment, or how was it performed? Please describe this in the methods section, as it will affect the evaluation.

We thank the reviewer for this question. In the updated Methods sections, we have added a detailed description (as shown below) of how the lung cancer experiment data were pre-processed **(Lines 828-841)**.

"Protein identification and quantification were performed using DIA-NN (v1.9) in library-free mode with deep learning–based spectral prediction. An in silico spectral library was generated directly from FASTA sequences of reviewed proteins from Uniprot, using tryptic digestion (cleavage at K/R, up to two missed cleavages), allowing peptides of 7–30 amino acids, precursor m/z 300–1800, fragment m/z 200–1800, and precursor charge states of 1–4. Carbamidomethylation of cysteine was set as a fixed modification, while methionine oxidation (UniMod:35) was included as a variable modification (maximum one variable modification per peptide), with peptidoform-level scoring enabled. DIA-NN was configured to optimise mass accuracy and scan window settings independently for each run. Protein inference was performed using heuristic protein grouping. False discovery rate (FDR) was controlled at 1% at precursor and protein levels. Quantification was based on precursor-level intensities without additional normalisation or match between runs to ensure independence of search results of each dilution fraction, and protein group level quantification matrices across samples were exported for downstream analysis in R."

In most studies normalization is performed before imputation, could the authors describe if their approach of imputation before normalization gives better or worse results compared to imputation after normalization?

We thank the reviewer for raising this important point of the order of imputation and normalisation, as this issue has not been addressed before; therefore, we systematically evaluated the impact of the order of the two steps on the accuracy of imputation and differential expression of all methods

The evaluation of imputation accuracy was conducted using synthetic data. In the three different missing-rate data, msBayesImpute showed a consistent improvement by using the impute-first strategy, while the difference is modest (Response Figure 13a) **(Supplementary Fig. 1d)**. Only PIMMS, missForest, and BPCA displayed a large improvement by the normalize-first strategy, and QRILC showed a minor but consistent advantage across three missing rates. Therefore, the four imputation methods applied a post-normalization imputation strategy in the reconstruction/prediction tasks, and the remaining methods imputed before normalization. **This result is included in Lines 194-204 in the revised manuscript.**

The evaluation of differential expression was assessed using the lung cancer tissue data. We compared the t-values from the ground-truth data with the t-values obtained after imputation. Response Figure 13b-c (**Supplementary Fig. 3c-d**) shows the preference across two fractionation profiles for the impute-first strategy (msBayesImpute, msImpute) and the normalize-first strategy (PIMMS, MOFA2, BPCA, KNN, missForest, QRILC, MinDet, and Mean). **The result is included in lines 365-371 in the revised manuscript.**

Based on the above results, we recommend that users to perform imputation before normalisation if they use our method, as the imputation-first strategy showed a consistent advantage over normalisation-first in different scenarios. However, the performance difference is not dramatic, and therefore, if the user can only obtain previously normalized data, our method also works well. **The recommendation is mentioned in Lines 472-474 in the Discussion section.**

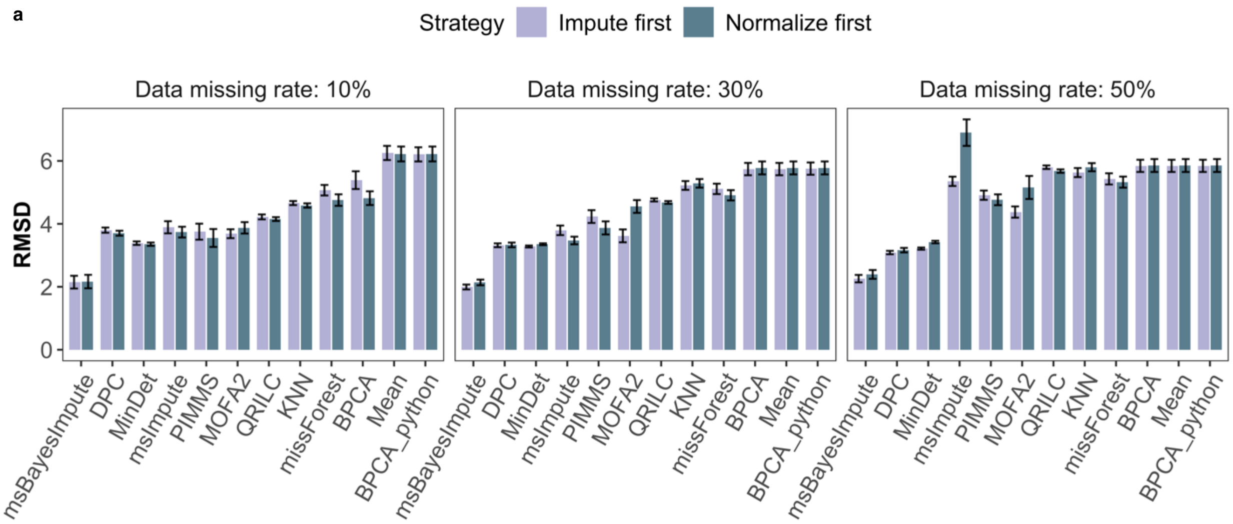

b

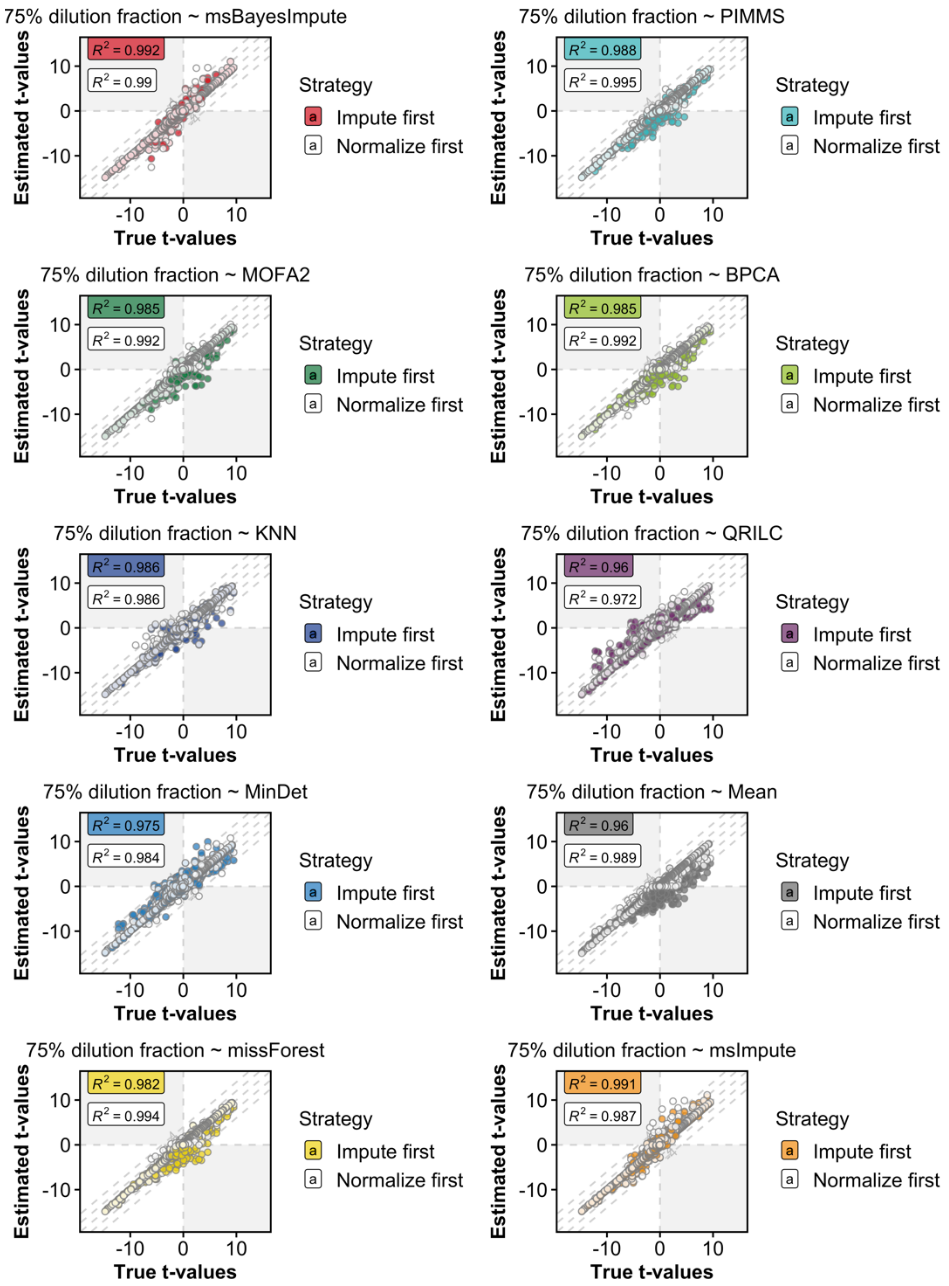
75% dilution fraction ~ msBayesImpute
R² = 0.992
R² = 0.99
75% dilution fraction ~ PIMMS
R² = 0.988
R² = 0.995
75% dilution fraction ~ MOFA2
R² = 0.985
R² = 0.992
75% dilution fraction ~ BPCA
R² = 0.985
R² = 0.992
75% dilution fraction ~ KNN
R² = 0.986
R² = 0.986
75% dilution fraction ~ QRILC
R² = 0.96
R² = 0.972
75% dilution fraction ~ MinDet
R² = 0.975
R² = 0.984
75% dilution fraction ~ Mean
R² = 0.96
R² = 0.989
75% dilution fraction ~ missForest
R² = 0.982
R² = 0.994
75% dilution fraction ~ msImpute
R² = 0.991
R² = 0.987
Estimated t-values
True t-values
-10
0
10
Strategy
Impute first
Normalize first

c

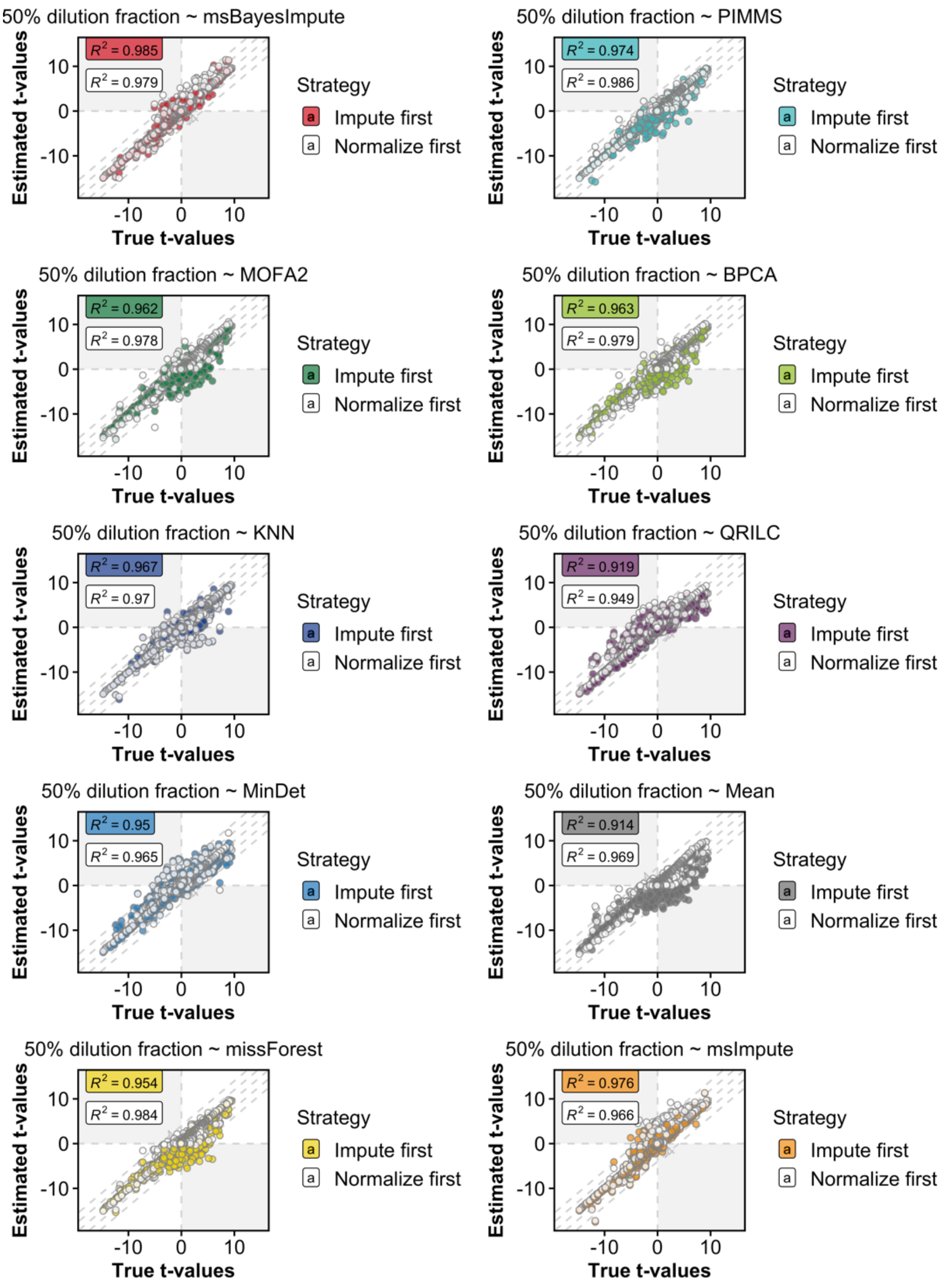

Response Figure 13: Assessing impute-first and normalize-first strategies across all MVI methods. (a) RMSD measuring the reconstruction using synthetic datasets across three missing rates. The comparison of two strategies can be seen for each MVI method. (b, c) Correlations of true t-values and estimated t-values after performing differential expression for two strategies across all methods except for DPC and proDA.

1. Brunner, A., Thielert, M., Vasilopoulou, C. *et al.* Ultra-high sensitivity mass spectrometry quantifies single-cell proteome changes upon perturbation. *Mol Syst Biol* **18**, MSB202110798 (2022). https://doi.org/10.15252/msb.202110798
2. Navarro, P., Kuharev, J., Gillet, L. *et al.* A multicenter study benchmarks software tools for label-free proteome quantification. *Nat Biotechnol* 34, 1130–1136 (2016). https://doi.org/10.1038/nbt.3685
3. Albrecht D, Kniemeyer O, Brakhage AA, Guthke R. Missing values in gel-based proteomics. Proteomics. 2010 Mar;10(6):1202-11. doi: 10.1002/pmic.200800576.
4. Mengbo Li, Gordon K Smyth, Neither random nor censored: estimating intensity-dependent probabilities for missing values in label-free proteomics, *Bioinformatics*, Volume 39, Issue 5, May 2023, btad200, https://doi.org/10.1093/bioinformatics/btad200
5. Li, M., Cobbold, S.A. and Smyth, G.K., 2025. Quantification and differential analysis of mass spectrometry proteomics data with probabilistic recovery of information from missing values. *bioRxiv*, pp.2025-04.
6. Webel, H. *et al.* (2024a) "Imputation of label-free quantitative mass spectrometry-based proteomics data using self-supervised deep learning," *Nature Communications*, 15(1), p. 5405. Available at: https://doi.org/10.1038/s41467-024-48711-5.
7. Webel, H., Perez-Riverol, Y., Nielsen, A.B. *et al.* Mass spectrometry-based proteomics data from thousands of HeLa control samples. *Sci Data* 11, 112 (2024b). https://doi.org/10.1038/s41597-024-02922-z

Point-by-point response to reviewers' final comments for "msBayesImpute: A Versatile Framework for Addressing Missing Values in Biomedical Mass Spectrometry Proteomics Data" by J.He et al.

Reviewer #1:
The reviewers have successfully addressed most of my comments, and I therefore basically recommend a publication. On a side note, the reviewer very much appreciates the very clear and structured form of the rebuttal letter and marking of changes made!

We sincerely thank the reviewer for the positive feedback and for their insightful comments.

There are only few remaining points to be clarified:
- L163-165: Computational time increases significantly for larger sets at non-linear rate. A one-liner to explain this behavior, e.g. linking to the complexity of the method, would help here to point at potential limits or future optimization directions.

The non-linear increase in runtime for larger datasets is mainly due to the increasing number of model parameters that must be inferred. As the sample size increases, more latent factor parameters and posterior distributions need to be estimated during both the initial and final training stages. In addition, estimating a larger number of latent factors during the initialization step becomes increasingly rate-limiting. For larger datasets, msBayesImpute may also adopt a more informative prior on the precision parameter to improve prediction stability and accuracy, which can further increase exploration time. Together, these factors increase memory usage and can lead to computational slowdown, particularly when memory becomes limiting. We have added a brief explanation of this behavior in the revised manuscript and now discuss it as a potential direction for future optimization **(Line 160-164)**: "*A non-linear increase in run time for larger datasets is expected, as more latent factors and posterior parameters need to be estimated during model training, increasing both computational complexity and memory usage. This highlights a potential direction for future optimization of msBayesImpute for very large-scale datasets through mini-batch training and parallelization.*"

- L544: \bar{U} can only be R^N\times D.
We have now fixed this notation in the revised manuscript **(Line 495)**

- Response Figure 2: Why do MinDet, QRILC, KNN and few others not show any run time values?
The reason that the run time values of MinDet, QRILC, KNN and feature-wise mean are invisible in the Response Figure 2 (main Figure 2k) is because they only took a few seconds to complete. To clarify this, we have added the following statement in Line 229-231: *By comparison, MinDet, QRILC, KNN, and Feature-wise Mean completed each job within seconds.*

- L248: typo "misssing"
- L 250: running time --> run time
- Suppl. Figure 7: typo "opitmized"

We have corrected those typos in the revised manuscript ("missing" in **Line 233**, "run time" in **Line 235**, "optimized" in Suppl. Figure 7).

Reviewer #2:
The authors have addressed all my comments. I would like to congratulate the authors on their hard work and recommend publication in Communications Chemistry.

I just have one minor comment, which I believe the authors can address during the proofing stage:
- Can the authors please double-check line 340 - 341: "...medians derived from msBayesImpute-imputed data were tightly centered around zero and exhibited..."? I think Fig. 5e,f shows the difference between the values from the original undiluted dataset and the imputed or unimputed values of the dataset where missingness was introduced. I think they mean to say that this difference is tightly centered around zero, so I think they'd rather mean to say something like this: "...medians derived from msBayesImpute-imputed data were tightly centered around their true value and exhibited..."

We sincerely thank the reviewer for the time spent on our work and the positive comment. We have rephrased the sentence accordingly in the main text in **Line 316-318**: "*Medians derived from msBayesImpute-imputed data were tightly concentrated around their true values and exhibited substantially lower variability compared to those obtained from the ten alternative methods or direct estimation from incomplete data (in Fig. 4e–f).*"

Reviewer #3:
The authors have addressed all my concerns in a satisfactory way.

We thank the reviewer for the positive feedback.

Reviewer report

Summary

In the work “msBayesImpute: A Versatile Framework for Addressing Missing Values in Biomedical Mass Spectrometry Proteomics Data”, Jiaojiao He *et al.* propose a new and improved method for imputing missing values in mass spectrometry-based proteomics.

In the analysis of MS-based proteomics data, missing values are a major concern. Imputation of such missing values is challenging, as different mechanisms contribute to missingness, and it is well known in the field that such missingness is partially random (“missingness at random”, MAR), and partially dependent on the abundance of the underlying peptide ions (“missingness not at random” MNAR). As noted by the authors, improper imputation will lead to biases in downstream statistical analysis. Omitting the imputation step in such a scenario does not solve this problem, as it may reduce statistical power and could also lead to bias. Additionally, many machine learning algorithms only work with complete data, which necessitates some form of imputation.

In their manuscript, the authors provide a comprehensive overview of numerous imputation methods that are already available. They highlight that in the most advanced of these approaches, the user must specify the fraction of data that is subject to MNAR, which is almost always unknown. They write that the advanced method MSImpute can automatically identify MAR and MNAR subsets, but is confined to two-group designs. However, this statement is no longer correct (see below for my comments). They also discuss new deep-learning-based methods such as PIMMS, which tends to outperform classic methods in larger cohorts but may underperform in smaller ones.

The authors propose MSBayesImpute, an elegant method that combines MAR- and MNAR-based imputation. Importantly, MSBayesImpute uses a data-driven approach to estimate the fractions of missingness attributed to MAR and MNAR. They first test MSBayesImpute on a synthetic dataset where missing values were introduced based on their method’s missingness assumptions. It is not really surprising that MSBayesImpute outperforms other methods in such a setting, but it is important to note that the missingness assumptions of MSBayesImpute are more realistic than those of most competing methods. The authors also leverage the synthetic datasets to show that their method can accurately recover sample-wise size factors, even when large fractions (up to 50%) of values are missing. They then introduce missing values in an existing HeLa dataset from Webel *et al.* (2024) using the independent approach from Lazar *et al.* (2016). In this fairer comparison, they again show MSBayesImpute’s superior performance. The authors then go the extra mile and generate

their own serial dilution experiments based on the proteomes of primary lung cancer tissues. Interestingly, they show that missing values introduced by diluting the samples display an inverse relationship between protein abundance and dropout probability, consistent with their proposed sigmoid-shaped dropout curve. And, they again demonstrate the superior performance of MSBayesImpute compared to the other methods when imputing the missing values of the diluted samples, using the undiluted samples as the ground truth.

MSBayesImpute is an elegant solution and relies on a solid theoretical foundation. It is implemented in both R and Python, and is available as a Shiny app and a Docker image, providing easy access to both bioinformaticians and scientists who prefer a graphical user interface. The authors benchmark their method extensively against eight state-of-the-art missing value imputation methods. I therefore completely agree with the words of the authors: **"Together, these features position msBayesImpute as a versatile and robust tool for enhancing the reliability and interpretability of label-free MS proteomics in biomedical research."**

I still have one major comment and several minor comments. The major comment relates to the fact that, as of 2025, MSImpute seems no longer to be confined to a two-sample experimental design. The minor comments are mainly suggestions to improve the readability even further, and are an expression of my excitement about the work. **I would like to congratulate the authors not only on providing an excellent software tool that is grounded in solid statistical theory and addresses a real need in the field, but also on the robustness of the analyses and the clarity of the manuscript. In my opinion, this is a high-quality scientific work, and I highly recommend publication in *Communications Chemistry*, provided that the authors address my comments below.**

Major comment

1. On multiple occasions, the authors write that MsImpute is limited to two-group experimental designs. See e.g. Line 82: "Another approach, MsImpute (Hediyeh-Zadeh, Webb and Davis, 2023), can automatically identify protein subsets adhering to MAR or MNAR mechanisms, though its application is confined to two-group experimental designs." Line 267: "We next compared msBayesImpute with nine other MVI methods, including MsImpute, which models MAR/MNAR mixtures but is restricted to two-group experimental designs (e.g., tumor vs. tumor-free)." At present, this statement is no longer correct. The 2023 manuscript that describes MSimpute (Hediyeh-Zadeh *et al.*, *MCP*) states "Note that although 2-group designs were considered in the DA benchmark analyses covered here, the method is applicable to complex experimental designs (*e.g.*, time-series, multiple conditions, combination of groups and time course, etc.) if the experimental groups of interest can be

discretized." Since March 2025, their GitHub page now deprecates the use of the "group" argument in favor of a design matrix, which indicates that MsImpute can now also handle more complex experimental designs. Hence, unless there is another reason, it would be a good idea to include MsImpute also in the analyses in Fig. 2 and Fig. 3.

Minor comments

2. Fig.1: I would somewhere specify that (I assume): ρ is the inflection point, and ζ is the scale parameter of the dropout function. I would optionally also specify that $\boldsymbol{\sigma}^2$ is the variance of the Gaussian function (even if that is perhaps more obvious).

3. A suggestion for clarity. On line 163: the authors write: "Each condition was repeated on five datasets with the same missingness profile." To me, this sentence came as a surprise, and I could not immediately figure out which datasets the authors were talking about. Only by re-reading and checking Fig. 2d, it became clear to me that these are five simulations. To make this clearer, I would suggest to re-write the sentence on line 156 for example as: "We next benchmarked msBayesImpute against eight commonly used missing value imputation (MVI) methods under varying missing rates (10%, 30%, 50%**; five simulated datasets each) with 50 samples per dataset.**" (or anything else that makes it clear that there are five simulations per missingess rate). I would then simply drop the sentence "Each condition was repeated on five datasets with the same missingness profile." from line 163.

4. Fig. 2a: the authors simulated datasets with 20, 100 and 200 samples. In practice, datasets with 100 or 200 samples are usually already quite large experiments. I was just curious what happens in a very small experiment of, say, 6 samples (i.e. the "classic" 3 control vs 3 treated samples design)? Could the authors add a simulation with 6 samples too, to show the behavior of msBayesImpute in very small experiments?

5. In Fig. 2b, the authors show coefficients of determination (R^2), which I assume are based on an ordinary linear regression. I was wondering why the authors are plotting the diagonal line instead of the actual regression line. Would the latter not be more informative?

6. Line 179: I would specify that this is a **95%** confidence interval.

7. Fig. 2(f,g), in line 180, I would also specify in the legend "derived from ground truth data versus matrices with **~30%** missing values".

8. On line 192, the authors write: "To assess this effect, we simulated datasets with ~30% missing values." I was wondering if these are the same datasets with 30% missing values mentioned on line 157? If yes, perhaps the authors could briefly clarify this in the text. If not, could they specify in the text with what method the missing values were introduced?

9. Fig. 3d: it is not immediately clear to me how over- and underestimation are calculated. Could the authors clarify this? As a differences closer to zero are better and differences away from zero are worse, I would highlight the 0-level with a horizontal line to clarify this in the figure. I would also clarify in the figure and/or in the figure legend what "differences" means exactly.

10. Line 278: the authors write: "MsImpute yielded comparably accurate normalization, whereas KNN and unimputed data performed the poorest." I was wondering if the authors actually performed the subsequent normalization or if this is based on MsImpute having a low bias in Fig. 4e and f? For normalization purposes, I could imagine that the most important thing is not the bias, but the variance of the differences shown in Fig. 4e,f. Imagine all the sample median abundances are strongly biased, but with a fixed, constant bias, then I would imagine the subsequent differential abundance analysis will not be biased, because when comparing two samples, the constant biases will cancel each other out. I could be wrong here, but if the authors agree that my interpretation is correct, perhaps they should either (1) reword to specify that they are talking about the variability of the differences, or (2) reword to avoid the word "normalization", or (3) if possible perform some kind of analysis to show that normalization is effectively more accurate here.

11. Purely out of curiosity, and I do not necessarily expect the authors to address this in the manuscript, as it is somewhat out of scope, but I was quite shocked to see in Fig. 5 a and b that the actual FDR remains overall quite stable and, for msBayesImpute, even decreases, over a desired FDR that ranges from 0 to 25%. What do you think would be the explanation for this phenomenon?

12. In the section from lines 290 – 302, the authors compare DE analyses based on different imputation methods, as well as the standard proDA workflow. However, I think they should explicitly mention in the main text and not only in the Methods

section that they used limma for DE analysis for all methods except proDA (which has its own dedicated workflow). Otherwise, I am left wondering from which analysis the FDR values were derived.

13. In equation (9), is "N+" a positive half Gaussian distribution?

14. The authors are very thorough in their discussion of the limitations of msBayesImpute, which I really appreciate. They write: "Its use of a variational Bayesian framework means that uncertainty quantification is generally underestimated (Blei, Kucukelbir and McAuliffe, 2017), which could limit its integration into downstream statistical inference or modelling." Out of curiosity, I was wondering if the software would allow small perturbations of the hyperparameters, such as those of the gamma distribution of the variance (100, 10) and/or the rate z_d (200,10) and/or if changing the seed produces different results? This would then allow users to perform multiple imputation. Could such an approach mitigate some of these problems? If yes, maybe it is also an interesting future direction to mention in the discussion section? If not, there is probably no need to discuss this.

15. Line 617, the authors write: "For the semi-synthetic and experimental data, a log transformation of base 2 was applied to the spectra counts prior to imputation." This comes as a surprise to me. Throughout the manuscript, I assumed the authors worked with spectral intensities (see e.g. line 274). It is known that intensity-based approaches are more powerful than count-based approaches in differential abundance analysis for MS-based proteomics. Could the authors please clarify whether they used summarized protein-level intensities, spectral count data, or something else (e.g. ion-level or peptide level intensities, peptide counts, …)?

Spelling/grammar suggestions

- Line 542: "One method generates missing values with the probabilistic dropout function on which msBayesImpute **is** based,"
- Line 412: "with the ~~length of~~ D" -> "with length D"
- Line 548: "TMT- or SILAC labelled MS data." -> "TMT- or SILAC-labelled MS data."
- Line 557: "…missingness and to avoid bias during benchmarking." -> "…missingness. It also avoids bias during benchmarking."
- Line 561: "function as missing rate" -> "function, as missing rate"
- Line 564: "For benchmarking, a semi-synthetic dataset, where…" -> "For benchmarking, **we used** a semi-synthetic dataset where…"

- Line 588: “For sample preparation two slices each” -> “For sample preparation**,** two slices each”
- Line 674: “which was proven to outperform other commonly used normalisation methods in differential expression tasks~~, such as the median normalisation approach~~ (Valikangas, Suomi and Elo, 2016).” -> “which was proven to outperform other commonly used normalisation methods**, such as the median normalisation approach**, in differential expression tasks (Valikangas, Suomi and Elo, 2016).”
